# Discovery of AD258 as a Sigma Receptor Ligand with Potent Antiallodynic Activity

**DOI:** 10.1021/acs.jmedchem.3c00959

**Published:** 2023-08-03

**Authors:** Maria Dichiara, Francesca Alessandra Ambrosio, Sang Min Lee, M. Carmen Ruiz-Cantero, Jessica Lombino, Adriana Coricello, Giosuè Costa, Dhara Shah, Giuliana Costanzo, Lorella Pasquinucci, Kyung No Son, Giuseppe Cosentino, Rafael González-Cano, Agostino Marrazzo, Vinay Kumar Aakalu, Enrique J. Cobos, Stefano Alcaro, Emanuele Amata

**Affiliations:** † Dipartimento di Scienze del Farmaco e della Salute, Università degli Studi di Catania, Viale Andrea Doria 6, 95125 Catania, Italy; ‡ Dipartimento di Medicina Sperimentale e Clinica, Università degli Studi “Magna Græcia” di Catanzaro, Campus “S. Venuta”, Viale Europa, 88100 Catanzaro, Italy; § Department of Ophthalmology and Visual Sciences, University of Illinois at Chicago, 1905 W Taylor St, Chicago, Illinois 60612, United States; ∥ Departamento de Farmacología e Instituto de Neurociencias, Facultad de Medicina, Universitad de Granada e Instituto de Investigación Biosanitaria de Granada ibs.GRANADA, Avenida de la Investigación, 18016 Granada, Spain; ⊥ Dipartimento di Scienze della Salute, Università “Magna Græcia” di Catanzaro, Campus “S. Venuta”, 88100 Catanzaro, Italy; # Net4Science Academic Spin-Off, Università “Magna Græcia” di Catanzaro, Campus “S. Venuta”, 88100 Catanzaro, Italy; ∇ Department of Ophthalmology and Visual Sciences, University of Michigan, 1000 Wall Street, Ann Arbor, Michigan 48105, United States

## Abstract

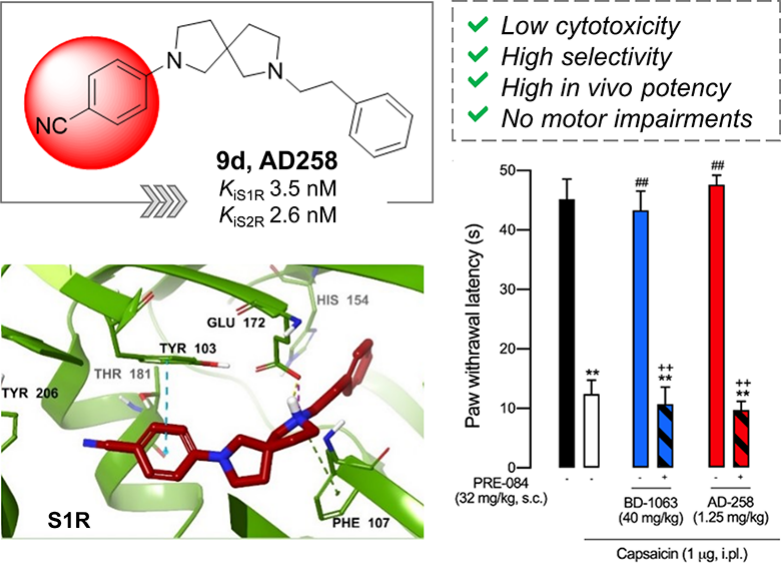

The design and synthesis of a series of 2,7-diazaspiro[4.4]nonane derivatives as potent sigma receptor (SR) ligands, associated with analgesic activity, are the focus of this work. In this study, affinities at S1R and S2R were measured, and molecular modeling studies were performed to investigate the binding pose characteristics. The most promising compounds were subjected to *in vitro* toxicity testing and subsequently screened for *in vivo* analgesic properties. Compound **9d** (**AD258**) exhibited negligible *in vitro* cellular toxicity and a high binding affinity to both SRs (*K*_i_S1R = 3.5 nM, *K*_i_S2R = 2.6 nM), but not for other pain-related targets, and exerted high potency in a model of capsaicin-induced allodynia, reaching the maximum antiallodynic effect at very low doses (0.6–1.25 mg/kg). Functional activity experiments showed that S1R antagonism is needed for the effects of **9d** and that it did not induce motor impairment. In addition, **9d** exhibited a favorable pharmacokinetic profile.

## Introduction

Pain is a serious health problem affecting the lives of millions of people around the world, with significant costs to the healthcare system.^1^ In many cases, current analgesics provide only modest efficacy and are limited by several side effects preventing long-term use. Chronic pain can be complicated by or co-existent with psychiatric morbidities, such as depression and post-traumatic stress disorder. These co-morbidities can have important effects on responses to therapy and quality of life. Given the high societal burden of chronic pain, many efforts have been pursued in finding novel analgesic candidates with favorable side effect profiles and novel mechanisms of action. However, the identification of safe and efficacious agents continues to be an unmet social need and a significant challenge for the scientific community.^2^

Sigma receptors (SRs) are a unique receptor class involved in several biological and pathological conditions.^3^ Two subtypes are distinguished and termed sigma-1 receptor (S1R) and sigma-2 receptor (S2R), having different structural, biological functions, and pharmacological profiles. S1R has been purified and cloned in several species and is well characterized as a chaperone protein at the mitochondrion-associated membrane (MAM) of the endoplasmic reticulum (ER) where it forms a complex with the binding immunoglobulin protein (BiP).^4^ Once triggered, S1R provokes its dissociation from BiP and translocation to the plasma membrane where it networks with client proteins such as G protein-coupled receptors and ion channels.^5−10^ The S1R is highly expressed in both the central and peripheral nervous system and exerts effects on these areas of great relevance in neuroprotection, neuroinflammation, neurotransmission, and neuroplasticity.^11^ Several pieces of evidence support the modulatory role of S1R in the treatment of pain, primarily centered on a phenotype in S1R knockout (KO) mice of pain attenuation and on the antinociceptive effect induced by S1R antagonists.^12^ The selective S1R antagonist E-52862 (S1RA) has shown effectiveness in phase 2 clinical trials for the management of neuropathic pain of a variety of causes, including chemotherapy-induced neuropathy and potentiation of opioid receptor-mediated analgesia in the postoperative period after abdominal hysterectomy.^13,14^

S2R is a poorly understood protein whose identification dates to 1990, which has attracted considerable interest as target for the treatment of neurological diseases and cancer.^15^ In 2017, S2R was identified as an endoplasmic reticulum-resident transmembrane protein (TMEM97). TMEM97 is thought to play a role in cholesterol homeostasis and function as a modulator of the sterol transporter Niemann–Pick disease type C1.^16^ It is thought that S2R is trafficked through multiple subcellular structures including the ER, lysosomes, and mitochondria, and modulation of the S2R can result in numerous tissue and cell-specific cell and molecular outcomes such as the release of intracellular Ca^2+^, dopaminergic transmission, and neurodegeneration as well as the pathogenesis of cancer and neurological disorders.^17^

**Figure 1:**
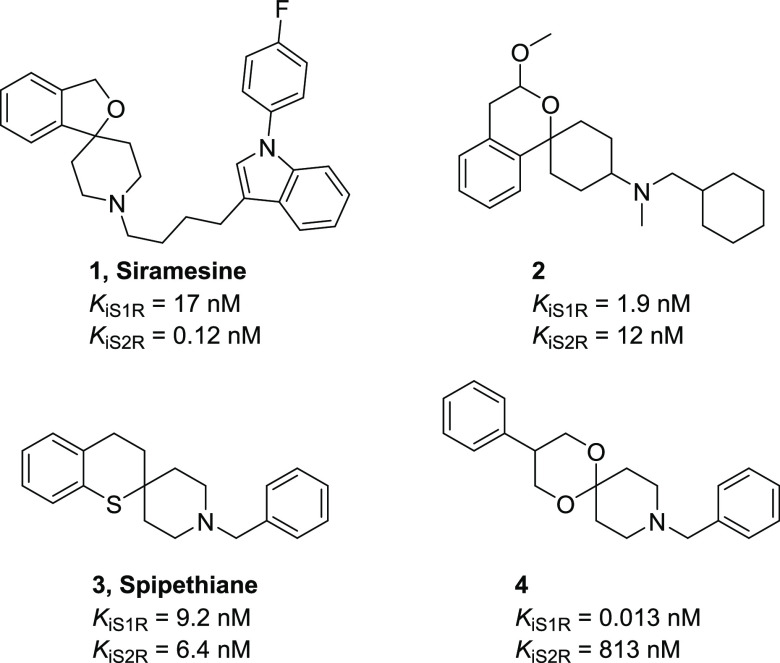
Structures of selected spirocyclic SR ligands.

Spirocyclic compounds have gained increasing interest in the development of bioactive compounds and contribute to a variety of approved drugs and drug candidates. The introduction of a spirocyclic moiety in a molecule grants a peculiar spatial arrangement that may influence important parameters, such as potency, selectivity, and physicochemical properties.^18^ A few spirocyclic ring systems used as conformationally restricted scaffolds with affinity towards S1R and S2R are described in the literature (Figure 1). The 3*H*-spiro[isobenzofuran-1,4′-piperidine] derivative siramesine (**1**) has been reported to have high affinity and selectivity to S2R.^19^ The spiro[cyclohexane-1,1′-isochromane] derivative (**2**), spipethiane (**3**) bearing a spiro[piperidine-4,2′-thiochromane] moiety, and the 1,5-dioxa-9-azaspiro[5.5]undecane derivative (**4**) resulted in compounds with exceptional ability to bind the SRs.^20,21^ Recently, a series of spirocyclic compounds as binders of S1R have been reported, although all the described compounds have shown human and mouse liver microsomal high intrinsic clearance.^22^

In this study, we report the development of 2,7-diazaspiro[4.4]nonane derivatives where – consistent with the SR pharmacophore requirements – the amino moiety has been decorated with hydrophobic groups at different distances. In contrast to the reported SR ligands with spirocyclic structure, which often bear a six-membered ring, the spiroamine 2,7-diazaspiro[4.4]nonane reported here retains the structural features of a pyrrolidine ring substituted in the 3-position by an aminomethyl group, although less flexible. We have synthesized and tested structure–activity relationship (SAR) studies for 19 compounds. Molecular modeling analysis was carried out to deeply analyze the binding mode and the interactions established between the designed compounds and SRs. Finally, we tested the most promising compounds using *in vitro* toxicity assays and subsequent screening for activity in an *in vivo* model of sensory hypersensitivity. To investigate the possibility that the observed *in vivo* effects could be associated with interference in motor coordination and thus with the response of mice in the nociceptive-related behavioral tests, motor performance was also measured using a rotarod test.

## Results and Discussion

### Chemistry

**Scheme 1:**
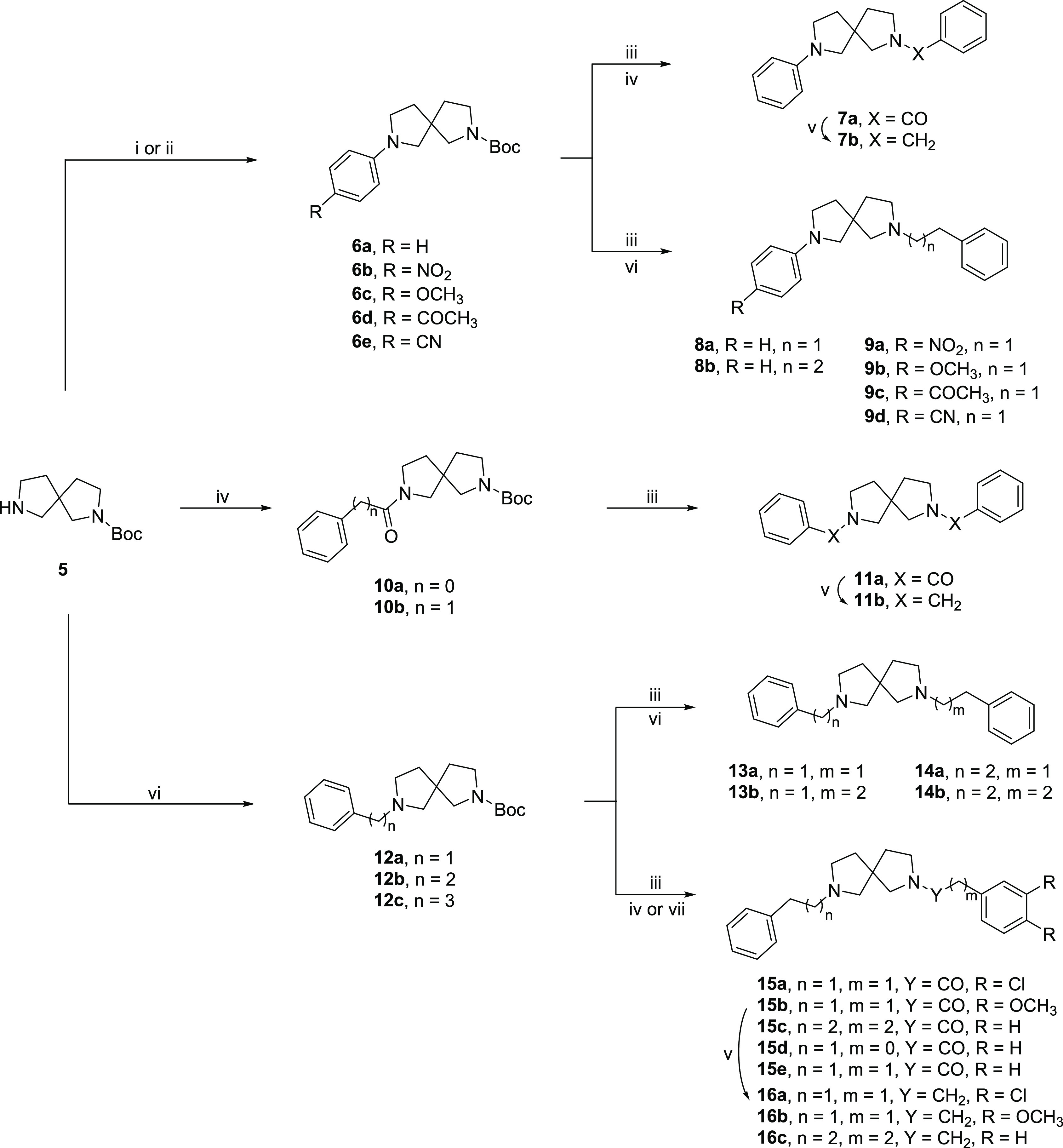
Synthetic Strategy for the Preparation of Target Compounds Reagents and conditions: (i) *p*-iodobenzene derivative, Pd_2_(dba)_3_, SPhos, *t*-BuOK, toluene, 100 °C, on (Procedure A); (ii) *p*-chlorobenzonitrile, K_2_CO_3_, DMSO, 120 °C, on (Procedure B); (iii) TFA, CH_2_Cl_2_, rt, 4 h; (iv) acyl chloride, TEA, CH_2_Cl_2_, rt, 2 h (Procedure D); (v) LiAlH_4_, THF, rt, N_2_ (Procedure F); (vi) alkyl bromide, K_2_CO_3_, ACN, 60 °C, on (Procedure B); (vii) EDC, HOBt, DMF, rt, 6 h (Procedure G).

Scheme 1 depicts the general synthesis of the racemic spirocyclic compounds described in this work.

Buchwald–Hartwig amination with appropriate *p*-iodobenzene substituents on *tert*-butyl-2,7-diazaspiro[4.4]nonane-2-carboxylate (**5**), provided intermediates **6a**–**d**,^23^ whereas compound **6e** was synthesized through nucleophilic aromatic substitution with *p*-chlorobenzonitrile. After *N*-Boc deprotection, all the intermediates underwent acylation or alkylation reactions. Intermediate **6a** was converted into the amide derivative **7a**, through nucleophilic acyl substitution with benzoyl chloride, which was then reduced to amine **7b** with LiAlH_4_.^24,25^

Conversely, derivatives **6b**–**e** were employed in alkyl substitution reactions to give corresponding final derivatives **8a**,**b** and **9a**–**d**. Intermediates **10a**,**b** were obtained from the reaction with opportune acyl chloride. After *N*-Boc deprotection, **10a** underwent nucleophilic acyl substitution with benzoyl chloride to give amide **11a**, which was then reduced to amine **11b**. Intermediates **12a**,**c** were obtained from **5** by alkylation with opportune alkyl bromide and deprotected with TFA. The alkylation with (2-bromoethyl)benzene or 1-bromo-3-phenylpropane of compound **12a** gave compounds **13a**,**b** and **14a**,**b**. Conversely, amine derivatives **12b**,**c** were conjugated with opportune acids in coupling reactions to give compounds **15a**,**c**, which underwent further reduction with LiAlH_4_ to give amines **16a**–**c**.^26^ Compounds **15d**,**e** were obtained by acyl substitution.

### SAR Studies

Once synthesized, compounds were subjected to radioligand binding assays for the evaluation of affinity at both S1R and S2R. Compounds were evaluated in rat liver homogenates using [^3^H]-(+)-pentazocine and [^3^H]DTG as radioligands for S1R and S2R, respectively. Nonspecific binding for S1R was measured in the presence of 10 μM unlabeled (+)-pentazocine and in the presence of 10 μM unlabeled DTG for S2R assays. Moreover, since a selective S2R radioligand is not available, [^3^H]DTG was used in the presence of an excess of (+)-pentazocine to mask the S1R sites. The results are summarized in Tables 1 and 2.

**Table 1:**
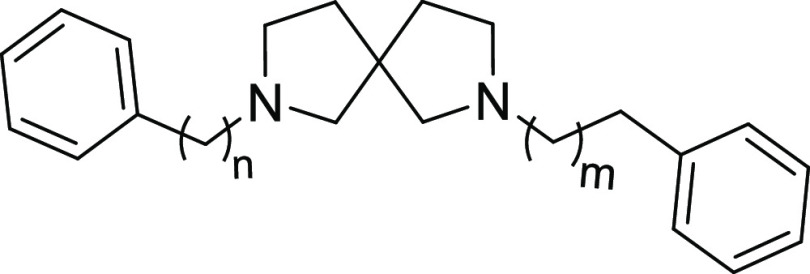
S1R and S2R Binding Assays

			*K*_i_ (nM) ± SD^a^		
ID	*n*	*m*	S1R	S2R	*K*_iS2R_/*K*_iS1R_
**7b**	0	0	10 ± 1.4	40 ± 4.4	4.0
**8a**	0	1	6.5 ± 0.9	95 ± 9.5	14.6
**8b**	0	2	8.6 ± 1.4	85 ± 13	9.9
**11b**	1	0	1.8 ± 0.4	14 ± 1.6	7.8
**13a**	1	1	2.5 ± 0.4	20 ± 3.1	8.0
**13b**	1	2	11 ± 2.0	12 ± 2.3	1.1
**14a**	2	1	2.1 ± 0.4	13 ± 2.2	6.2
**14b**	2	2	11 ± 1.4	18 ± 3.4	1.6
**16c**	3	2	36 ± 5.7	7.7 ± 0.7	0.2
**(+)-PTZ**			4.3 ± 0.5	1465 ± 224	
**DTG**			124 ± 19	18 ± 1	
**Haloperidol**			2.6 ± 0.4	77 ± 18	
**BD1063**			14 ± 2.7	204 ± 31	

a Each value is the mean ± SD of at least two experiments performed in duplicate.

**Table 2:**
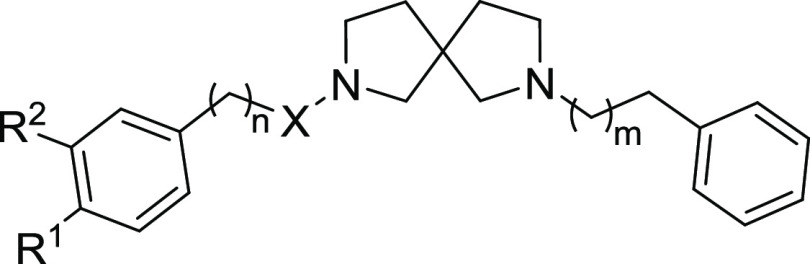
S1R and S2R Binding Assays for Targeted Compounds

						*K*_i_ (nM) ± SD^a^		
ID	R^1^	R^2^	X	*n*	*m*	S1R	S2R	*K*_iS2R_/*K*_iS1R_
**9a**	NO_2_	H		0	1	0.76 ± 0.17	12 ± 2.9	15.8
**9b**	OCH_3_	H		0	1	6.2 ± 1.8	12 ± 1.2	1.9
**9c**	COCH_3_	H		0	1	3.0 ± 0.46	10 ± 1.7	3.3
**9d**	CN	H		0	1	3.5 ± 0.7	2.6 ± 0.6	0.7
**15a**	Cl	Cl	CO	1	1	7.5 ± 1.0	24 ± 3.6	3.2
**15b**	OCH_3_	OCH_3_	CO	1	1	435 ± 35	736 ± 267	1.7
**15d**	H	H	CO	0	1	209 ± 18	57 ± 3.3	0.3
**15e**	H	H	CO	1	1	79 ± 3.8	99 ± 16	1.3
**16a**	Cl	Cl	CH_2_	1	1	4.2 ± 0.8	11 ± 2.1	2.6
**16b**	OCH_3_	OCH_3_	CH_2_	1	1	25 ± 5.3	23 ± 3.5	0.9

a Each value is the mean ± SD of at least two experiments performed in duplicate.

The first set of compounds was prepared and tested to evaluate the variation in affinity for the targets of the 2,7-diazaspiro[4.4]nonane followed by substitution at the nitrogen with plain phenyl rings located at different distances as given by opportune spacers. All the synthesized compounds demonstrated high affinity for the S1R with *K*_i_ ranging from 1.8 to 11 nM and different ranges of selectivity over S2R based on substituents (Table 1).

The benzyl derivative **7b** demonstrated a slightly lower affinity at S1R and a higher affinity for the S2R, thus showing a lower preference for S1R with respect to **8a** bearing a phenethyl substituent on the nitrogen.

Compound **8b**, with a phenpropyl group, shares a similar profile to **8a** although with a slightly worse affinity and selectivity. The reinstatement of two basic nitrogens gives the symmetric compound **11b** and derivatives **13a** and **13b**, with the two of them showing higher affinity at S1R compared to **8a** together with an improvement of the affinity for the S2R and a sequential reduction of selectivity. The last modification resulted in the symmetric compound **14a** and derivative **14b** bearing a phenethyl group. The elongation to a three-carbon chain as in **14b** has provided a reduction in affinity for both subtypes. It must be noted that compound **13b**, having a phenethyl and a benzyl group, belongs also to this subset of compounds and it shares a similar profile to **14b**. The symmetric derivative **16c** shows a reduction of the S1R affinity when compared to **14a** and **14b**, with an improvement of selectivity over S2R.

Overall, the best compounds in terms of S1R affinity are those bearing benzyl or phenethyl substituents on both sides of the molecule. Nevertheless, compound **8a** shows a high affinity for S1R together with a moderate selectivity for S2R. From this first round of testing, chemical variations of **8a**, **13a**, and **14a** were designed to better understand the influence of the nitrogen atoms, the chain length, and aromatic ring substituents with regard to affinity and selectivity (Table 2). Derivatives of **8a**, compounds **9a**–**d**, were designed to add further binding opportunity with the targets and to dig around selectivity. Compound **9d** (AD258), with a *p*-CN-phenyl group, has shown a high affinity for both receptors with *K*_i_ values below 4 nM. The introduction of a nitro group in the *para* position (**9a**) improved affinity for both SRs. Notably, compound **9a** is the only compound in the series demonstrating an affinity for S1R below 1 nM.

Compound **9c** having a *p*-CH_3_CO-phenyl group showed a similar *K*_iS1R_ to **9d** and a slightly lower affinity for S2R. Compound **9b** bearing a *p*-CH_3_O-phenyl group is the worst binder of the series with *K*_i_ for both receptors in the 10 nM range. The modifications made have given products with an outstanding affinity for S1R together with improved affinity for the S2R, thus providing compounds with peculiar affinity profiles. Variations of **13a** as in compound **15d** determined an inversion of the SR profile with preferential affinity for S2R as compared to S1R. However, the presence of the amide function in compound **15d** reduces the affinity for both SR subtypes. Similar to **15d**, the insertion in **14a** of an amide group led to compound **15e** with lower affinity on both SRs. The presence of the chlorine atoms in positions 3 and 4 of the aromatic ring as in **15a** takes back to low nanomolar affinity without significant preference. The corresponding amine derivative **16a** showed an improvement in affinity for both subtypes. The presence of two methoxy groups as in **15b** shifted the affinity towards negligible *K*_i_ values. The reduction of the amide function into the corresponding amine derivative **16b** restored a two-digit nM affinity for both subtypes.

Overall, the 2,7-diazaspiro[4.4]nonane moiety has provided ligands with optimal features for SR binding. Compounds **8a** and **9a** showed high-affinity at S1R or S2R with selectivity in the 15 fold range. Conversely, compound **9d** resulted in a mixed high-affinity ligand for both receptor subtypes. Detailed mechanistic studies allowed us to understand the binding interactions between the ligands and SRs. With this in mind, we decided to further investigate the effects of these molecules in additional in vitro and in vivo models.

### Molecular Modeling

We carried out molecular modeling studies to explore the binding mode of the synthesized ligands and their interactions with SRs.

The 5HK1 and 7M95 crystal structures for both receptors were retrieved from the Protein Data Bank. The two structures were selected among others for their high resolutions and the completion of the full-length models. The bovine model of the S2R was mutated into human by manually modifying only the different residues. The two structures were subjected to 100 ns molecular dynamics simulations to relax the systems and obtain multiple representative conformations to subsequently assess and identify the most efficient ones at recognizing active compounds. For the latter purpose, clusterization and subsequent validation of the representatives of the most populated clusters were carried out. The docking of all the stereoisomers of the 19 compounds of interest was hence performed on the structures with the highest enrichment capacity.

The docking results showed that for each compound, all the stereoisomers are well accommodated within the S1R and S2R binding pockets and they are associated with excellent theoretical binding affinity (see Table S1).

Considering the S1R, all the compounds interact with the pivotal residues of the S1R binding pocket, and the pose of the protonated nitrogen of the 2,7-diazaspiro[4.4]nonane core is very peculiar, being near the carboxylic moiety of Glu172, a highly conserved amino acid closely positioned to the center of the cavity and crucial for the ligand binding.^27^ It is also worthy of note that all the compounds establish hydrogen bonds or salt bridge interactions with the carboxylic group of that residue. In some compounds, the protonated nitrogen is also engaged in a π–cation interaction with Phe107 while the other nitrogen atom, when charged, is in most cases involved in a π–cation interaction with Tyr103. The aromatic non-substituted ring of most of the **8a** derivative compounds is engaged in π-stacking interactions with Phe133 or His154 while the substituents present on the other phenyl ring are turned toward Tyr206 with which they make several hydrophobic contacts. Moreover, one oxygen atom of the nitro group of **9a** is engaged in an aromatic H-bond interaction with Tyr206.

**Figure 2:**
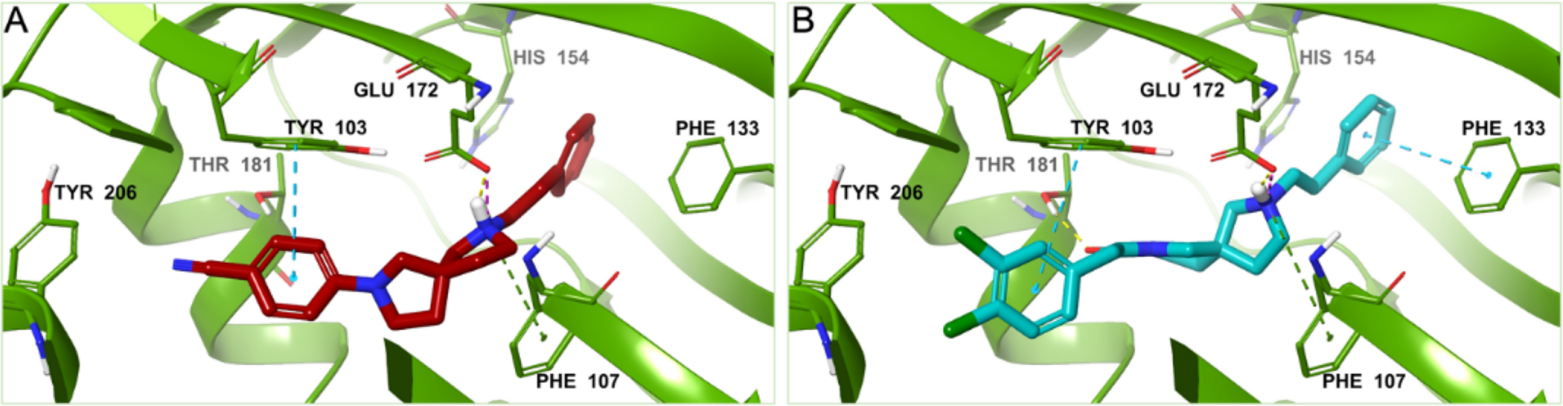
3D representation of the best scoring enantiomer of (A) **9d** and (B) **15a** docked in the binding pocket of the S1R representative conformation obtained after clusterization of the MD trajectories (PDB ID 5HK1). The ligands are shown as burgundy and teal sticks, respectively. The S1R is shown as a green cartoon, and the enzyme residues participating in pivotal interactions with the ligands are reported as green carbon sticks. Salt bridges, π–π stacking, π–cation, hydrogen bonds, and aromatic H-bonds interactions are respectively represented by magenta, azure, green, yellow, and cyan dashed lines.

Furthermore, compounds **9a**, **9d**, **15a**, **16a**, **15b**, and **16b** present different substituents on one of the phenyl rings – a nitro group for **9a**, a *p*-CN-phenyl group for **9d** (Figure 2A), a chlorine for **15a** (Figure 2B) and **16a**, and methoxy groups for **15b** and **16b** – which are accommodated in the proximity of Tyr206, with which they make several hydrophobic contacts. Considering compounds **15a**, **15b**, **15e**, and **15d**, they present an amide function, and the oxygen of this group makes a hydrogen bond with the side chain of Thr181.

Finally, all the ligands are well accommodated among the hydrophobic amino acids that cover the interior surfaces of the binding cavity and they also establish different kinds of interactions, such as π–cation, π–π stacking, and hydrophobic interactions with His154, Phe107, Tyr103, Phe133, and Tyr 206, and hydrogen bonds with Thr181.

Regarding the S2R receptor, all the studied compounds establish different kinds of interactions with at least one of the conserved acid residues Asp29, Asp56, and Glu73.^17^ The protonated nitrogen of the 2,7-diazaspiro[4.4]nonane core of the ligands is engaged in salt bridges or hydrogen bonds with the conserved Asp29 and in some cases, it is also involved in π–cation interactions with Tyr150. The other charged nitrogen of the two symmetric compounds **14a** and **14b** is involved in a π–cation interaction with Tyr147; meanwhile, in the case of compound **16b**, the second charged nitrogen is engaged in a π–cation interaction with a salt bridge with Tyr50 and Asp56, respectively.

**Figure 3:**
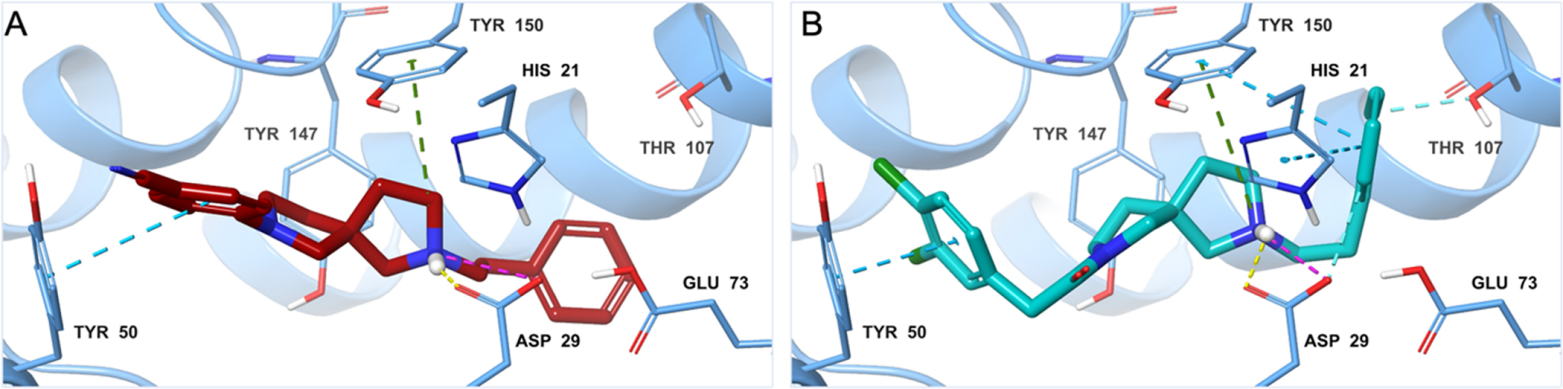
3D representation of the best scoring enantiomer of (A) **9d** and (B) **15a** docked in the binding pocket of the S2R representative conformation obtained after clusterization of the MD trajectories (PDB ID 7M95). The ligands are shown as burgundy and teal sticks, respectively. The S2R is shown as an azure cartoon, and the amino acids engaged in pivotal contacts with the ligands are represented as azure carbon sticks. Salt bridges, π–π stacking, π–cation, hydrogen bonds, and aromatic H-bonds interactions are, respectively, represented by magenta, azure, green, yellow, and cyan dashed lines.

The substituents on the phenyl ring of the derivatives of **8a** are projected toward Tyr50 establishing several hydrophobic contacts and, in detail, compounds **9b**, **9c**, and **9d** (Figure 3A) make also π–π interactions with this residue through their benzyl substituted ring, while the unsubstituted phenyl ring of compound **9a** is engaged in an aromatic H-bond interaction with Glu73.

Moreover, π-stacking interaction between the benzyl ring and Tyr50 was observed for compounds **7b**, **8b**, **13a**,**b**, **14a**, and **14b**. The substituents on one of the phenyl rings of compounds **15a**, **16a**, and **15b** are accommodated in the proximity of Tyr50, and these groups – chlorine for **15a** and **16a**, and methoxy groups for **15b** – make several hydrophobic contacts with this residue. In addition, the substituted phenyl ring of compounds **15a** (Figure 3B) and **16b** is engaged in a π-stacking interaction with Tyr50.

Finally, all the ligands form many hydrophobic interactions with the hydrophobic amino acids of the binding pocket.

### Toxicity and Tolerability

**Figure 4:**
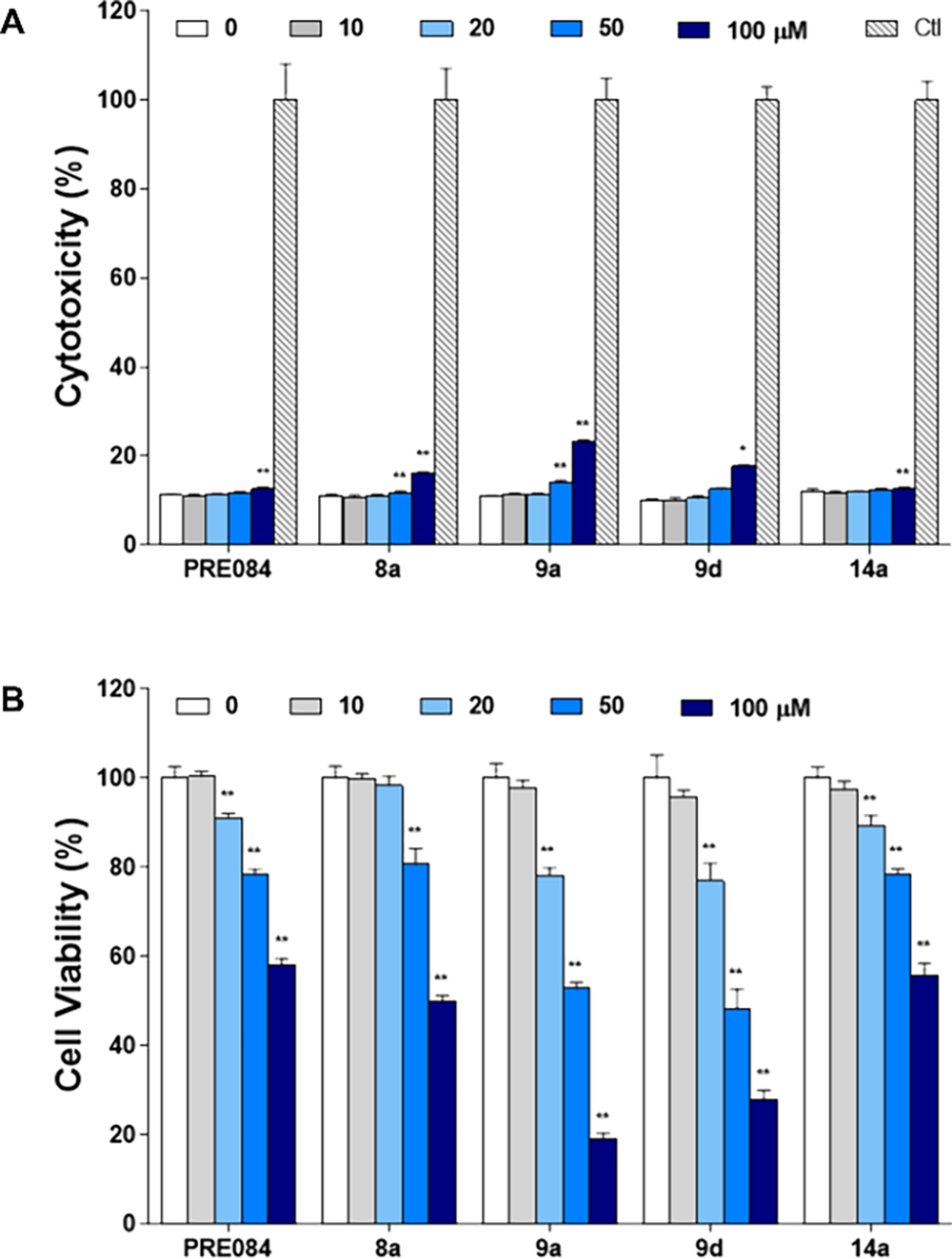
Effects of five compounds on cytotoxicity and cell viability in HCE cells. (A) Cytotoxicity in treated cells was evaluated using the LDH assay and (B) cell viability was measured using the MTT assay. The results are displayed in percentage of control samples. Each value represents the mean ± S.D. and is representative of results obtained from three independent experiments. *p < 0.05, **p < 0.01 compared to 0 μM.

Compounds **8a**, **9a**, **9d**, and **14a** were further investigated for their potential to cause cellular toxicity by metabolic changes and phenotypic effects in human corneal epithelial cells (HCE) (Figure 4). The cytotoxicity profile against HCE was assessed using the lactate dehydrogenase (LDH) assay and the 3-(4,5-dimethylthiazol-2-yl)-2,5-diphenyl-2*H*-tetrazolium bromide (MTT) assay (Figure 4A,B, respectively). These assays are standard measures of cellular toxicity and metabolism respectively.^28^

Similarly, to the selective S1R agonist PRE-084, all the tested derivatives showed low toxic activity, indicating that compounds in this series are quite well tolerated also at higher concentrations. Below concentrations of 50 μM there was no statistically significant cytotoxicity for most compounds tested. At very high concentrations of 50–100 μM, there was a significant but small increase of LDH release for all tested compounds with **9a** exhibiting the highest cytotoxicity (23%) (Figure 4A). Subsequently, the capacity of the derivatives to modulate cell viability was measured by MTT assay (Figure 4B). The reference standard S1R agonist PRE-084 showed a small but statistically significant reduction in cell viability above 20 μM concentrations. At very high concentrations (100 μM), this reached an effect size of 58% reduction in cell viability. Compounds **8a** and **14a** showed similar profiles to PRE-084 with cell viability reduction with statistically significant but very modest increases in metabolic depression above 20 μM but only demonstrating larger reductions in cell viability at 100 μM, 50% and 56% at 100 μM, respectively. Finally, compounds **9a** and **9d** exhibited similar patterns to the other compounds tested but reached slightly higher toxicity at the highest tested concentrations (100 μM).

### In Vivo Studies

**Figure 5:**
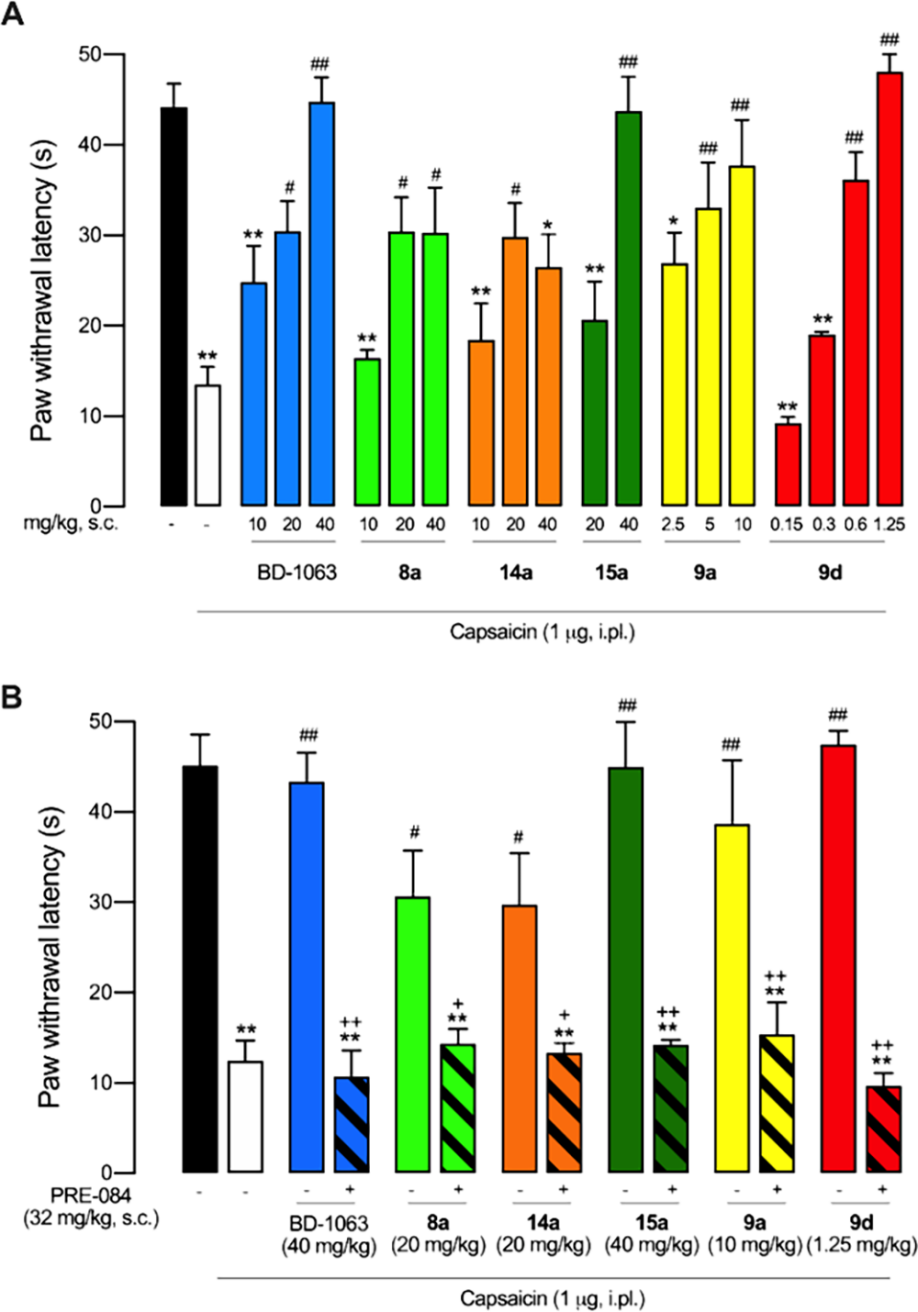
Reduction of capsaicin-induced mechanical hypersensitivity by the systemic administration of the experimental compounds and BD-1063 as standard S1R antagonist in mice, and contribution of S1R to their effects. (A) Dose dependency of the antinociceptive effects of the subcutaneous (s.c.) administration of BD-1063, **8a**, **9a**, **9d**, **14a**, and **15a**. (B) Effects of the compounds tested alone and associated with the S1R agonist PRE-084. Values are the mean ± SEM obtained from 6–9 animals per group: *p < 0.05, ^**^p < 0.01 vs non-sensitized animals treated with the solvent of the drugs (black bar); ^#^*p* < 0.05, ^##^*p* < 0.01 vs capsaicin-injected mice treated with the solvent of the drugs (white bar); ^+^*p* < 0.05, ^++^*p* < 0.01 selected doses of each compound associated with PRE-084 or its solvent (one-way ANOVA followed by Student–Newman–Keuls test).

We tested the effect of several of our compounds in capsaicin-induced mechanical hypersensitivity (allodynia) in mice (Figure 5). The increase in pain sensitivity in the area surrounding capsaicin injection results from central sensitization, and this process plays a pivotal role in chronic pain development and maintenance.^29^ Capsaicin-induced mechanical hypersensitivity has been used to study drug effects in central sensitization in both humans and rodents.^30,31^ In particular, this behavioral model has been previously employed to evaluate the S1R functional profile of new compounds (including clinical candidates) since compounds that act as an antagonist at S1R can reduce sensory hypersensitivity, whereas compounds that act as agonists at S1R reverse the effects of the former.^30,32,33^

Non-sensitized mice showed a response latency to the mechanical stimulation of 44.19 ± 2.59 s. The response latency markedly decreased in mice intraplantarly treated with capsaicin up to 13.51 ± 1.93 s, denoting the development of tactile allodynia (Figure 5A). BD-1063 (10–40 mg/kg, s.c.), used as a control standard S1R antagonist, induced a dose-dependent and full reversal of capsaicin-induced allodynia, as previously described.^30,33^ Compounds **8a** and **14a** (10–40 mg/kg, s.c.) also induced dose-dependent antiallodynic effects. However, the extent of their effects was limited in comparison to BD-1063, as they were unable to fully reverse capsaicin-induced hypersensitivity at 40 mg/kg, reaching latency values of just 30.28 ± 5.02 s for **8a** and 26.55 ± 3.55 s for **14a** (Figure 5A). The s.c. administration of **15a** (20–40 mg/kg) induced a dose-dependent and full antiallodynic effect, similar to the effect of the standard BD-1063. Finally, we tested two compounds that outperformed BD-1063 in terms of potency. The administration of **9a** (2.5–10 mg/kg) induced dose-dependent and full reversal of capsaicin-induced allodynia, yielding significant antiallodynic effects from the dose of 5 mg/kg, and **9d** showed not only dose-dependent and full reversal of capsaicin-induced allodynia (similar to the last two compounds described above) but exhibited an extreme potency for this effect, reaching maximum antiallodynia at a dose as low as 0.6–1.25 mg/kg (Figure 5A).

**Figure 6:**
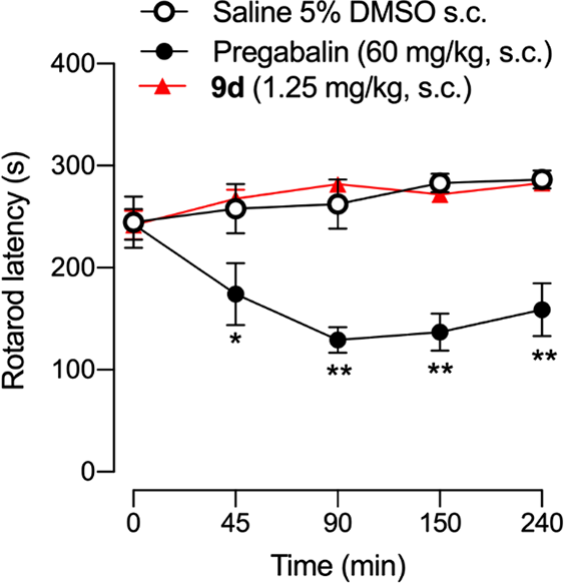
Effect of **9d** and pregabalin on motor coordination. The latency to fall-down from the rotarod was recorded in each mouse immediately before (time 0) and several times after the subcutaneous (s.c.) administration of **9d** (1.25 mg/kg), pregabalin (60 mg/kg), or their solvent (DMSO 5% in saline). Values are the mean ± SEM from 6–7 animals. Significant differences between the values at time 0 and after drug administration: ^**^p < 0.01 (2-way repeated measures ANOVA followed by Student–Newman–Keuls test).

We then tested the *in vivo* effects of the association of these compounds with PRE-084 (32 mg/kg, s.c.), a prototypic S1R agonist. We selected drug doses that induced the maximum antiallodynic effect of each compound (*i.e.* BD-1063 40 mg/kg, **8a** 20 mg/kg, **14a** 20 mg/kg, **15a** 40 mg/kg, **9a** 10 mg/kg, and **9d** 1.25 mg/kg). The administration of PRE-084 fully reversed the antiallodynic effect of BD-1063 (Figure 5B), as previously reported.^30,33^ Importantly, PRE-084 administration was also able to fully reverse the effect of all other experimental compounds. These results indicate that S1R receptor antagonism is essential for the effect of all these compounds on mechanical hypersensitivity (Figure 5B). It is worth mentioning that among the S1R compounds tested *in vivo*, **9d** was the most potent and showed a high affinity not only for S1R but also for S2R (see Tables 1 and 2). As it has been recently shown that S2R modulators can also induce antinociceptive effects,^34^ the participation of S2R on the antiallodynic effects induced by **9d** cannot be ruled out. We then tested the effects of **9d** on motor coordination (Figure 6). Assessment of drug-induced motor impairment is relevant for the interpretation of the results from tests for nociception since pharmacological treatment affecting motor functioning might attenuate nociceptive responses inducing false analgesic-like effects.^2^

The rotarod test is the most standard test to assess motor function and coordination in rodents. It consists of a rotating rod where the animal is placed, and the latency to fall is measured. Drugs that negatively affect motor coordination decrease the latency to fall.^2^ We treated animals with **9d** at a dose able to fully reverse mechanical hypersensitivity (1.25 mg/kg) and tested them on rotarod performance.

As shown in Figure 6, animals treated with **9d** showed no change in the latency to fall from the rotating drum in comparison to the baseline value (time 0) or to solvent-treated mice, at any time point tested during the 4 h evaluation period.

Hence, the results found on capsaicin-induced mechanical hypersensitivity of **9d** cannot be attributed to motor impairment. However, the administration of pregabalin, used as a positive control of a drug known to induce motor deficits,^35^ markedly reduced rotarod latencies (Figure 6). Therefore, the lack of effect of **9d** on the rotarod test was not due to any methodological pitfall.

### S1R Functional Assay for Compound **9d**

**Figure 7:**
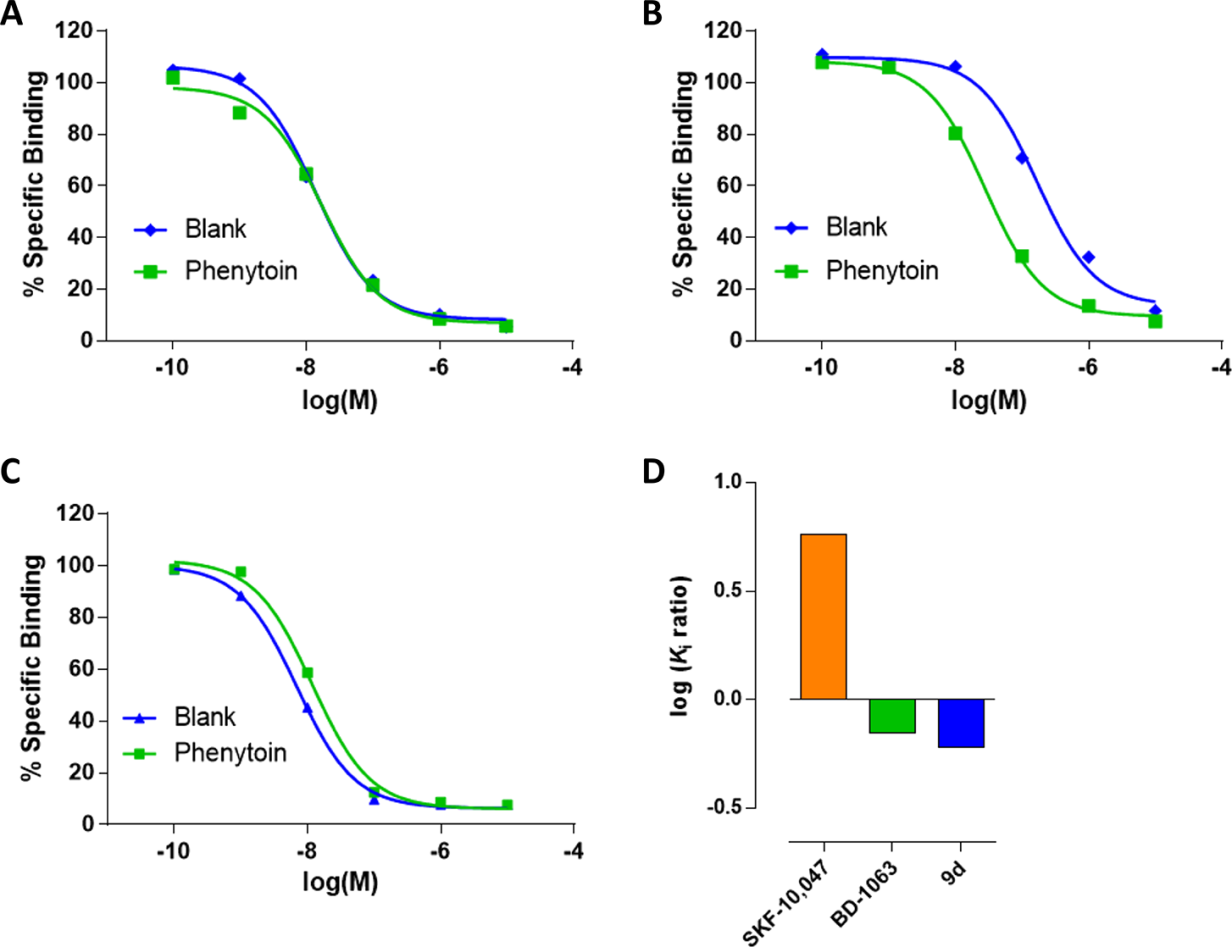
Radioligand displacement of (A) BD-1063, (B) SKF-10,047, and (C) compound **9d** in the presence (green) or absence (blue) of phenytoin. The ratio of log *K*_i_ values with or without phenytoin (D) in the S1R radiolabeled binding assays.

Compound **9d** was then subjected to an *in vitro* phenytoin assay for S1R functional profile determination. Phenytoin is a low-potent allosteric modulator for the S1R, differentially modulating the affinity of S1R ligands based on their agonist or antagonist functionality.^36^ Phenytoin improves the binding affinity of S1R agonists, while it has no effects or slightly decreases the binding affinity for S1R antagonists. The functionality of compound **9d** on S1R was determined by a radioligand binding assay using rat liver in the presence of phenytoin, together with the known S1R agonist SKF-10,047 and antagonist BD-1063 (Figure 7).

Both **9d** and BD-1063 exhibited a very small shift to lower receptor binding affinity with ratios of *K*_i_ without phenytoin/with phenytoin of 0.6 and 0.7, respectively. On the contrary, SKF-10,047 showed a ratio of 5.8 in the presence of phenytoin. These observations indicate that compound **9d** acts as an antagonist for the S1R, confirming the *in vivo* outcome deriving from the capsaicin-induced sensitization.

### Selectivity Profile for Compound **9d**

To assess the interactions of compound **9d** with other receptors, a target selectivity profile was investigated over supplementary receptors, including the opioid (MOR, DOR, KOR), serotoninergic (5HT2A, SERT), cannabinoid (CB1, CB2), and NMDA receptors. This panel includes targets validated for pain and others associated with undesirable side effects, to rule out off-target activities that could interfere with the analgesic response of the compounds. Notably, compound **9d** showed no significant affinity to any of these targets (inhibition <50% at 1 μM).

### Initial ADMET Profile

We profiled preliminary *in vitro* solubility and both chemical and metabolic stability assays of compound **9d** (Table 3). The water solubility was experimentally determined showing a value of 2.14 mM (0.71 mg/mL) at rt. The chemical stability was evaluated at 37 °C in an aqueous phosphate buffer (PBS) at pH 7.4 showing an optimal stability profile (*T*_1/2_ > 24 h). A similar profile was found when evaluated at 37 °C in human plasma (*T*_1/2_ > 24 h). Moreover, *in vitro* metabolic studies were performed using mouse and human liver microsomes. Following incubation at 0.1 μM with the liver microsomes for 1 h, reaction samples were analyzed by LC–MS/MS at different time points (0, 15, 30, 45, 60 min). Compound **9d** exhibited similar patterns in both mouse and human metabolism with intrinsic clearance (CL_int_) of 123.7 and 126.7 μL/min/mg protein in mouse and human liver microsomes and metabolic half-time (*T*_1/2_) around 1 h. Having a 50 > CL_int_ ≤ 150 μL/min/mg protein in both species, compound **9d** may be classified to have a moderate clearance value.^37^

**Table 3:** In Vitro Characterization of Compound **9d**

PK parameter		**9d**
solubility^a^		2.14
stability^b^	pH 7.4	>24
	plasma	>24
mouse liver microsomes	*T*_1/2_^c^	56
	CL_int_^d^	123.7
human liver microsomes	*T*_1/2_^c^	55
	CL_int_^d^	126.7

a mM.

b h.

c min.

d μL/min/mg protein.

Since the potassium ion channel coded by the human ether-a-go-go-related gene (hERG) inhibition was a recurrent issue in previous S1R programs^38,39^ and considering the chemical structures of **9d**, we sought to investigate its hERG blockade.^40^ Compound **9d** showed an IC_50_ 0.085 μM (below the standard compound verapamil, IC_50_ 0.27 μM), which means a 24-fold ratio versus its S1R affinity. Although it is thought that a 30-fold difference between the effective therapeutic plasma concentration and hERG IC_50_ may be sufficient to prevent the appearance of Torsades de Pointes associated with QT prolongation^41^ and **9d** exerts maximum antiallodynic effect in the mg/kg range, the usual IC_50_ cutoff is 10 μM. Hence, the high hERG inhibition of **9d** is an alert to be considered in the future optimization of the series.

## Conclusions

In this study, 19 analogs containing a central 2,7-diazaspiro[4.4]nonane moiety were synthesized and evaluated for SR affinity in radioligand binding assays. Iterative optimization was carried out according to structure–affinity relationships using the following steps: (i) design of new candidate ligands; (ii) *in vitro* radioligand binding assays; (iii) iterative compounds design based on affinity and selectivity; (iv) computational studies; and (v) synthesis of the new compounds for further pharmacological evaluation.

After this iterative process of optimization, compounds **8a**, **9a**, **9d**, and **14a** were selected for preliminary *in vitro* tests, revealing all to be well tolerated up to very high concentrations followed by testing compounds **8a**, **9a**, **9d**, **14a**, and **15a** for dose-dependent antiallodynic effects against capsaicin-induced pain in mice. Among these, compound **9d** exhibited affinity for both S1R and S2R, but not for other pain-related targets, and exerted promising dose-dependent antiallodynic effects against capsaicin-induced pain in mice, without displaying significant neurotoxicity or motor dysfunction in the rotarod test at effective doses for analgesia (1.25 mg/kg). The functional activity of compound **9d** was evaluated both through *in vivo* and *in vitro* experiments, showing that compound **9d** acts as an antagonist for the S1R. In fact, the antiallodynic effects of this compound are fully reversed by the S1R agonist PRE-084, indicating that S1R antagonism is essential for these effects. In conclusion, the present study provides new observations into the use of 2,7-diazaspiro[4.4]nonane scaffold for the development of SR ligands. Most notably, we report that the mixed S1R/S2R ligand **9d** has potent antiallodynic effects and a demonstrable dose able to fully reverse mechanical hypersensitivity of 1.25 mg/kg, making this compound one the most potent S1R/S2R ligand endowed with S1R antagonism reported so far. Further studies aimed at improving ADMET for this series of compounds will be the focus of future optimization campaigns.

## Experimental Section

### General Remarks

Reagent-grade chemicals were purchased from Merck (Darmstadt, Germany) and were used without further purification. All reactions involving air-sensitive reagents were performed in Ar or N_2_ in oven-dried glassware using the syringe-septum cap technique. Flash chromatography purification was performed on a Merck silica gel 60 (40–63 μm; 230–400 mesh) stationary phase. Nuclear magnetic resonance spectra (^1^H NMR recorded at 200 and 500 MHz) were obtained on VARIAN INOVA spectrometers using CDCl_3_. TMS was used as an internal standard. Chemical shifts (δ) are given in parts per million (ppm) and coupling constants (*J*) in Hertz (Hz). The following abbreviations are used to designate the multiplicities: s = singlet, d = doublet, t = triplet, q = quartet, quint = quintet, m = multiplet, br = broad. The purity of all tested compounds, whether synthesized or purchased, reached at least 95% as determined by microanalysis (C, H, N) that was performed on a Carlo Erba instrument model E1110; all the results agreed within ±0.4% of the theoretical values. Reactions were monitored by TLC performed on 250 μm silica gel Merck 60 F_254_-coated aluminum plates; the spots were visualized by UV light or iodine chamber. The nomenclatures were made with ChemDraw Professional version 16.0.0.82.

### General Procedure for Buchwald–Hartwig Amination (Procedure A)

A mixture of Pd_2_(dba)_3_ (3 mol %, 11 mg) and 2-dicyclohexylphosphino-2′,6′-dimethoxybiphenyl (8 mol %, 13 mg) in dry toluene (3 mL) was degassed under N_2_ for 30 min into an oven-dried sealed vial. Then, *para*-substituted iodobenzene (0.39 mmol), *tert*-butyl 2,7-diazaspiro[4.4]nonane-2-carboxylate (0.47 mmol, 106 mg), and *t*-BuONa (0.55 mmol, 62 mg) were sequentially added and the reaction was stirred. The reaction mixture was slowly brought to rt., quenched with H_2_O (5 mL), and extracted with EtOAc (2 × 10 mL). The organic layer was dried over Na_2_SO_4_ and concentrated under vacuum. The crude product was purified by flash chromatography on silica gel to afford the desired product. Products synthesized according to this procedure are **6a**–**d**.

### General Procedure for Amine Preparation (Procedure B)

To a solution of *tert*-butyl-2,7-diazaspiro[4.4]nonane-2-carboxylate (0.66 mmol, 150 mg) in ACN (5 mL), K_2_CO_3_ (1.32 mmol, 182 mg) and the halo-derivative (0.73 mmol) were sequentially added. The reaction was stirred for 3 h at rt, then quenched with H_2_O (5 mL), and extracted with EtOAc (2 × 10 mL). The organic layer was washed with brine (1 × 5 mL), dried over Na_2_SO_4_, filtered, and concentrated under vacuum. The residue was purified via silica gel chromatography to obtain the desired product. Products synthesized according to this procedure are **6e** and **12a**,**c**.

### General Procedure for Amine Preparation (Procedure C)

The Boc-protected amine (0.2 mmol) has been stirred with 30% TFA in CH_2_Cl_2_ (10 mL) at rt for 4 h followed by the removal of the solvents under vacuum. The residue was then dissolved in ACN (5 mL), and K_2_CO_3_ (0.3 mmol, 41 mg) and the appropriate bromide (0.2 mmol) were sequentially added. The reaction has been stirred under reflux on, quenched with H_2_O (5 mL), and extracted with EtOAc. The collected organic phases have been washed with brine (1 × 5 mL), dried over Na_2_SO_4_, and evaporated to dryness. Products synthesized according to this procedure are **8a**,**b**, **9a**,**d 13a**,**b**, and **14a**,**b**.

### General Procedure for Amine Preparation (Procedure D)

To a solution of *tert*-butyl-2,7-diazaspiro[4.4]nonane-2-carboxylate (0.44 mmol, 100 mg) in dry CH_2_Cl_2_ (5 mL), TEA (0.66 mmol, 92.41 μL) and acyl chloride (0.88 mmol) have been added dropwise at 0 °C. The reaction has been stirred for 1 h at rt, then quenched with cold H_2_O (5 mL), diluted with CH_2_Cl_2_ (10 mL), and washed with 5% NH_4_Cl (1 × 5 mL), and a saturated solution of NaHCO_3_ (1 × 5 mL). The combined organic extracts were dried over anhydrous Na_2_SO_4_ and concentrated under vacuum. The residue has been purified by flash chromatography. Products synthesized according to this procedure are **10a**,**b**.

### General Procedure for Amine Preparation (Procedure E)

A mixture of Boc-protected amine (0.30 mmol) with 30% TFA in CH_2_Cl_2_ (10 mL) has been stirred at rt for 4 h, followed by the removal of the solvents under vacuum. The residue was dissolved in fresh dry CH_2_Cl_2_ (5 mL), and TEA (0.53 mmol, 75 μL) and acyl chloride (0.36 mmol) have been dropwise added at 0 °C. The reaction has been stirred at rtfor 2 h and then quenched with H_2_O (5 mL), diluted with CH_2_Cl_2_ (10 mL), washed with 5% NH_4_Cl (1 × 5 mL), and then with a saturated solution of NaHCO_3_ (1 × 5 mL). The combined organic extracts were dried over anhydrous Na_2_SO_4_ and concentrated under vacuum. The residue has been purified by flash chromatography to obtain the desired product. Products synthesized according to this procedure are **7a**, **11a**, and **15c**–**e**.

### General Procedure for Amide Bond Reduction (Procedure F)

To a solution of amide (0.14 mmol) in THF (10 mL), LiAlH_4_ (4 M in THF, 0.82 mmol) was added dropwise at −10 °C under a N_2_ atmosphere. The resulting mixture was stirred for 2 h at the appropriate temperature. Then, the reaction was quenched with ice-cold H_2_O (1 mL) and 1 M NaOH (1 mL) at 0 °C, filtered through Celite, and washed with MeOH. The solution was concentrated under reduced pressure and dissolved in EtOAc. The organic phase was dried over anhydrous Na_2_SO_4_ and concentrated under vacuum. The residue has been purified by flash chromatography with 100% CH_2_Cl_2_ and then with 3% MeOH in CH_2_Cl_2_ + 1% NH_4_OH. Products synthesized according to this procedure are **7b**, **11b**, and **16a**–**c**.

### General Procedure for Coupling Reaction (Procedure G)

A mixture of Boc-protected amine (0.31 mmol) with 30% TFA in CH_2_Cl_2_ (10 mL) has been stirred at rt for 4 h followed by the removal of the solvents under vacuum. Meanwhile, to a solution of carboxylic acid (0.40 mmol) in ACN (5 mL), EDC (0.46 mmol, 72 mg), and HOBT (0.46 mmol, 63 mg) were added at 0 °C. After 20 min, the previously prepared amine in ACN (2 mL) and DIPEA (0.93 mmol, 162 μL) were added at 0 °C. The reaction has been stirred at rt for 24 h. After the reaction was complete, it was diluted with EtOAc (5 mL), washed with H_2_O (1 × 5 mL), saturated solution of NaHCO_3_ (1 × 5 mL), and brine (1 × 5 mL). The combined organic extracts were dried over anhydrous Na_2_SO_4_ and concentrated under vacuum. The residue has been purified by flash chromatography. Products synthesized according to this procedure are **15a**,**b**.

### General Procedure for Oxalate Preparation (Procedure H)

The pure compound was dissolved in diethyl ether and a solution of oxalic acid in diethyl ether was added dropwise to obtain the desired product as oxalic acid salt. All the final compounds have been prepared as oxalic acid salts.

#### *tert*-Butyl-7-phenyl-2,7-diazaspiro[4.4]nonane-2-carboxylate (**6a**)

The compound has been prepared using iodobenzene (0.39 mmol, 43.6 μL) following Procedure A. The reaction was heated to 100 °C. The crude product was purified by flash chromatography using Hex/EtOAc (95:5). Yield: 82%, brown oil. ^1^H NMR (200 MHz, CDCl_3_) δ 7.18–7.31 (m, 2H), 6.70 (t, *J* = 7.2 Hz, 1H), 6.55 (d, *J* = 8.0 Hz, 2H), 3.18–3.58 (m, 8H), 1.76–2.09 (m, 4H), 1.47 (s, 9H).

#### tert-Butyl-7-(4-nitrophenyl)-2,7-diazaspiro[4.4]nonane-2-carboxylate (**6b**)

The compound has been prepared using 1-iodo-4-nitrobenzene (0.39 mmol, 97 mg) following Procedure A. The reaction was left stirring at rt. The crude product was purified by flash chromatography using EtOAc/Hex (70:30). Yield: 88%, orange oil. ^1^H NMR (200 MHz, CDCl_3_) δ 8.11 (d, *J* = 9.4 Hz, 2H), 6.45 (d, *J* = 9.0 Hz, 2H), 3.21–3.67 (m, 8H), 1.75–2.19 (m, 4H), 1.46 (s, 9H).

#### tert-Butyl-7-(4-methoxyphenyl)-2,7-diazaspiro [4.4]nonane-2-carboxylate (**6c**)

Following Procedure A, the compound was prepared using 4-iodoanisole (0.39 mmol, 91 mg). The reaction mixture was heated to 100 °C. The crude product was purified by flash chromatography using EtOAc/Hex (70:30). Yield: 51%, orange oil. ^1^H NMR (200 MHz, CDCl_3_) δ 6.86 (d, *J* = 8.6 Hz, 2H), 6.51 (d, *J* = 8.6 Hz, 2H), 3.77 (s, 3H), 3.10–3.62 (m, 8H), 1.74–2.13 (m, 4H), 1.47 (s, 9H).

#### tert-Butyl-7 (4-acetylphenyl) -2,7-diazaspiro[4.4]nonane-2-carboxylate (**6d**)

Following Procedure A, the compound was prepared using 4-iodoacetophenone (0.39 mmol, 96 mg). The reaction was left stirring at rt. The crude product was purified by flash chromatography using EtOAc/Hex (70:30). Yield: 76%, orange oil. ^1^H NMR (200 MHz, CDCl_3_) δ 7.85 (δ, *J* = 8.2 Hz, 2H), 6.48 (δ, *J* = 8.2 Hz, 2H), 3.19–3.61 (m, 8H), 2.49 (s, 3H), 1.79–2.19 (m, 4H), 1.44 (s, 9H).

#### tert-Butyl-7-(4-cyanophenyl)-2,7-diazaspiro[4.4]nonane-2-carboxylate (**6e**)

The compound has been prepared using 4-chlorobenzonitrile (0.73 mmol, 100 mg) and DMSO as solvent following Procedure B. The reaction mixture was heated to 120 °C. The crude product was purified by flash chromatography using Hex/EtOAc (90:10). Yield: 56%, white solid. ^1^H NMR (200 MHz, CDCl_3_) δ 7.46 (d, *J* = 8.6 Hz, 2H), 6.49 (d, *J* = 8.2 Hz, 2H), 3.14–3.58 (m, 8H), 1.81–2.18 (m, 4H), 1.34–1.56 (m, 9H).

#### Phenyl(7-phenyl-2,7-diazaspiro[4.4]nonan-2-yl)methanone (**7a**)

The compound has been prepared using **6a** (0.30 mmol, 91 mg) and benzoyl chloride (0.36 mmol, 41.8 μL) following Procedure E. The residue has been purified by flash chromatography with Hex/EtOAc (80:20). Yield: 70%, yellow oil. ^1^H NMR (200 MHz, CDCl_3_) δ 8.05–8.15 (m, 3H), 7.39–7.52 (m, 5H), 7.05–7.23 (m, 2H), 2.84–3.97 (m, 8H), 1.70–2.16 (m, 2H), 0.94–1.45 (m, 2H).

#### 2-Benzyl-7-phenyl-2,7-diazaspiro[4.4]nonane (**7b**, **AD214**)

The compound has been prepared using **7a** (0.14 mmol, 43 mg) following Procedure F. The reaction was allowed to warm to rt. The residue has been purified by flash chromatography with Hex/EtOAc (60:40) followed by conversion into oxalic acid salt according to Procedure H. Yield: 20%, white solid. ^1^H NMR (200 MHz, CDCl_3_ – free base) δ 7.08–7.40 (m, 7H), 6.55–6.68 (m, 1H), 6.48 (d, *J* = 8.3 Hz, 2H), 3.59 (d, *J* = 1.5 Hz, 2H), 3.23 (quin, *J* = 8.9 Hz, 4H), 2.35–2.76 (m, 4H), 1.68–2.09 (m, 4H). ^13^C NMR (200 MHz, CDCl_3_ – free base) δ 147.7, 138.8, 129.1, 128.7, 128.3, 126.9, 115.6, 111.3, 64.6, 60.5, 59.6, 53.9, 48.0, 47.0, 38.0, 36.1. Anal. calcd for C_20_H_24_N_2_·H_2_C_2_O_4_: C, 69.09; H, 6.85; N, 7.32; found: C, 69.22; H, 6.88; N, 7.27.

#### 2-Phenethyl-7-phenyl-2,7-diazaspiro[4.4]nonane (**8a**, **AD174**)

The compound has been prepared using **6a** (0.2 mmol, 60 mg) and (2-bromoethyl)benzene (0.2 mmol, 27.3 μL) following Procedure C. The crude product was purified by flash chromatography using 1% MeOH in CH_2_Cl_2_. After purification, the pure product was converted into oxalate salt following Procedure H. Yield: 20%, yellow solid. ^1^H NMR (200 MHz, CDCl_3_ – free base) δ 7.16–7.53 (m, 7H), 6.71 (t, *J* = 7.4 Hz, 4H), 6.58 (d, *J* = 7.8 Hz, 3H), 3.22–3.48 (m, 4H), 2.57–2.96 (m, 8H), 1.80–2.19 (m, 4H). ^13^C NMR (200 MHz, CDCl_3_ – free base) δ 147.7, 140.1, 129.2, 128.7, 128.3, 126.1, 115.4, 111.4, 64.8, 59.6, 58.4, 54.2, 48.0, 47.1, 37.9, 36.0, 35.4. Anal. calcd for C_21_H_26_N_2_·C_2_H_2_O_4_: C, 69.68; H, 7.12; N, 7.07; found: C, 69.42; H, 7.11; N, 7.09.

#### 2-Phenyl-7-(3-phenylpropyl)-2,7-diazaspiro[4.4]nonane (**8b**, **AD157**)

The compound has been prepared using **6a** (0.2 mmol, 60 mg) and (3-bromopropyl)benzene (0.2 mmol, 30.4 μL) following Procedure C. The crude product was purified by flash chromatography using 5% MeOH in CH_2_Cl_2_. After purification, the pure product was converted into oxalate salt following Procedure H. Yield: 25%, brown solid. ^1^H NMR (200 MHz, CDCl_3_ – free base) δ 7.10–7.39 (m, 7H), 6.63–6.75 (m, 1H), 6.53 (d, *J* = 8.3 Hz, 2H), 3.21–3.43 (m, 5H), 2.97–3.17 (m, 3H), 2.61–2.85 (m, 5H), 1.91–2.21 (m, 5H). ^13^C NMR (200 MHz, CDCl_3_ – free base) δ 147.7, 138.8, 129.1, 128.7, 128.3, 126.9, 115.3, 111.3, 64.6, 60.5, 59.6, 53.9, 48.0, 47.0, 38.0, 36.1, 29.7. Anal. calcd for C_22_H_28_N_2_·H_2_C_2_O_4_: C, 70.22; H, 7.37; N, 6.82; found: C, 70.56; H, 7.38; N, 6.86.

#### 2-(4-Nitrophenyl)-7-phenethyl-2,7-diazaspiro[4.4]nonane (**9a**, **AD242**)

The compound has been prepared using **6b** (0.2 mmol, 69 mg) and (2-bromoethyl)benzene (0.2 mmol, 27.3 μL) following Procedure C. The reaction has been stirred at rt for 48 h and the crude product was purified by flash chromatography using Hex/EtOAc (70:30 to 30:70). After purification, the pure product was converted into oxalate salt according to Procedure H. Yield: 55%, yellow solid. ^1^H NMR (500 MHz, CDCl_3_ – free base) δ 8.00–8.09 (m, 2H), 7.18–7.24 (m, 2H), 7.10–7.16 (m, 3H), 6.35–6.41 (m, 2H), 3.33–3.45 (m, 3H), 3.24 (d, *J* = 9.8 Hz, 1H), 2.70–2.78 (m, 3H), 2.56–2.67 (m, 4H), 2.46 (d, *J* = 8.8 Hz, 1H), 1.93–2.07 (m, 2H), 1.65–1.87 (m, 2H). ^13^C NMR (200 MHz, CDCl_3_ – free base) δ 151.8, 140.1, 136.7, 128.5, 126.3, 126.1, 110.3, 64.4, 59.8, 58.1, 53.8, 48.0, 47.5, 37.4, 35.5. Anal. calcd for C_21_H_25_N_3_O_2_·H_2_C_2_O_2_: C, 62.57; H, 6.16; N, 9.52; found: C, 62.95; H, 6.19; N, 9.49.

#### 2-(4-Methoxyphenyl)-7-phenethyl-2,7-diazaspiro[4.4]nonane (**9b**, **AD239**)

The compound has been prepared using **6c** (0.2 mmol, 66 mg) and 2-bromoethylbenzene (0.2 mmol, 27.3 μL) following Procedure C. The reaction has been stirred at 50 °C for 5 h and the crude product was purified by flash chromatography using 2% MeOH in CH_2_Cl_2_. After purification, the product was converted into oxalate salt according to Procedure H. Yield: 6%, dark solid. ^1^H NMR (500 MHz, CDCl_3_ – free base) δ 7.25–7.32 (m, 2H), 7.17–7.23 (m, 3H), 6.85 (d, *J* = 8.8 Hz, 2H), 6.50 (d, *J* = 8.8 Hz, 2H), 3.75 (s, 3H), 3.26–3.35 (m, 3H), 3.19 (d, *J* = 8.8 Hz, 1H), 2.70–2.88 (m, 7H), 2.62 (d, *J* = 8.8 Hz, 1H), 1.82–2.09 (m, 4H); ^13^C NMR (200 MHz, CDCl_3_ – free base) δ 150.8, 143.0, 139.9, 128.5, 115.0, 112.1, 65.0, 56.0, 54.1, 48.1, 36.1. Anal. calcd for C_22_H_28_N_2_O·H_2_C_2_O_2_: C, 67.59; H, 7.09; N, 6.57; found: C, 67.89; H, 7.11; N, 6.54.

#### 1-(4-(7-Phenethyl-2,7-diazaspiro[4.4]nonan-2-yl)phenyl)ethan-1-one (**9c**, **AD245**)

The compound has been prepared using **6d** (0.2 mmol, 69 mg) and (2-bromoethyl)benzene (0.2 mmol, 27.3 μL) following Procedure C. The reaction has been stirred at 50 °C for 5 h, and the crude product was purified by flash chromatography using Hex/EtOAc (90:10 to 50:50) followed by conversion into oxalic acid salt. Yield: 13%, yellow solid. ^1^H NMR (500 MHz, CDCl_3_ – free base) δ 7.87 (d, *J* = 8.8 Hz, 2H), 7.24–7.32 (m, 2H), 7.17–7.23 (m, 3H), 6.50 (d, *J* = 8.8 Hz, 2H), 3.37–3.48 (m, 3H), 3.29 (d, *J* = 9.3 Hz, 1H), 2.77–2.85 (m, 3H), 2.66–2.76 (m, 4H), 2.56 (d, *J* = 9.3 Hz, 1H), 2.50 (s, 3H), 1.98–2.11 (m, 2H), 1.83–1.96 (m, 2H); ^13^C NMR (200 MHz, CDCl_3_ – free base) δ 196.3, 150.9, 140.1, 130.4, 128.6, 128.3, 125.0, 110.5, 64.5, 59.5, 58.2, 53.9, 48.0, 47.1, 37.6, 35.7, 35.3, 26.0, 25.9. Anal. calcd for C_23_H_28_N_2_O·H_2_C_2_O_4_: C, 62.97; H, 7.23; N, 7.73; found: C, 63.31; H, 7.28; N, 7.70.

#### 4-(7-Phenethyl-2,7-diazaspiro[4.4]nonan-2-yl)benzonitrile (**9d**, **AD258**)

The compound has been prepared using **6e** (0.2 mmol, 65 mg) and (2-bromoethyl)benzene (0.2 mmol, 27.3 μL) following Procedure C. The crude product was purified by flash chromatography using 2% MeOH in CH_2_Cl_2_ and then converted into oxalate salt following Procedure H. Yield: 10%, clear solid. ^1^H NMR (500 MHz, CDCl_3_ – free base) δ 7.45 (d, *J* = 9.3 Hz, 2H), 7.25–7.31 (m, 2H), 7.17–7.24 (m, 3H), 6.48 (d, *J* = 8.8 Hz, 2H), 3.33–3.44 (m, 3H), 3.25 (d, *J* = 9.3 Hz, 1H), 2.76–2.84 (m, 3H), 2.63–2.74 (m, 4H), 2.49–2.57 (m, 1H), 1.95–2.11 (m, 2H), 1.81–1.94 (m, 2H); ^13^C NMR (200 MHz, CDCl_3_ – free base) δ 149.9, 140.1, 133.6, 128.2, 126.1, 120.9, 111.5, 96.8, 64.5, 59.5, 58.2, 53.9, 48.0, 47.1, 37.5, 35.6. Anal. calcd for C_22_H_25_N_3_·H_2_C_2_O_4_: C, 68.39; H, 6.46; N, 9.97; found: C, 68.91; H, 6.49; N, 9.91.

#### tert-Butyl-7-benzoyl-2,7-diazaspiro[4.4]nonane-2-carboxylate (**10a**)

The compound has been prepared using benzoyl chloride (0.88 mmol, 102.1 μL) following Procedure D. The residue has been purified by flash chromatography with EtOAc/Hex (90:10 to 70:30). Yield: 98%, colorless oil. ^1^H NMR (200 MHz, CDCl_3_) δ 7.35–7.58 (m, 5H), 3.14–3.85 (m, 8H), 1.72–2.02 (m, 4H), 1.45 (s, 9H).

#### tert-Butyl-7-(phenylacetyl)-2,7-diazaspiro[4.4]nonane-2-carboxylate (**10b**)

The compound has been prepared using phenylacetyl chloride (0.88 mmol, 116.4 μL) following Procedure D. The residue has been purified by flash chromatography with EtOAc/Hex (70:30 to 90:10). Yield: 60%, colorless oil. ^1^H NMR (200 MHz, CDCl_3_) δ 7.20–7.41 (m, 5H), 3.14–3.71 (m, 10H), 1.61–1.98 (m, 4H), 1.45 (s, 9H).

#### (2,7-Diazaspiro[4.4]nonane-2,7-diyl)bis(phenylmethanone) (**11a**)

The compound has been prepared using **10a** (0.30 mmol, 99 mg) and benzoyl chloride (0.36 mmol, 41.8 μL) following Procedure E. The residue has been purified by flash chromatography with 100% EtOAc. Yield: 76%, colorless oil. ^1^H NMR (200 MHz, CDCl_3_) δ (200 MHz, CDCl_3_) δ 7.31–7.66 (m, 10H), 3.20–3.87 (m, 8H), 1.73–2.18 (m, 4H).

#### 2,7-Dibenzyl-2,7-diazaspiro[4.4]nonane (**11b**, **AD206**)

The compound has been prepared using **11a** (0.14 mmol, 47 mg) following Procedure F. The reaction was warmed to 65 °C. The residue has been purified by flash chromatography with 100% CH_2_Cl_2_ and then with 3% MeOH in CH_2_Cl_2_. After purification, the product was converted into oxalate salt following Procedure H. Yield: 83%, white solid. ^1^H NMR (200 MHz, CDCl_3_ – free base) δ 7.15–7.36 (m, 10H), 3.59 (s, 4H), 2.36–2.68 (m, 8H), 1.69–2.00 (m, 4H). ^13^C NMR (200 MHz, CDCl_3_ – free base) δ 139.1, 128.8, 128.1, 126.8, 67.3, 62.6, 60.5, 53.8, 47.5, 39.3. Anal. calcd for C_21_H_26_N_2_·H_2_C_2_O_4_: C, 69.68; H, 7.12; N, 7.07; found: C 69.98; H, 7.17; N, 7.04.

#### tert-Butyl-7-benzyl-2,7-diazaspiro[4.4]nonane-2-carboxylate (**12a**)

Following Procedure B, the compound was prepared using benzyl bromide (0.73 mmol, 86.8 μL). The crude product was purified by flash chromatography using Hex/EtOAc (70:30). Yield: 60%, clear oil. ^1^H NMR (200 MHz, CDCl_3_) δ 7.15–7.35 (m, 5H), 3.57 (s, 2H), 3.07–3.42 (m, 4H), 2.26–2.75 (m, 4H), 1.62–1.95 (m, 4H), 1.34–1.48 (m, 9H).

#### tert-Butyl-7-phenethyl-2,7-diazaspiro[4.4]nonane-2-carboxylate (**12b**)

Following Procedure B, the compound was prepared using (2-bromoethyl)benzene (0.73 mmol, 99.7 μL). The reaction has been left to stir at 50 °C. The crude product was purified by flash chromatography with 5% MeOH in EtOAc. Yield: 50%, yellow oil. ^1^H NMR (200 MHz, CDCl_3_) δ 7.12–7.30 (m, 5H), 3.13–3.52 (m, 5H), 2.57–2.87 (m, 7H), 1.68–1.95 (m, 4H), 1.46 (s, 9H).

#### tert-Butyl-7-(3-phenylpropyl)-2,7-diazaspiro[4.4]nonane-2-carboxylate (**12c**)

Following Procedure B, the compound has been prepared using (3-bromopropyl)benzene (0.73 mmol, 111 μL). The reaction has been left to stir at 50 °C. The crude product was purified by flash chromatography with 2% MeOH in EtOAc. Yield: 91%, yellow oil. ^1^H NMR (200 MHz, CDCl_3_) δ 7.12–7.44 (m, 5H), 3.21–3.63 (m, 4H), 2.41–2.98 (m, 8H), 1.75–2.10 (m, 6H), 1.53 (s, 9H).

#### 2-Benzyl-7-phenethyl-2,7-diazaspiro[4.4]nonane (**13a**, **AD145**)

The compound has been prepared using **12a** (0.2 mmol, 63 mg) and (2-bromoethyl)benzene (0.2 mmol, 27.3 μL) following Procedure C. The crude product was purified by flash chromatography using 4% MeOH in CH_2_Cl_2_. After purification, the pure product was converted into oxalate salt following Procedure H. Yield: 25%, orange solid. ^1^H NMR (200 MHz, CDCl_3_ – free base) δ 7.14–7.48 (m, 10H), 3.76 (s, 2H), 3.10 (br. s., 8H), 2.55–2.93 (m, 4H–, 1.86–2.20 (m, 4H). ^13^C NMR (200 MHz, CDCl_3_ – free base) δ 137.2, 136.3, 129.2, 128.6, 127.8, 127.0, 64.9, 64.5, 59.5, 57.7, 53.7, 52.8, 47.7, 37.1, 36.8, 32.9. Anal. calcd for C_22_H_28_N_2_·H_2_C_2_O_4_: C, 70.22; H, 7.37; N, 6.82; found: C, 70.51; H, 7.35; 6.81.

#### 2-Benzyl-7-(3-phenylpropyl)-2,7-diazaspiro[4.4]nonane (**13b**, **AD193**)

The compound has been prepared using **12a** (0.2 mmol, 63 mg) and (3-bromopropyl)benzene (0.2 mmol, 30.4 μL) following Procedure C. The crude product was purified by flash chromatography using 4% MeOH in CH_2_Cl_2_. After purification, the product was converted into oxalate salt following Procedure H. Yield: 37%, yellow solid. ^1^H NMR (200 MHz, CDCl_3_ – free base) δ 7.04–7.44 (m, 10H), 3.58 (s, 2H), 2.32–2.71 (m, 12H), 1.81 (d, *J* = 6.5 Hz, 6H). ^13^C NMR (200 MHz, CDCl_3_ – free base) δ 140.9, 139.4, 129.5, 129.2, 129.0, 128.9, 128.0, 126.9, 60.4, 56.5, 56.1, 54.3, 53.5, 48.3, 38.1, 37.7, 33.7. Anal. calcd for C_23_H_30_N_2_·H_2_C_2_O_4_: C, 70.73; H, 7.60; N, 6.60; found: C, 70.69; H, 7.58; N, 6.58.

#### 2,7-Diphenethyl-2,7-diazaspiro[4.4]nonane (**14a**, **AD181**)

The compound has been prepared using **12b** (0.2 mmol, 66 mg) and (2-bromoethyl)benzene (0.2 mmol, 27.3 μL) following Procedure C. The crude product was purified by flash chromatography using 3% MeOH in CH_2_Cl_2_ followed by conversion into oxalic acid salt according to Procedure H. Yield: 42%, white solid. ^1^H NMR (200 MHz, CDCl_3_ – free base) δ 7.14–7.43 (m, 10H), 2.57–2.99 (m, 16H), 1.83–2.12 (m, 4H). ^13^C NMR (200 MHz, CDCl_3_ – free base) δ 139.9, 128.4, 125.8, 67.2, 67.0, 66.8, 58.3, 53.9, 47.6, 38.5, 35.1. Anal. calcd for C_23_H_30_N_2_·H_2_C_2_O_4_: C, 70.73; H, 7.60; N, 6.60; found: 70.90; H, 7.55; N, 6.58.

#### 2-Phenethyl-7-(3-phenylpropyl)-2,7-diazaspiro[4.4]nonane (**14b**, **AD182**)

The compound has been prepared using **12b** (0.2 mmol, 66 mg) and (3-bromopropyl)benzene (0.2 mmol, 30.4 μL) following Procedure C. The crude product was purified by flash chromatography using 3% MeOH in CH_2_Cl_2_ followed by conversion into oxalate salt according to Procedure H. Yield: 39%, yellow solid. ^1^H NMR (200 MHz, CDCl_3_ – free base) δ 7.10–7.47 (m, 10H), 2.58–3.03 (m, 16H), 1.80–2.21 (m, 6H). ^13^C NMR (200 MHz, CDCl_3_ – free base) δ 141.0, 139.3, 128.6, 128.4, 128.3, 126.3, 126.0, 65.8, 57.9, 55.8, 53.6, 47.6, 37.6, 34.6, 33.3, 28.8. Anal. calcd for C_24_H_32_N_2_·H_2_C_2_O_4_: C, 71.21; H, 7.81; N, 6.39; found: C, 71.80; H, 7.82; N, 6.40.

#### 2-(3,4-Dichlorophenyl)-1-(7-phenethyl-2,7-diazaspiro[4.4]nonan-2-yl)ethan-1-one (**15a**, **AD220**)

The compound has been prepared using **12b** (0.31 mmol, 102 mg) and 3,4-dichlorophenylacetic acid (0.40 mmol, 82 mg) following Procedure G. The residue has been purified by flash chromatography with 5% MeOH in CH_2_Cl_2_ and then converted into oxalate salt following Procedure H. Yield: 40%, light yellow solid. ^1^H NMR (200 MHz, CDCl_3_ – free base) δ 6.89–7.39 (m, 8H), 3.31–3.69 (m, 8H), 2.78–3.26 (m, 6H), 1.77–2.20 (m, 4H); ^13^C NMR (200 MHz, CDCl_3_ – free base) δ 168.8, 162.8, 162.1, 135.9, 134.6, 134.4, 132.3, 132.2, 131.1, 130.9, 130.8, 130.3, 128.9, 128.7, 128.5, 127.2, 61.2, 60.9, 57.0, 56.2, 55.4, 53.1, 52.9, 48.3, 46.4, 45.6, 44.8, 40.5, 40.1, 36.3, 34.8, 33.9, 33.4, 31.9. Anal. calcd for C_23_H_26_Cl_2_N_2_O·H_2_C_2_O_4_: C, 59.18; H, 5.56; N, 5.52; found: C, 59.26; H, 5.59; N, 5.49.

#### 2-(3,4-Dimethoxyphenyl)-1-(7-phenethyl-2,7-diazaspiro[4,4]nonan-2-yl)ethan-1-one (**15b**, **AD226**)

The compound has been prepared using **12b** (0.31 mmol, 102 mg) and 3,4-dimethoxyphenylacetic acid (0.40 mmol, 78 mg) following Procedure G. The residue has been purified by flash chromatography with 100% EtOAc followed by conversion into oxalate salt according to Procedure H. Yield: 25%, white solid. ^1^H NMR (200 MHz, CDCl_3_ – free base) δ 7.13–7.44 (m, 5H), 6.75–6.96 (m, 3H), 3.79–3.97 (m, 6H), 3.21–3.70 (m, 6H), 2.30–3.01 (m, 6H), 1.71–2.02 (m, 6H); ^13^C NMR (200 MHz, CDCl_3_ – free base) δ 170.0, 148.9, 147.8, 128.6, 128.4, 127.2, 126.1, 121.0, 111.9, 111.0, 63.9, 58.7, 58.0, 55.8, 53.8, 48.5, 46.6, 45.3, 36.2, 35.5. Anal. calcd for C_25_H_32_N_2_O_3_·H_2_C_2_O_4_: C, 65.04; H, 6.87; N, 5.62; found: C, 65.57; H, 6.91; N, 5.57.

#### 3-Phenyl-1-(7-(3-phenylpropyl)-2,7-diazaspiro[4.4]nonan-2-yl)propan-1-one (**15c**)

The compound has been prepared using **12c** (0.27 mmol, 97 mg) and 3-phenylpropionyl chloride (0.32 mmol, 48 μL) following Procedure E. The residue has been purified by flash chromatography with 5% MeOH in CH_2_Cl_2_ followed by conversion into oxalate salt according to Procedure H. Yield: 90%, light yellow solid. ^1^H NMR (200 MHz, CDCl_3_ – free base) δ 7.04–7.42 (m, 10H), 3.09–3.77 (m, 5H), 2.89–3.06 (m, 2H), 2.24–2.81 (m, 10H), 1.58–1.99 (m, 5H). ^13^C NMR (200 MHz, CDCl_3_ – free base) δ 171.3, 139.7, 136.5, 128.6, 128.3, 126.4, 71.8, 71.7, 61.0, 55.8, 53.7, 50.6, 47.5, 37.0, 36.5, 36.1, 32.8, 27.2, 14.1. Anal. calcd for C_25_H_32_N_2_O·H_2_C_2_O_4_: C, 79.74; H, 8.57; N, 4.25; found: C, 69.96; H, 7.35; N, 5.98.

#### (7-Phenethyl-2,7-diazaspiro[4.4]nonan-2-yl)(phenyl)methanone (**15d**, **AD217**)

The compound has been prepared using **12b** (0.30 mmol, 99 mg) and benzoyl chloride (0.36 mmol, 41.8 μL) following Procedure E. The residue has been purified by flash chromatography with EtOAc/Hex (70:30 to 90:10). After purification, the product was converted into oxalate salt following Procedure H. Yield: 23%, white solid. ^1^H NMR (200 MHz, CDCl_3_ – free base) δ 7.09–7.60 (m, 10H), 3.29–3.84 (m, 4H), 2.65–3.13 (m, 6H), 2.34–2.61 (m, 4H), 1.73–2.19 (m, 4H). ^13^C NMR (200 MHz, CDCl_3_ – free base) δ 169.8, 138.8, 131.6, 129.6, 128.5, 128.2, 127.9, 127.0, 62.3, 60.3, 57.8, 53.2, 48.5, 46.7, 45.4, 36.2, 34.5. Anal. calcd for C_22_H_26_N_2_O·H_2_C_2_O_4_: C, 67.91; H, 6.65; N, 6.60; found: C, 68.08; H, 6.68; N, 6.55.

#### 1-(7-Phenethyl-2,7-diazaspiro[4.4]nonan-2-yl)-2-phenylethan-1-one (**15e**, **AD219**)

The compound has been prepared using **12b** (0.30 mmol, 99 mg) and phenylacetyl chloride (0.36 mmol, 47.6 μL) following Procedure E. The residue has been purified by flash chromatography with EtOAc/Hex (70:30 to 90:10) followed by conversion into oxalate salt according to Procedure H. Yield: 37%, light yellow solid. ^1^H NMR (200 MHz, CDCl_3_ – free base) δ 7.04–7.44 (m, 10H), 3.39–3.77 (m, 6H), 2.91–3.37 (m, 2H), 2.83 (d, *J* = 9.8 Hz, 4H), 2.52–2.78 (m, 2H), 1.68–2.15 (m, 4H). ^13^C NMR (200 MHz, CDCl_3_ – free base) δ 168.9, 139.9, 128.4, 125.8, 67.2, 67.0, 66.8, 58.3, 53.9, 47.6, 38.5, 35.1. Anal. calcd for C_23_H_28_N_2_O·H_2_C_2_O_4_: C, 68.47; H, 6.90; N, 6.39; found: C, 68.58; H, 6.95; N, 6.34.

#### 2-(3,4-Dichlorophenethyl)-7-phenethyl-2,7-diazaspiro[4.4]nonane (**16a**, **AD225**)

The compound has been prepared using **15a** (0.14 mmol, 58 mg) following Procedure F. The reaction was allowed to warm to rt. The residue has been purified by flash chromatography with 6% MeOH in CH_2_Cl_2_ and then converted into oxalate salt following Procedure H. Yield: 33%, white solid. ^1^H NMR (200 MHz, CDCl_3_ – free base) δ 7.02–7.48 (m, 8H), 2.50–3.08 (m, 16H), 1.82–2.16 (m, 4H); ^13^C NMR (200 MHz, CDCl_3_ – free base) δ 140.5, 139.8, 131.9, 129.9, 128.6, 128.4, 126.1, 67.2, 58.38, 57.6, 53.9, 47.5, 38.7, 35.1. Anal. calcd for C_23_H_28_Cl_2_N_2_·H_2_C_2_O_4_: C, 60.86; H, 6.13; N, 5.68; found: C, 61.35; H, 6.15; N, 5.65.

#### 2-(3,4-Dimethoxyphenethyl)-7-phenethyl-2,7-diazaspiro[4.4]nonane (**16b**, **AD234**)

The compound has been prepared using **15b** (0.14 mmol, 57 mg) following Procedure F. The reaction was allowed to warm to rt. The residue has been purified by flash chromatography with 100% CH_2_Cl_2_ and then with 5% MeOH in CH_2_Cl_2_. The product has been converted into oxalate salt following procedure H. Yield: 37%, white solid. ^1^H NMR (200 MHz, CDCl_3_ – free base) δ 7.14–7.38 (m, 5H), 6.67–6.87 (m, 3H), 3.87 (d, *J* = 3.1 Hz, 6H), 2.53–3.20 (m, 16H), 1.78–2.11 (m, 4H). ^13^C NMR (200 MHz, CDCl_3_ – free base) δ 148.9, 147.8, 128.6, 128.4, 127.2, 126.1, 121.0, 111.9, 111.0, 63.9, 58.7, 58.0, 55.8, 53.8, 48.5, 46.6, 45.3, 36.2, 35.5. Anal. calcd for C_25_H_34_N_2_O_2_·H_2_C_2_O_4_: C, 66.92; H, 7.49; N, 5.78; found: C, 67.18; H, 7.51; N, 5.79.

#### 2,7-Bis(3-phenylpropyl)-2,7-diazaspiro[4.4]nonane (**16c**, **AD267**)

The compound has been prepared using **15b** (0.19 mmol, 70 mg) following Procedure F. The reaction was allowed to warm to rt. The residue has been purified by flash chromatography with 100% CH_2_Cl_2_ and then with 5% MeOH in CH_2_Cl_2_. The product has been converted into oxalate salt following procedure H. Yield: 87%, white solid. ^1^H NMR (200 MHz, CDCl_3_ – free base) δ 7.08–7.40 (m, 10H), 2.50–2.70 (m, 10H), 2.37–2.49 (m, 6H), 1.63–1.96 (m, 8H). ^13^C NMR (200 MHz, CDCl_3_ – free base) δ 148.9, 147.8, 128.6, 128.4, 127.2, 126.1, 121.0, 111.9, 111.0, 63.9, 58.7, 58.0, 55.8, 53.8, 48.5, 46.6, 45.3, 36.2, 35.5. Anal. calcd for C_25_H_34_N_2_·H_2_C_2_O_4_: C, 71.65; H, 8.02; N, 6.19; found: C, 71.32; H, 8.04; N, 6.18.

### Radioligand Binding Assays

#### S1R and S2R Binding Affinity

S1R and S2R binding assays were performed using [^3^H] (+)-pentazocine (28.4 Ci/mmol) and [^3^H]1,3-di-*o*-tolylguanidine ([^3^H]DTG, 41.7 Ci/mmol), respectively (PerkinElmer, Belgium). All experiments were performed using ultrapure water obtained with a Millipore Milli-Q Reference Ultrapure Water Purification System. The Ultima Gold MV Scintillation cocktail was from PerkinElmer (Milan, Italy), Whatman GF 6 glass fiber filters from Merck (Darmstadt, Germany). For *in vitro* S1R radioligand binding assays, increasing concentrations of test compounds (from 0.1 nM to 10 μM), [^3^H] (+)-pentazocine (2 nM, *K*_d_ 2.9 nM), S1R Tris buffer (50 mM, pH 8) and the membrane preparation – liver homogenates from male Sprague Dawley rats – have been used in a final volume of 0.5 mL. Cold (+)-pentazocine (10 μM) was used to measure non-specific binding. The incubation was carried out for 120 min at 37 °C followed by fast filtration under reduced pressure using Millipore filter apparatus through Whatman GF/6 glass fiber filters presoaked in a 0.5% poly(ethyleneimine) water solution. Filters were rinsed three times with 3 mL of Tris buffer (50 mM, pH 8), dried, and incubated with a 3 mL scintillation cocktail in a 4 mL Kartell high-density polyethylene (HDPE) scintillation vial (Noviglio, Italy). The bound radioactivity has been determined by a liquid scintillation counter (Beckman LS 6500). *In vitro* S2R competition radioligand binding assays were performed using increasing concentrations of test compounds (from 0.1 nM to 10 μM), [^3^H]DTG (2 nM, *K*_d_ 17.9 nM), S2R Tris buffer (50 mM, pH 8), (+)-pentazocine (5 μM) as the S1R masking agent and liver homogenates from male Sprague Dawley rats, in a final volume of 0.5 mL. Measurement of non-specific binding was carried out using DTG (10 μM). The incubation was carried out for 120 min at 25 °C followed by fast filtration through Whatman GF 6 glass fiber filters presoaked in a 0.5% poly(ethylenimine) solution. Filters have been washed three times with 2 mL of Tris buffer (10 mM, pH 8), dried, and incubated with a 3 mL scintillation cocktail in 4 mL Kartell HDPE scintillation vial (Noviglio, Italy). The bound radioactivity has been determined by liquid scintillation counting.^33,42^

#### Opioid Receptor Binding Affinity

MOR, DOR, and KOR binding experiments were performed using [^3^H]-DAMGO (48.4 Ci/mmol), [^3^H]-(2-D-Ala)-[Tyrosyl-3,5-]-DELTORPHIN II (54.7 Ci/mmol), and [^3^H]-U69,593 (49.3 Ci/mmol), respectively (PerkinElmer, Belgium). Unlabeled naloxone hydrochloride, DAMGO, (−)-U50,488, and naltrindole hydrochloride were purchased from Sigma-Aldrich (St. Louis, MO, USA). MOR and DOR binding experiments were carried out by incubating 200 μg/sample of rat brain membranes for 45 min at 35 °C with 1 nM [^3^H]-DAMGO (*K*_d_ 1.0 nM) or 2 nM [^3^H]-(2-d-Ala)-[Tyrosyl-3,5-]-DELTORPHIN II (*K*_d_ 1.5 nM) in 50 mM Tris–HCl (pH 7.4). For KOR binding assays, guinea pig brain membranes (200 μg/sample) have been incubated with 1 nM [^3^H]-U69,593 (*K*_d_ 2.3 nM) for 30 min at 30 °C. Test compounds were added in a final volume of 0.5 mL. Non-specific binding has been measured using 10 μM unlabeled naloxone. The reaction was stopped by rapid filtration under reduced pressure using Millipore filter apparatus through Whatman glass fiber filters (GF/C for MOR and DOR GF/B for KOR) presoaked in a 0.1% poly(ethyleneimine) solution. Filters were washed with 50 mM ice-cold Tris–HCl buffer (3 × 2 mL), dried, and soaked in 3 mL of scintillation cocktail in a 4 mL Kartell HDPE scintillation vial (Noviglio, Italy). The radioactivity was detected by a liquid scintillation counter (Beckman LS 6500).^43^

#### S1R Functional Assay

Binding experiments were performed using the same procedure for the S1R binding assay in the presence of phenytoin (1 mM) or its solvent (NaOH 0.3 M) in a final volume of 0.5 mL. Experiments were carried out by incubating rat liver homogenates at 37 °C for 2 h. The test compound is defined an S1R agonist if the *K*_i_ ratio without/with phenytoin is >1 and an S1R antagonist if the *K*_i_ ratio without/with phenytoin is ≤1.^36^

#### Data Analysis

The *K*_i_ values were calculated with the program GraphPad Prism 9.0 (San Diego, CA, USA). The *K*_i_ values are given as mean value ± SD from at least two independent experiments performed in duplicate.

### Selectivity Profiling

The selectivity profile of compound **9d** was assessed at 1 μM in a small panel of recognized human targets by Eurofins Panlabs Discovery Services according to their standard assay protocols (https://www.eurofinsdiscovery.com/).

#### Cannabinoid CB1 receptor

Human recombinant cannabinoid CB1 receptors expressed in rat hematopoietic Chem-1 cells were used in modified HEPES buffer pH 7.4. Experiments were carried out by incubating 5 μg/sample of membranes with [^3^H]SR141716A (2 nM, *K*_d_ 18 nM) for 60 min at 37 °C. CP 55,940 (10 μM) has been employed to assess non-specific binding. Bound and free radioligands were separated by fast filtration, filters were washed four times, and the trapped radioactivity was counted to determine [^3^H]SR141716A specifically bound.

#### Cannabinoid CB2 receptor

Human CB2 receptor-expressing CHO-K1 cells were used in modified HEPES buffer pH 7.0. Experiments were carried out by incubating 30 μg/sample of membranes with [^3^H]WIN-55,212-2 (2.4 nM, *K*_d_ 4.9 nM) for 90 min at 37 °C. R(+)-WIN-55,212-2 (10 μM) has been employed to assess non-specific binding. Bound and free radioligand were separated by fast filtration and the trapped radioactivity was counted to determine [^3^H]WIN-55,212-2 specifically bound.

#### NMDA Receptor

Rat cerebral cortical membranes of male Wistar were used in HEPES buffer pH 7.7. Experiments were carried out by incubating 2.5 mg/sample of membranes with [^3^H]MDL 105,519 (0.33 nM, *K*_d_ 6 nM) for 30 min at 4 °C. MDL 105,519 (10 μM) has been employed to assess non-specific binding. Bound and free radioligands were separated by fast filtration, and the trapped radioactivity was counted to determine [^3^H]MDL 105,519 specifically bound.

#### 5-HT2A Receptor

CHO-K1 cells stably transfected with a plasmid encoding the human serotonin 5-HT2A receptor were used to prepare membranes in modified Tris–HCl pH 7.4 buffer. Experiments were carried out by incubating 30 μg/sample of membranes with [^3^H]Ketanserin (0.5 nM, *K*_d_ 0.2 nM) for 60 min at 25 °C. Mianserin (1 μM) has been employed to assess non-specific binding. Bound and free radioligands were separated by fast filtration, and the trapped radioactivity was counted to determine [^3^H]Ketanserin specifically bound.

#### SERT receptor

HEK-293 cell membranes stably transfected with a plasmid encoding the human serotonin transporter were prepared in modified Tris–HCl pH 7.4 buffer. Experiments were carried out by incubating 9 μg/sample of membranes with [^3^H]Paroxetine (0.4 nM, *K*_d_ 0.078 nM) for 60 min at 25 °C. Bound and free radioligands were separated by fast filtration, and the trapped radioactivity was counted to determine [^3^H]Paroxetine specifically bound.

### Molecular Modeling

#### Active and Decoy Compounds

Fifteen ligands with *K*_i_ spanning from 0.005 to 5 nM on the S1R and fifteen compounds with *K*_i_ spanning from 0.12 to 8.2 nM on the S2R were considered (compounds extrapolated from ChEMBL).^44^ The decoy set has been generated with the DUDE-Z online server, and two datasets of 750 and 850 decoys for S1R and S2R were generated.^45^

#### Ligand Preparation

The LigPrep tool was used for all the compounds preparation. Salts were removed, hydrogens were added, and the states of ionization at pH 7.4 were calculated using Epik. The internal energy of the conformers was estimated through the OPLS_2005 force field (LigPrep, Schrödinger, LLC, New York, NY, 2018).^46^

#### Receptor Preparation and Validation

The crystal structure of the S1R was retrieved from the Protein DataBank. We employed the human protein bound to PD144418 (PDB ID 5HK1).^27^ The receptor structure was properly processed using the Protein Preparation Wizard tool.^47^ Disulfide bonds were created, and hydrogens were added. The hydrogen-bonding network was optimized, and the p*K*_a_ of the residues along with their protonation state were calculated at pH 7.4. The structure reveals a trimeric architecture; however, only the protomer with the most complete sequence was selected for our study. Molecular dynamics was performed using Desmond package v. 3.8.^48^ The protomer was inserted in a fully hydrated palmitoyl-oleyl-phosphatidylcholine (POPC) bilayer, the system was immersed in an orthorhombic box of TIP4P water molecules, extending at least 10 Å from the protein, and counter ions were added to neutralize the system charge. The system temperature was set at 300 K, and the NPT ensemble was selected. The simulation was carried out for 100 ns, and the trajectories and energies were recorded at 100 ps intervals. The resulting trajectory was clustered with respect to the root mean square deviation (RMSD), getting four cluster representatives. Using the MacroModel tool and OPLS-2005 as the force field, these structures were submitted to 10,000 iterations of energy minimization,^46^ thus obtaining 4 additional structures for subsequent molecular recognition studies.

The crystal structure of the S2R was retrieved from the Protein DataBank. We employed the bovine protein bound to compound Z1241145220 (PDB ID 7M95).^17^ To obtain the human wild-type structure of the S2R, 37 residues of the bovine receptor were appropriately mutated (T3A, L4P, G5A, A6T, G9C, L10V, F13L, F16L, L27F, G32A, D37E, L42F, Q47L, Q48K, I51A, E52K, T61E, A85T, F89L, G92S, L118F, L120F, D121E, H128G, R130K, G133R, K135E, F137L, Q138H, F142T, I144V, I148A, F151L, L155F, L159I, V162I, N164S) and the resulting model was used for the subsequent studies (see Figure S1 for sequence alignment).

The structure was refined using the Protein Preparation Wizard tool.^47^ Disulfide bonds were created, and hydrogens were added. The hydrogen-bonding network was optimized, and the p*K*_a_ of the residues along with their protonation state were calculated at pH 7.4. A molecular dynamics simulation was performed using the same conditions described for the S1R. The trajectory clusterization produced six cluster representatives, which were minimized, thus obtaining 12 structures.

#### Validation of the Docking Protocol

The validation of each S1R and S2R cluster representative was carried out, and the enrichment factor, the AUC, and the receiver operating characteristic (ROC) were analyzed. For the docking studies, we selected the S1R and S2R structures associated with the highest AUC and ROC values (S1R AUC value: 0.76 and ROC value: 0.77; S2R AUC value: 0.89 and ROC value: 0.89 – Figures S2 and S3 and Tables S2 and S3 for more details).

#### Docking Studies

Molecular docking was carried out with Glide v. 6.7, using the Standard Precision (SP) protocol and generating 10 poses per ligand.^49^

### In Vitro Toxicity

#### Cell Culture

Human corneal epithelial (HCE) cells were provided by Deepak Shukla (University of Illinois at Chicago, Chicago, IL, USA). HCE cells were cultured in a Medium Essential Media (MEM, Corning, Cellgro, Manassas, VA, USA) supplemented with 10% fetal bovine serum (FBS, Gibco Life Technologies, Grand Island, NY, USA) and 1% penicillin–streptomycin as reported prior. Standard cell culture conditions (37 °C, 5% CO_2_, >95% humidity) were used during routine passages, as done previously.

#### Cytotoxicity and Cell Viability of HCE Cells

HCE cells were seeded in a 96-culture well plate and were grown to 80–90% confluence. HCE cells were incubated with various concentrations of compounds in free medium for 20 h. Each analysis was repeated three times, and the results are expressed as the means of three independent experiments.

#### LDH Assay

The permeability of cellular membranes following the exposures was determined by measuring the amount of released LDH (lactate dehydrogenase) enzyme from HCE cells. The commercial CytoTox 96 kit (Promega, Fitchburg, WI, USA) was used according to the manufacturer’s instructions. To measure maximal LDH release, 10 μL of 10× lysis solution was added to control wells 45 min before adding the CytoTox 96 reagent. To measure the amount of released LDH, 50 μL of each well was transferred to a fresh 96-well plate and 50 μL of CytoTox 96 reagent was added followed by a 30 min incubation period. Finally, 50 μL of stop solution was added and absorbance was recorded at 490 nm (Synergy H1 Hybrid Reader, BioTek, Winooski, VT, USA). Absorbance values were corrected by background values, and the percentage of LDH release was calculated using the following formula: 100 × experimental LDH release optical density (OD)/maximum LDH release OD.

#### MTT Assay

Cellular viability was analyzed by the MTT (3-(4,5-dimetnythiazol-2-yl)-2,5-diphenyl-thetazolium bromide, Sigma-Aldrich Co.) assay. Briefly, fresh MTT solution (5 mg/mL in 1× PBS) was added (1:5 volume of medium) to the treated and non-treated cells for 1 h. The formazan precipitate was dissolved in 100 μL of DMSO (Sigma-Aldrich Co.), and absorbance at 540 nm was read on a microplate reader as a measure of cell viability. The cell viability was described as the percentage of the control group values. The percentage of cell viability was calculated as follows: 100 × mean OD in treated cells/mean OD in untreated cells.

#### Statistical Analysis

Unless otherwise stated, all experiments were performed with triplicate samples and repeated at least three times. The results are expressed as the mean ± standard deviation (SD) and analyzed using one-way analysis of variance (ANOVA) followed by Dunnett’s tests for multiple comparisons or unpaired Student’s *t* tests for two-group comparisons. All analyses were performed using Prism 6.0 (GraphPad Software, San Diego, CA, USA), and *p* values <0.05 were considered statistically significant.

### In Vivo Studies

#### Experimental Animals

Experiments were performed in female WT-CD1 mice (Charles River, Barcelona, Spain) weighing 25–30 g. Mice were acclimated in our animal facilities for at least 1 week before testing and were housed in a room under controlled environmental conditions: 12/12 h day/night cycle, constant temperature (22 ± 2 °C), air replacement every 20 min, and they were fed a standard laboratory diet (Harlan Teklad Research Diet, Madison, WI, USA) and tap water ad libitum until the beginning of the experiments. The behavioral test was conducted during the light phase (from 9:00 to 15:00 h) and randomly throughout the estrous cycle. Animal care was in accordance with institutional (Research Ethics Committee of the University of Granada, Spain), regional (Junta de Andalucía, Spain), and international standards (European Communities Council Directive 2010/63).

#### Drugs and Drug Administration

The experimental compounds were dissolved in 5% DMSO (Merck KGaA, Darmstadt, Germany) in physiological sterile saline (0.9% NaCl). As S1R control drugs, we used the S1R antagonist BD-1063 1-[2-(3,4-dichlorophenyl)ethyl]-4-methylpiperazine dihydrochloride) and the S1R receptor agonist PRE-084 (2-(4-morpholinethyl)1]-phenyl cyclohexane carboxylate hydrochloride) (both provided by Cayman Chemical, Ann Arbor, Michigan, USA).^50^ Drug solutions were prepared immediately before the start of the experiments and injected s.c. in a volume of 5 mL/kg into the interscapular area. BD-1063 or the experimental drugs tested were injected 30 min before the administration of capsaicin (used as the chemical algogen). To test for the effects of PRE-084 on the antiallodynia induced by the other drugs, it was administered 5 min before the latter. Capsaicin was dissolved in 1% DMSO in physiological sterile saline to a concentration of 0.05 μg/μL. Capsaicin solution was injected intraplantarly (i.pl.) into the right hind paw proximate to the heel, in a volume of 20 μL (i.e., 1 μg per mouse) using a 1710 TLL Hamilton microsyringe (Teknokroma, Barcelona, Spain) with a 301/2-gauge needle. Control animals were injected with the same volume of the vehicle of capsaicin.

#### Evaluation of Capsaicin-Induced Secondary Mechanical Hypersensitivity

Animals were placed for 2 h in individual black-walled test compartments, which were situated on an elevated mesh-bottom platform with a 0.5 cm^2^ grid to provide access to the ventral surface of the hind paws. Punctate mechanical stimulation was applied with a Dynamic Plantar Aesthesiometer (Ugo Basile, Varese, Italy) 15 min after the administration of capsaicin or saline (i.e., 45 min after drug injection). Briefly, a nonflexible filament (0.5 mm diameter) was electronically driven into the ventral side of the right hind paw at least 5 mm away from the site of the injection toward the fingers. The intensity of the stimulation was set at 0.5*g* force.^30^ When a paw withdrawal response occurred, the stimulus was automatically terminated, and the response latency was recorded. The filament was applied three times, separated by intervals of 0.5 min. The mean value of the three trials was considered the withdrawal latency time of the animal. A cutoff time of 50 s was used in each trial.

#### Rotarod Test

Motor coordination was assessed with an accelerating rotarod (Cibertec, Madrid, Spain), as previously described.^34^ Briefly, mice were required to walk against the motion of an elevated rotating drum at increasing speed (4–80 rpm over 5 min), and the latency to fall was recorded with a cut-off time of 300 s. Mice were given three training sessions 24 h before drug testing. On the day of the test, rotarod latencies were measured immediately before the drug or vehicle (saline 5% DMSO) was administered (time 0) and several times (45, 90, 150, and 240 min) after the s.c. drug administration. As a comparison drug, we used pregabalin (60 mg/kg, s.c.), which has been reported to impair rotarod performance.^34^

### Chemical Stability

Evaluation of chemical stability for compound **9d** has been performed in 50 mM Phosphate Buffer (pH 7.4) as previously reported.^33^ The working solution was incubated at 37 ± 0.5 °C, and at appropriate time intervals, an amount of the reaction mixture was withdrawn and added of ACN. Three individual experiments were run in triplicate. The half-life (t_1/2_) of compound **9d** was determined by fitting the data with one phase exponential decay equation using GraphPad Prism 9.0 (San Diego, CA, USA).

### Water Solubility

Aqueous solubility was determined by UHPLC-PDA analysis.^33^ First, 5 mg of **9d** (free base) was weighed and added to 1 mL of ultrapure water. The suspension was shaken at rt for 24 h and then centrifuged, and the supernatant was filtered and diluted in MeOH before analysis. The compound is quantified against a methanol calibration curve built over seven dilution concentrations.

### Plasma Stability

Human plasma was obtained by centrifugation of blood samples containing 0.3% citric acid.^51^ Plasma fractions were diluted with 0.02 M phosphate buffer (pH 7.4) and incubated at 37 °C. The reaction started by adding 200 μL of a stock solution of **9d** to preheated plasma. At appropriate time intervals, 100 μL aliquots were taken and deproteinized by mixing with 0.01 M HCl in MeOH. After centrifugation, the clear supernatant was analyzed by UHPLC-PDA. The amounts of the remaining intact compounds were plotted as a function of incubation time. The experiments were run in triplicate, and the mean values of the rate constants were calculated.

### Metabolic Stability

*In vitro* metabolic stability studies were performed by Eurofins Cerep Quality Control Unit (France) using mouse and human liver microsomes fractions in accordance with their validation Standard Operating Procedure.

### Measure of hERG Activity

Electrophysiological experiments were performed in CHO e K1 cells that express human ERG using a Qube APC assay. Compound **9d** has been tested employing six different concentrations ranging from 10^–10^ to 10^–5^ M using serial dilution by Eurofins Panlabs (St Charles, MO, United States) according to their standard assay protocol.

## Supporting Information Available

Supplementary synthetic procedures; ^1^H NMR and ^13^C NMR spectra of final compounds; molecular modeling analysis; sequence alignment; validation of the docking protocol (PDF)
Molecular formula strings (CSV)
3D PDB file of S1R_9d (PDB)
3D PDB file of S1R_15a (PDB)
3D PDB file of S2R_9d (PDB)
3D PDB file of S2R_15a (PDB)

## Supplementary Material

jm3c00959_si_002.csv

jm3c00959_si_003.pdb

jm3c00959_si_004.pdb

jm3c00959_si_005.pdb

jm3c00959_si_006.pdb

## Notes

Patents (provisional and pending—as inventor or co-inventor), owned by the Board of Trustees of the University of Illinois and is an equity holder in ViSo Therapeutics Inc. (V.K.A.).

## ABBREVIATIONS

DOR: δ opioid receptor
HCE: human corneal epithelial cells
HDPE: high-density polyethylene
KOR: κ opioid receptor
LDH: lactate dehydrogenase
MOR: μ opioid receptor
OR: opioid receptor
S1R: sigma 1 receptor
S2R: sigma 2 receptor
SAR: structure–activity relationship
SRs: sigma receptors
TLC: thin-layer chromatography
