# Supplementary material for: Discovery of AD258 as a Sigma Receptor Ligand with Potent Antiallodynic Activity
Source: J Med Chem. 2023 Aug 3;66(16):11447–63. doi: 10.1021/acs.jmedchem.3c00959 (PMC10461227; doi:10.1021/acs.jmedchem.3c00959)
Supplement: Supplementary file 1 — jm3c00959_si_001.pdf [file jm3c00959_si_001.pdf]

Supporting Information

Discovery of AD258 as a Sigma Receptor Ligand with Potent Antiallodynic Activity

Maria Dichiara,[†] Francesca Alessandra Ambrosio,[‡] Sang Min Lee,[‖] M. Carmen Ruiz-Cantero,[#] Jessica Lombino,[†] Adriana Coricello,[§] Giosuè Costa,[§,⊥] Dhara Shah,[‖] Giuliana Costanzo,[†] Lorella Pasquinucci,[†] Kyung No Son,[ǂ] Giuseppe Cosentino,[†] Rafael González-Cano,[#] Agostino Marrazzo,[†] Vinay Kumar Aakalu,[ǂ] Enrique J. Cobos,[#] Stefano Alcaro,[§,⊥,*] and Emanuele Amata[†,*]

[†]Dipartimento di Scienze del Farmaco e della Salute, Università degli Studi di Catania, Viale Andrea Doria 6, 95125 Catania, Italy;

[‡]Dipartimento di Medicina Sperimentale e Clinica, Università degli Studi "Magna Græcia" di Catanzaro, Campus "S. Venuta", Viale Europa, 88100, Catanzaro, Italy;

[‖]Department of Ophthalmology and Visual Sciences, University of Illinois at Chicago, 1905 W. Taylor St, Chicago, IL 60612, United States;

[#]Departamento de Farmacología e Instituto de Neurociencias, Facultad de Medicina, Universidad de Granada e Instituto de Investigación Biosanitaria de Granada ibs.GRANADA, Avenida de la Investigación, 18016 Granada, Spain;

[§]Dipartimento di Scienze della Salute, Università "Magna Græcia" di Catanzaro, Campus "S. Venuta", 88100, Catanzaro, Italy;

[⊥]Net4Science Academic Spin-Off, Università "Magna Græcia" di Catanzaro, Campus "S. Venuta", 88100, Catanzaro, Italy.

[ǂ]Department of Ophthalmology and Visual Sciences, University of Michigan, 1000 Wall Street, Ann Arbor, Michigan, 48105, United States;

*Emanuele Amata: phone, (+39) 0957384102; email, eamata@unict.it

*Stefano Alcaro: phone, (+39) 09613694198; email, alcaro@unicz.it

Table of contents

^{1}H and ^{13}C NMR spectra	S2
Molecular modeling analysis	S21
Docking Score analysis (Table S1)	S21
Sequence alignment between human and bovine S2R (Figure S1)	S22
Count and percentage of actives in top N% of results for S1R (Table S2)	S22
Percent screen plot for S1R (Figure S2)	S22
Count and percentage of actives in top N% of results for S2R (Table S3)	S23
Percent screen plot for S2R (Figure S3)	S23

^{1}H and ^{13}C NMR spectra

NMR spectra in $CDCl_3$ from final compounds synthesized by the authors are included.

Compound 7b (AD214)

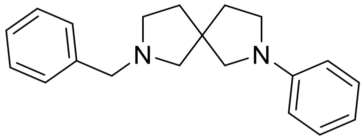

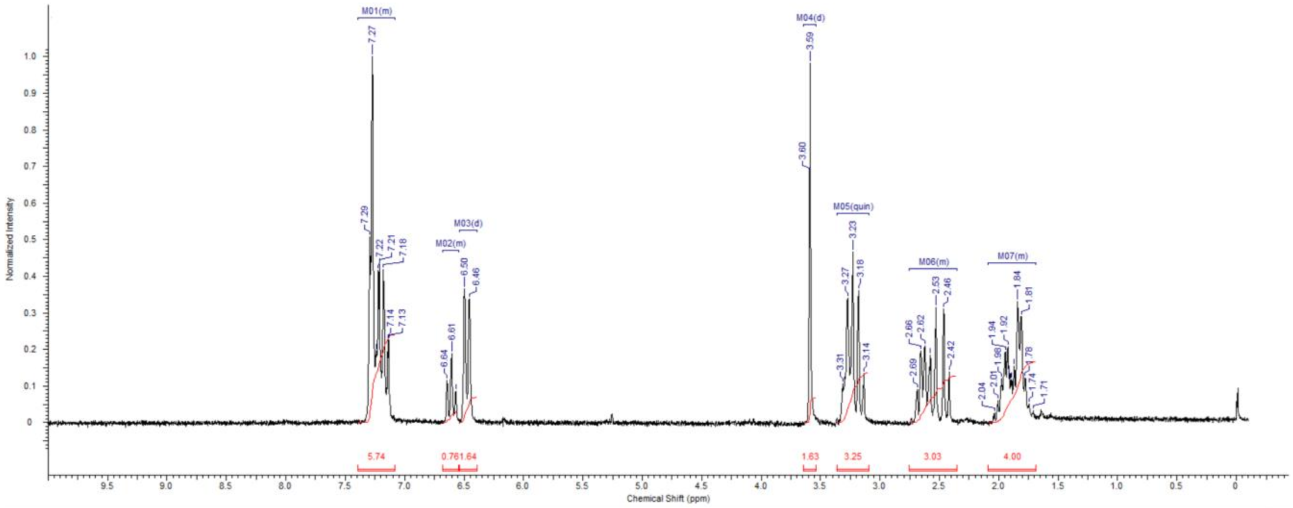

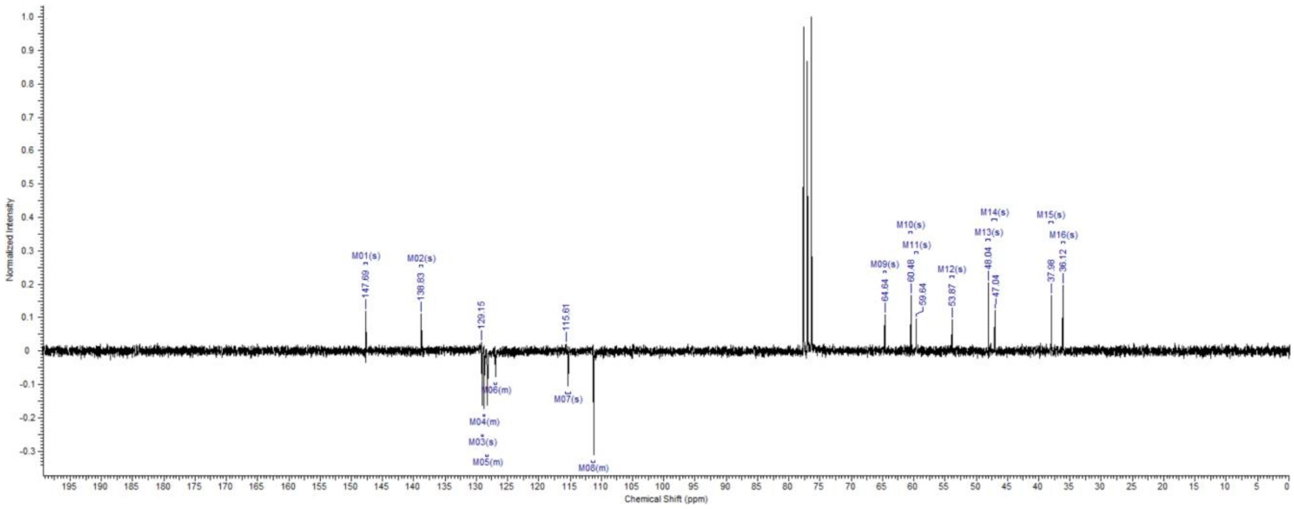

Compound 8a (AD174)

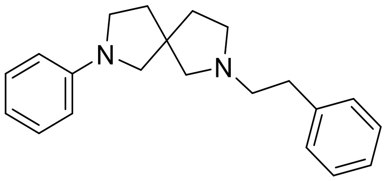

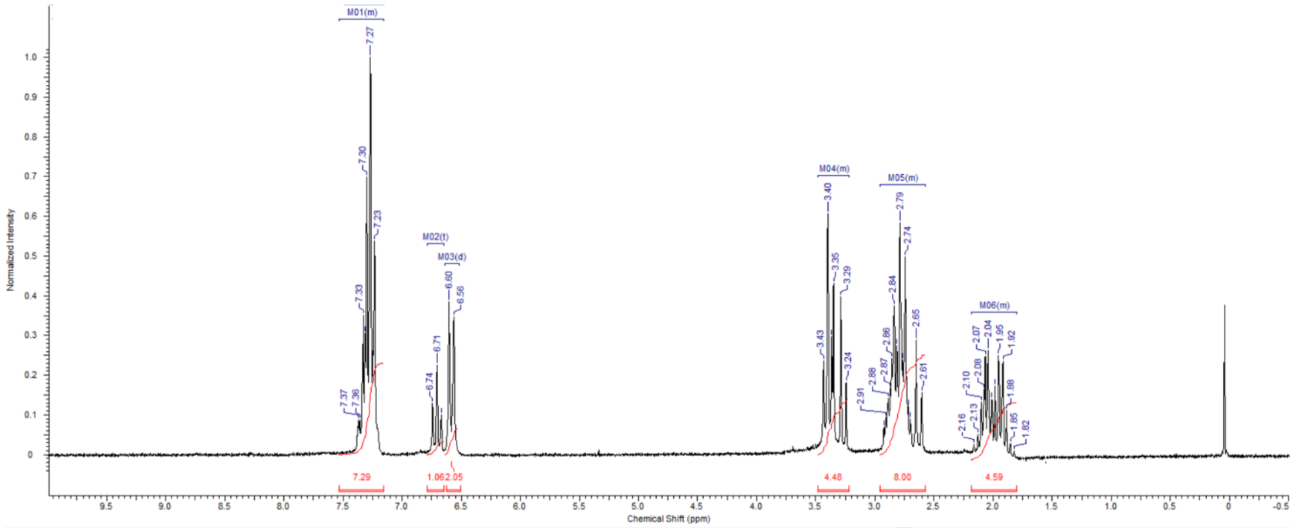

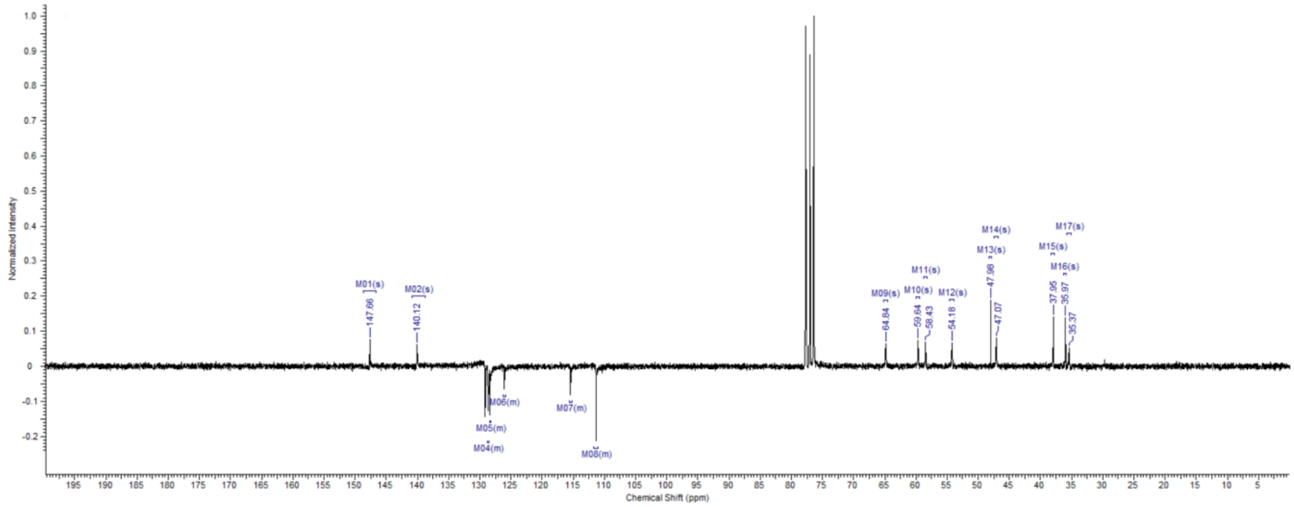

Compound 8b (AD157)

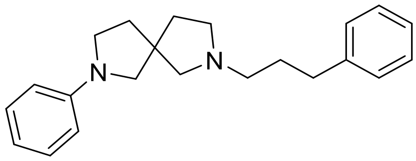

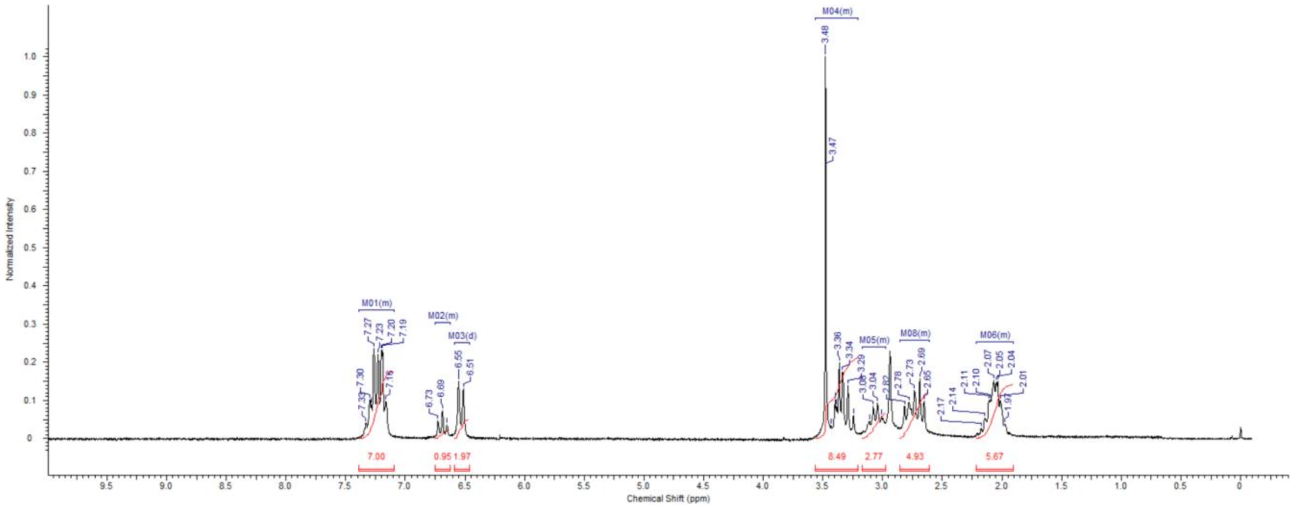

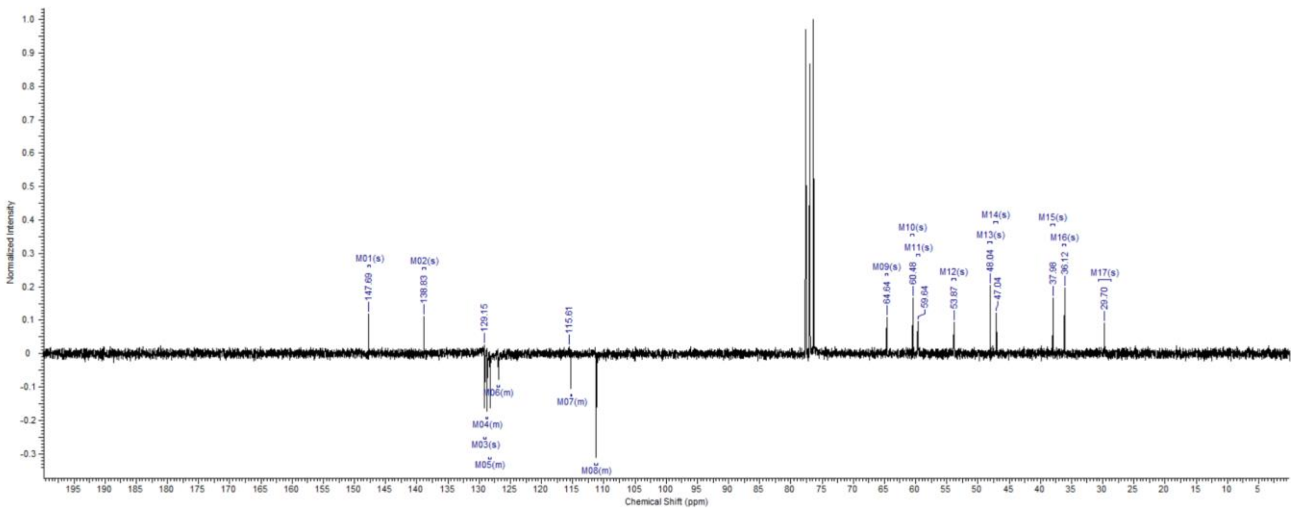

Compound 9a (AD242)

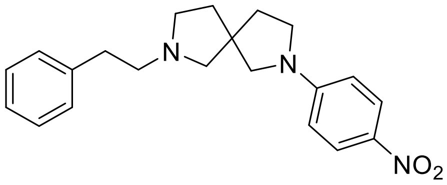

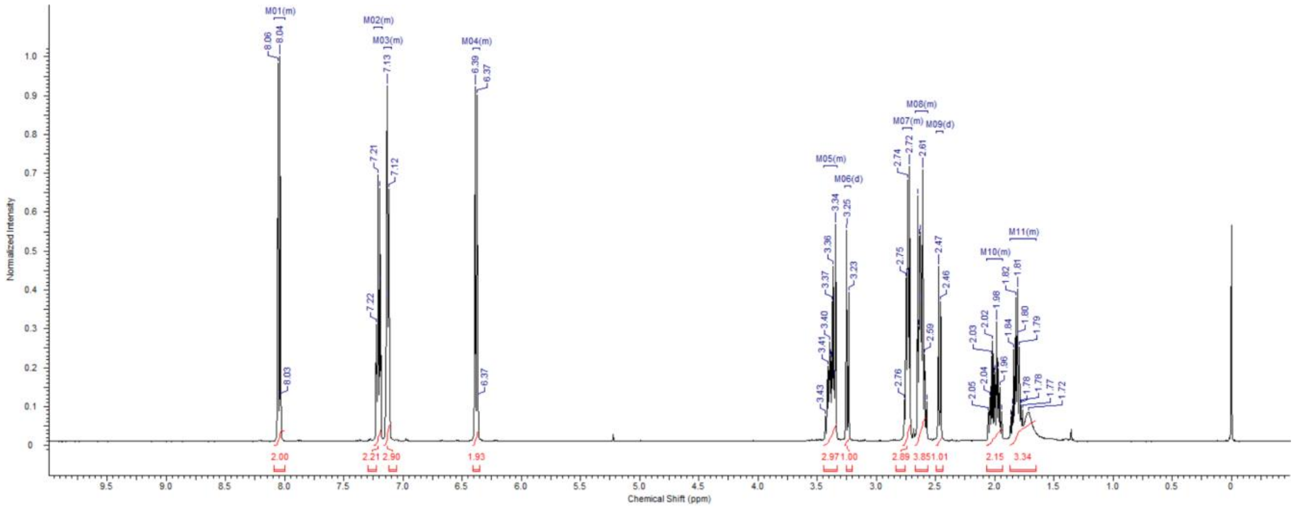

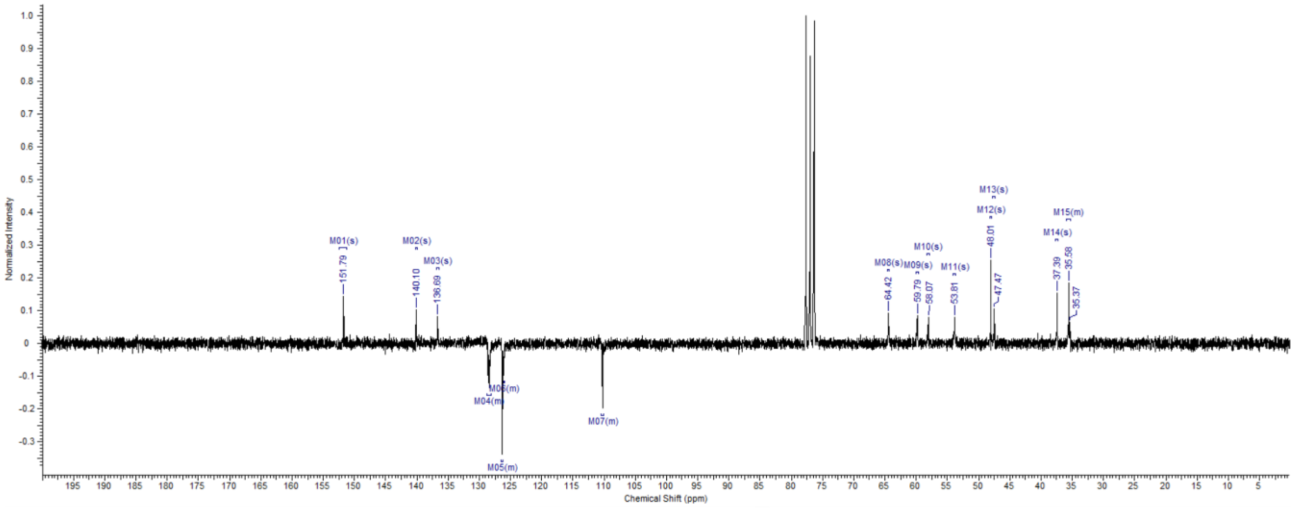

Compound 9b (AD239)

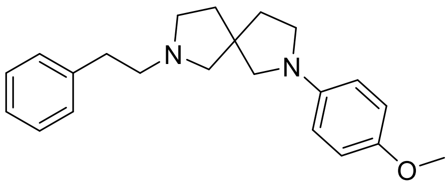

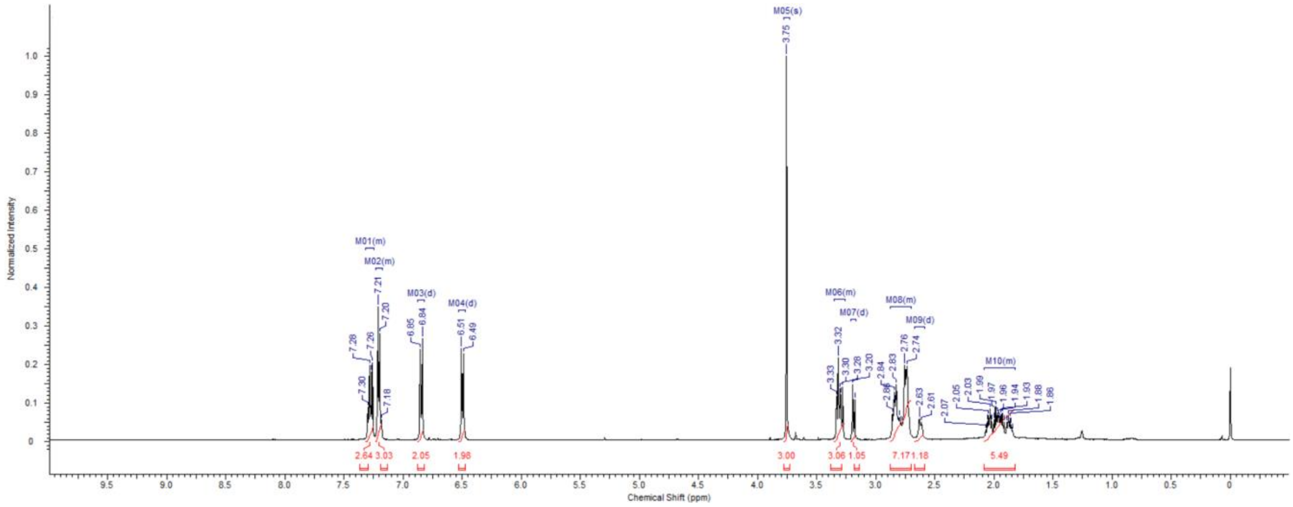

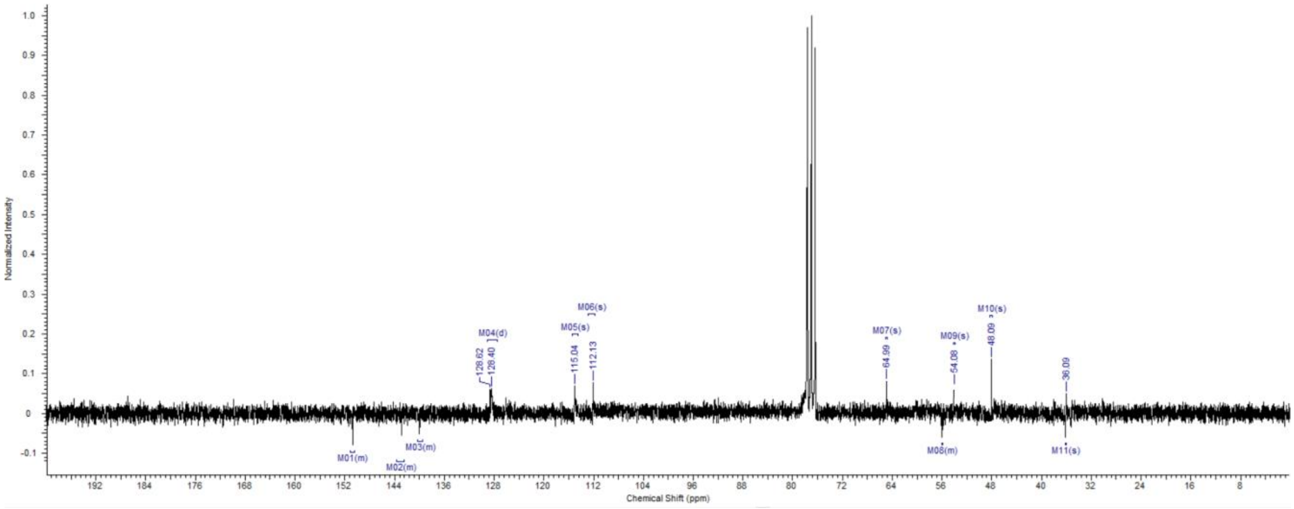

Compound 9c (AD245)

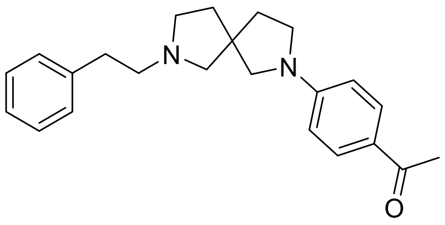

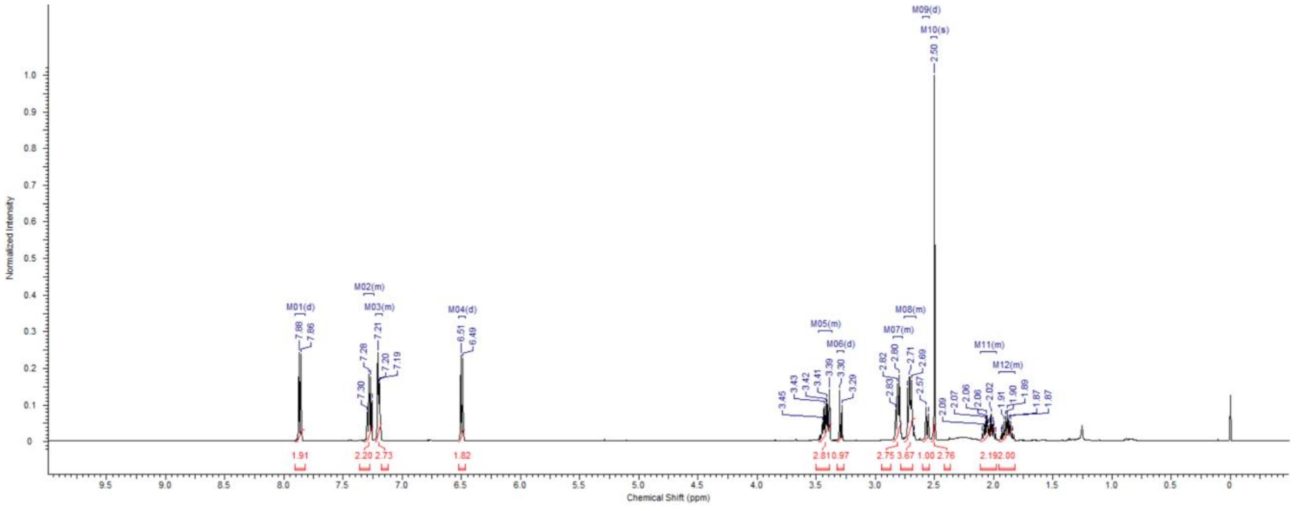

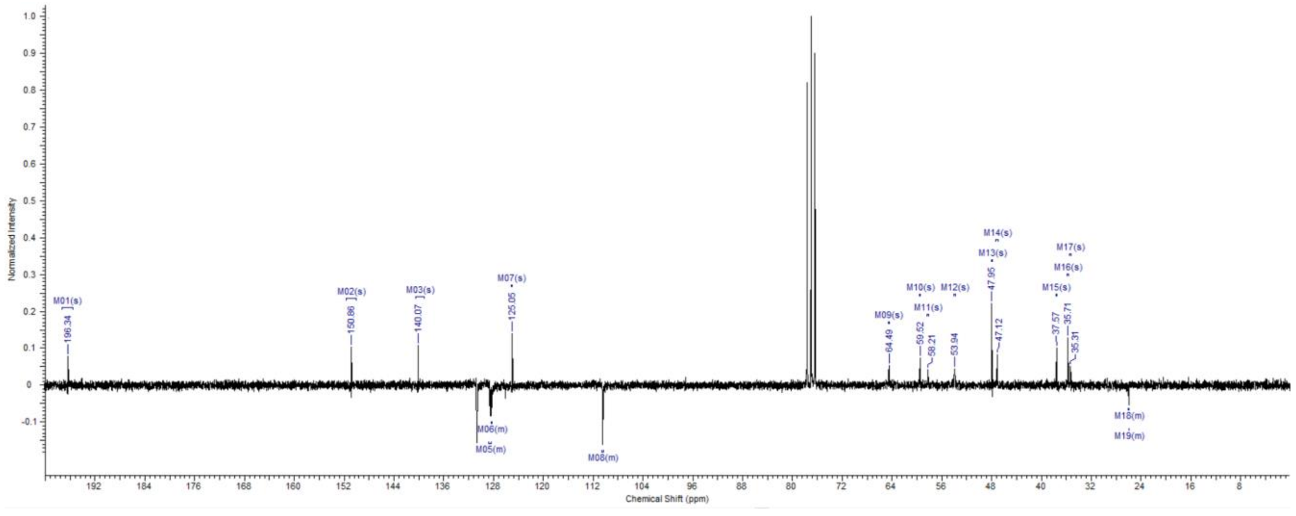

Compound 9d (AD258)

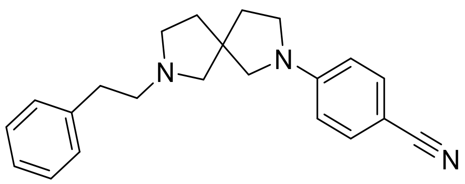

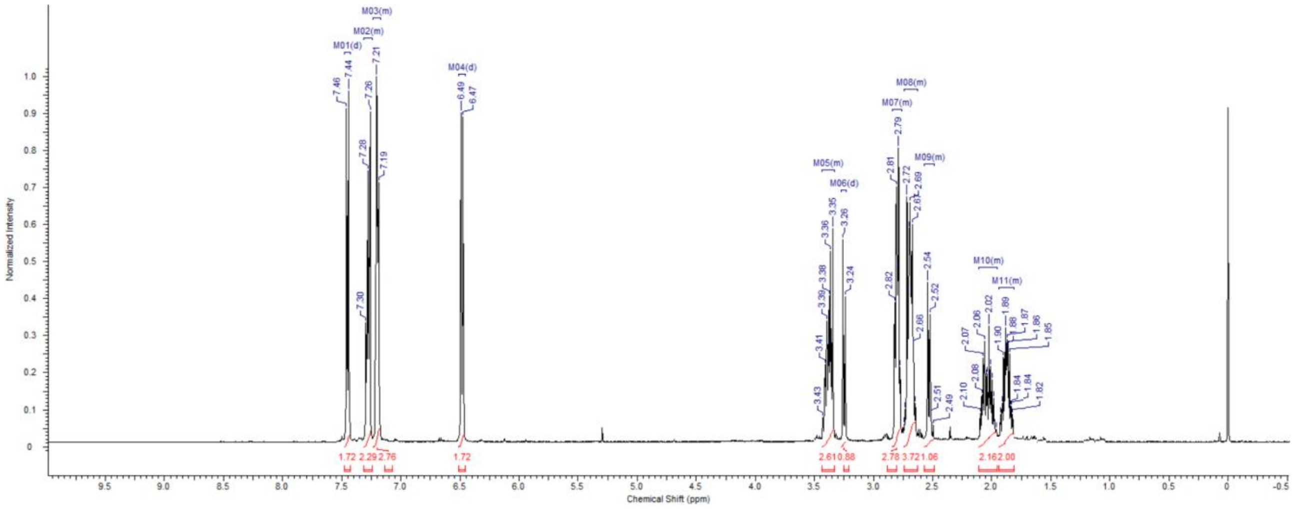

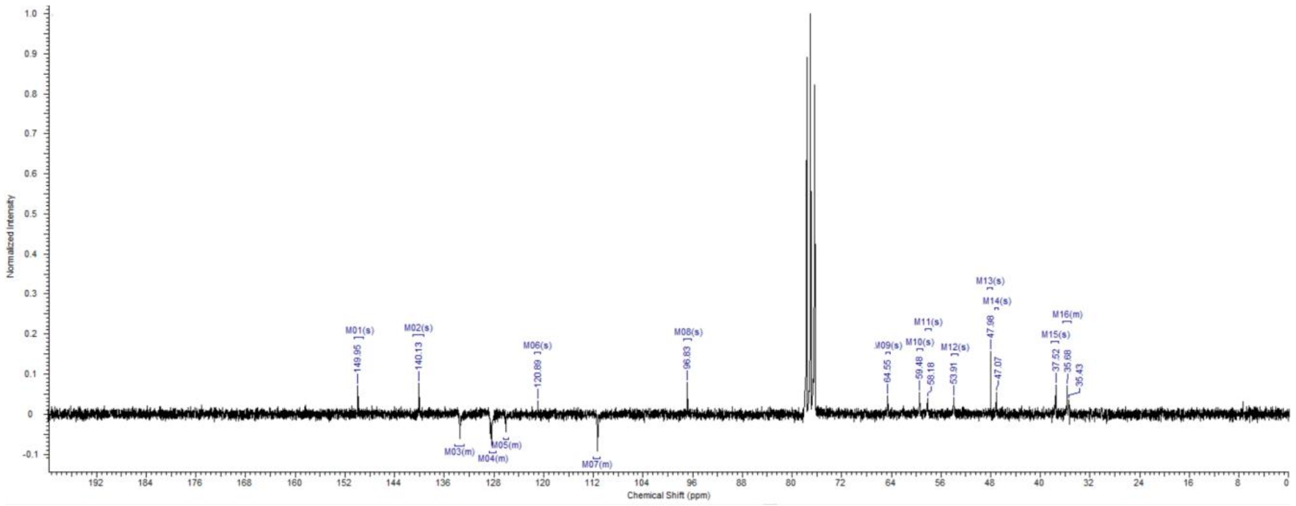

Compound 11b (AD206)

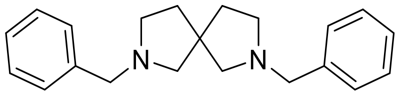

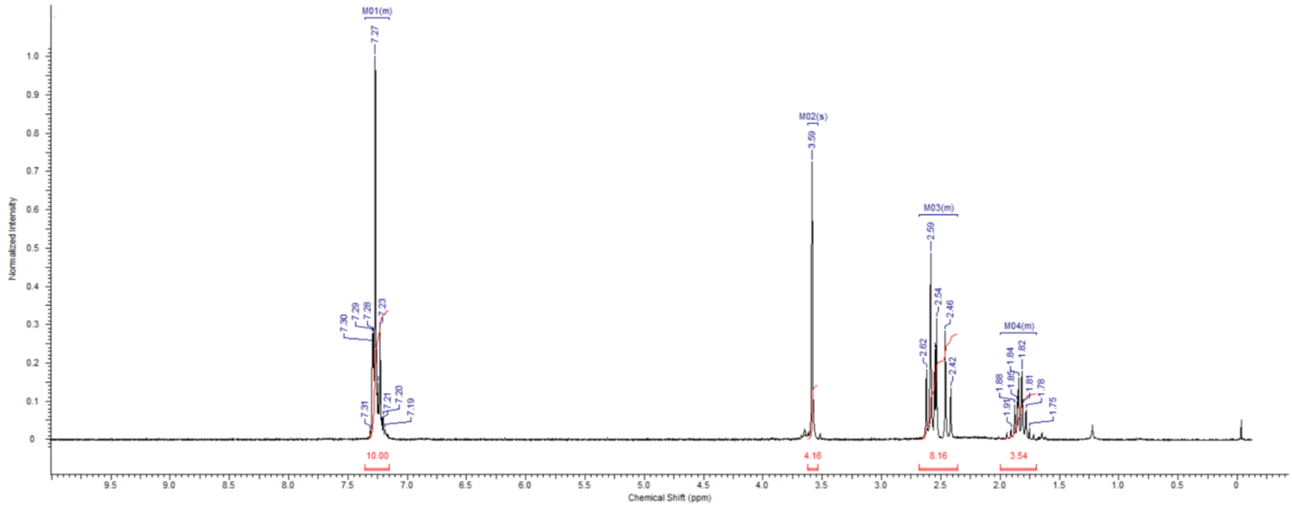

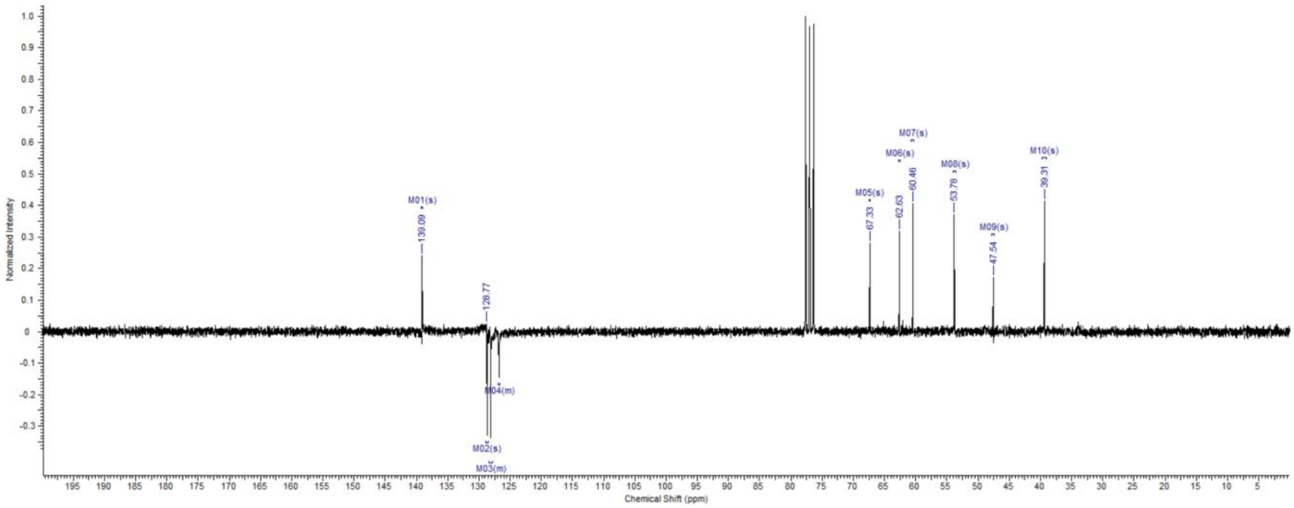

Compound 13a (AD145)

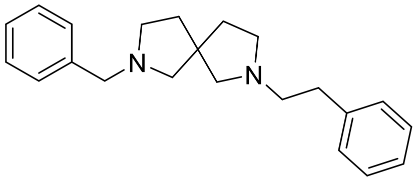

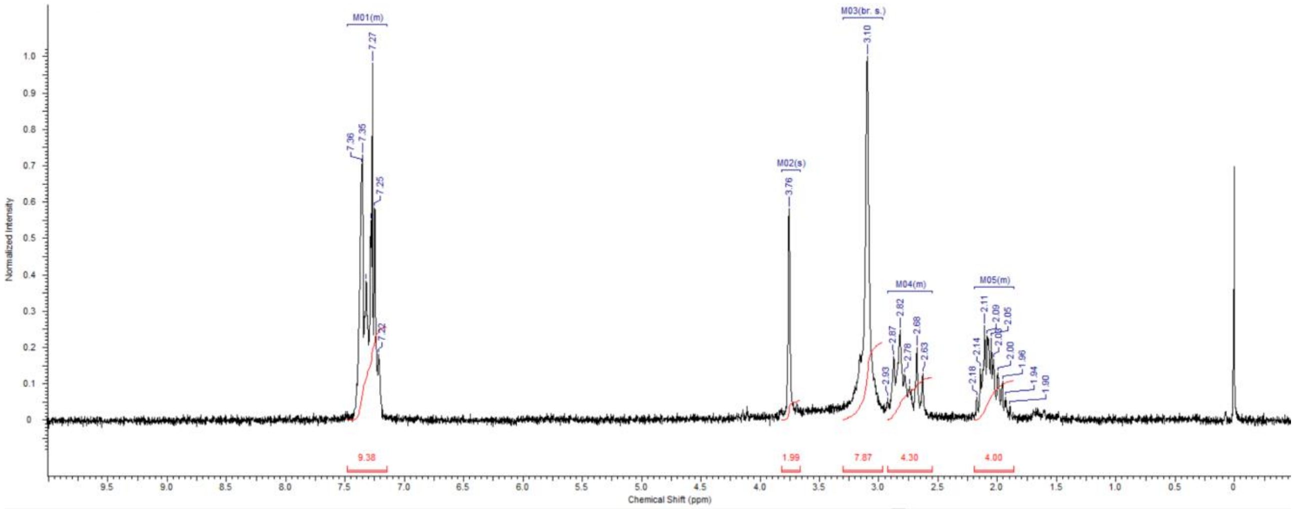

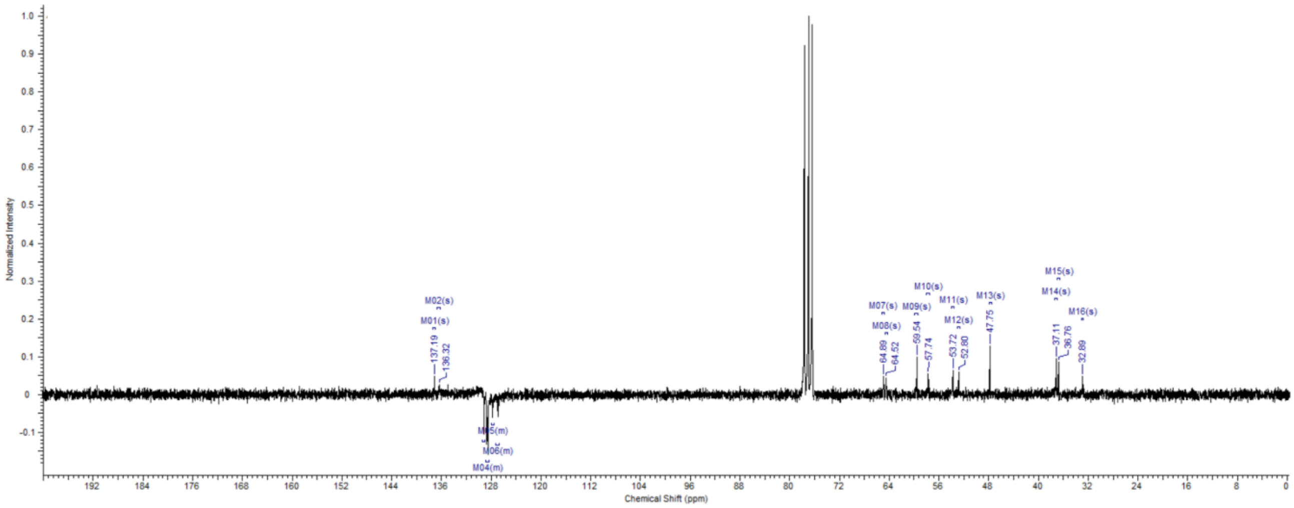

Compound 13b (AD193)

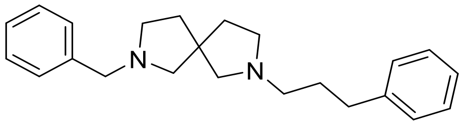

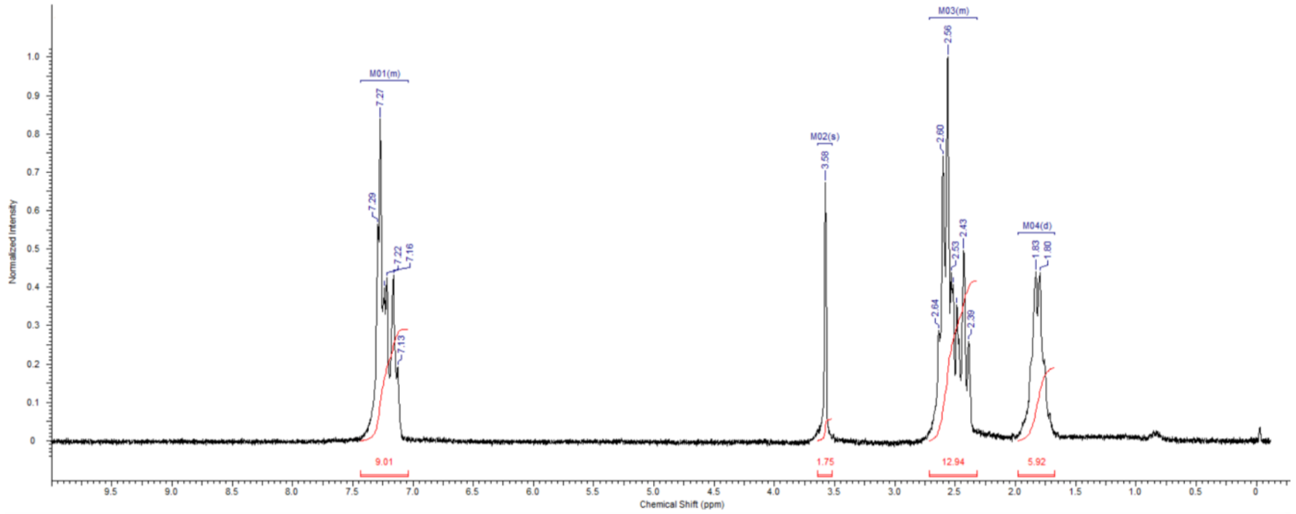

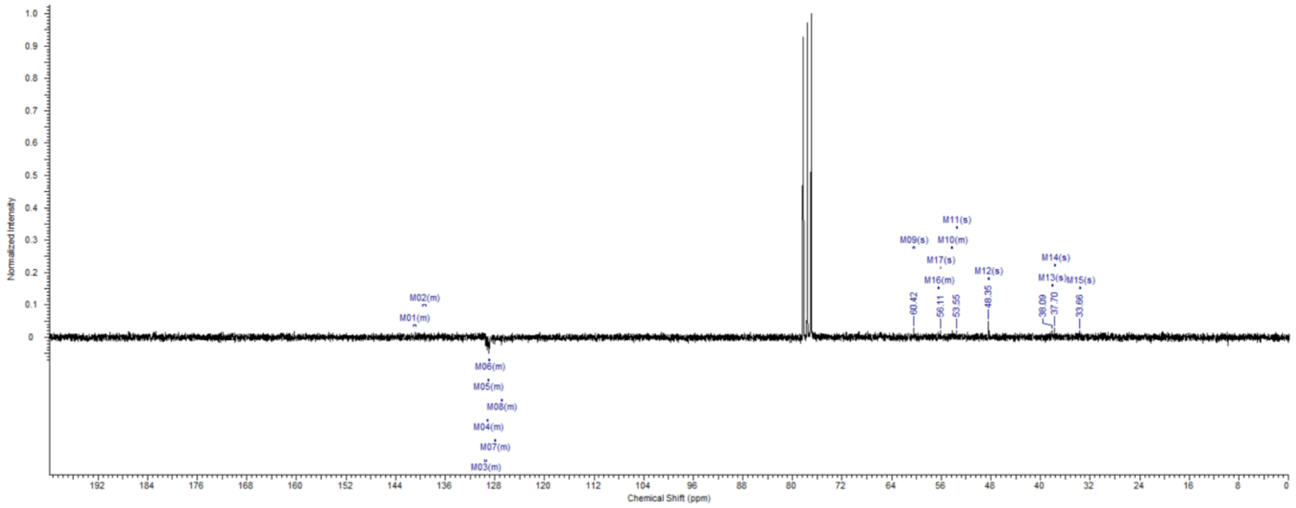

Compound 14a (AD181)

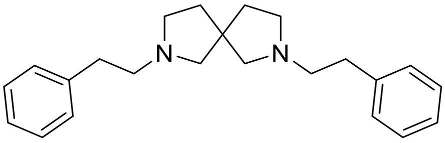

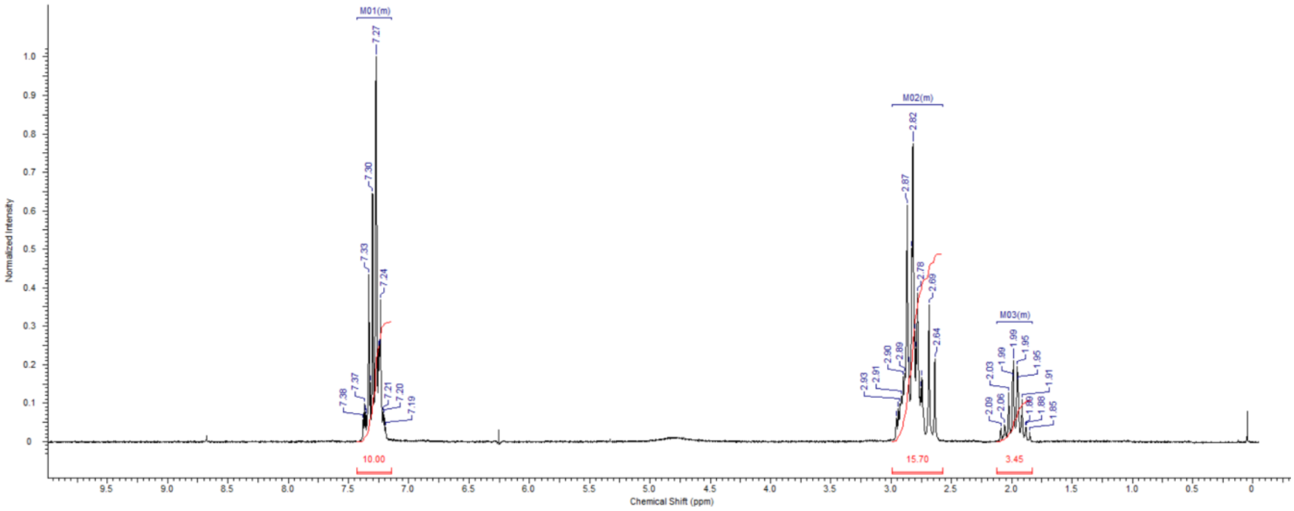

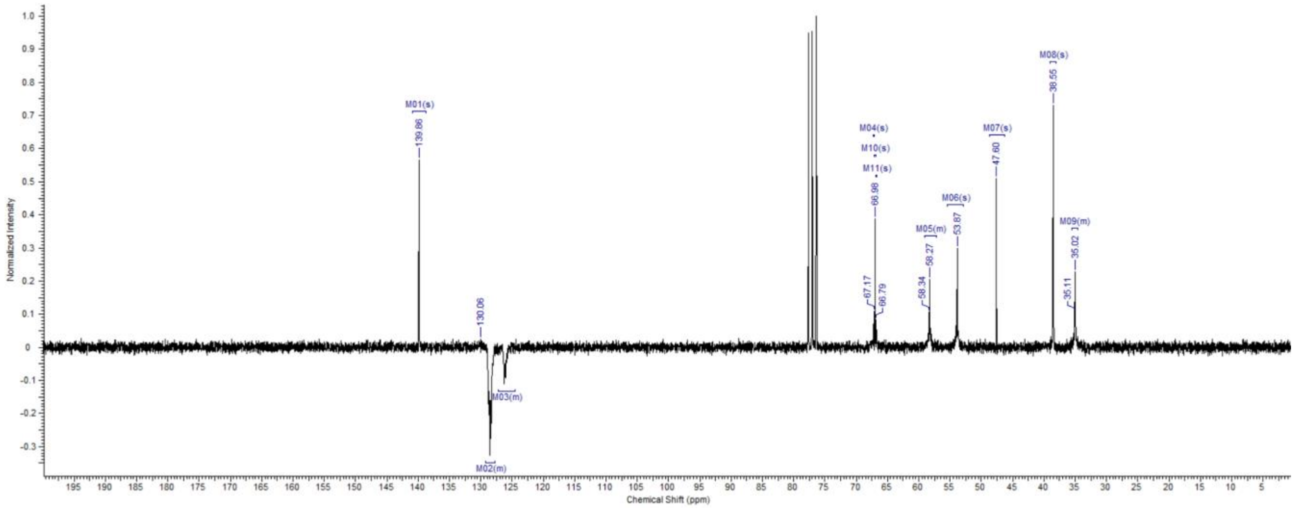

Compound 14b (AD182)

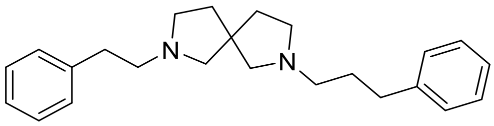

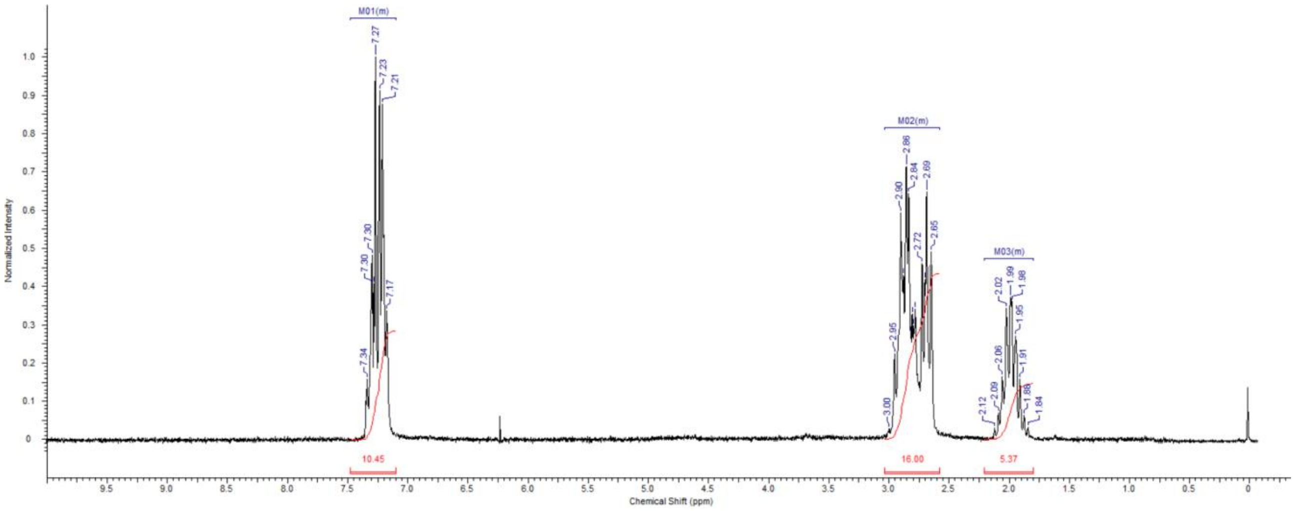

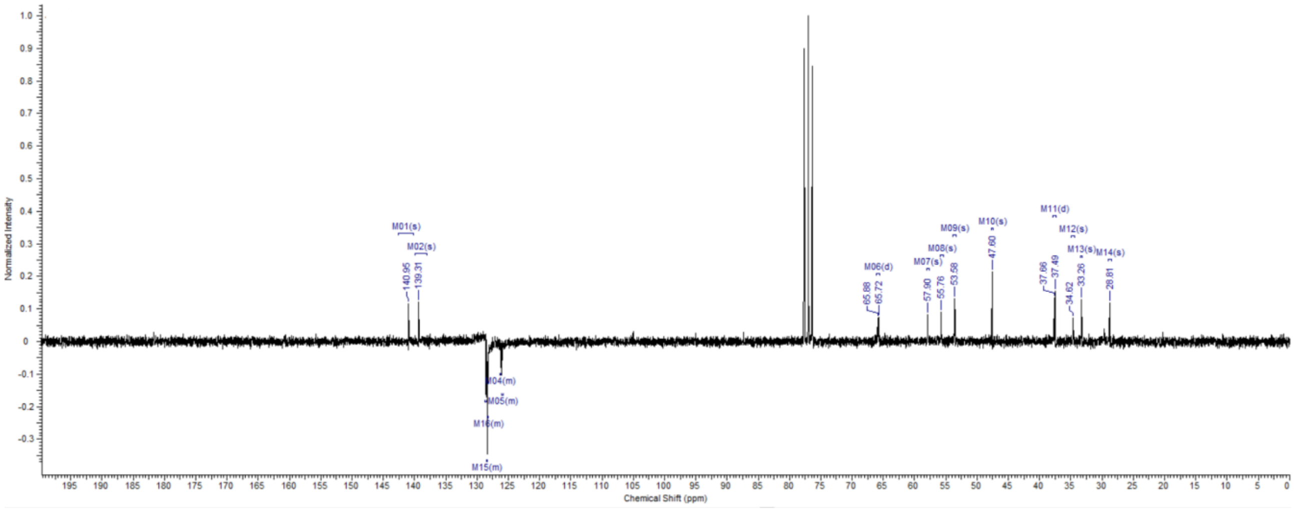

Compound 15a (AD220)

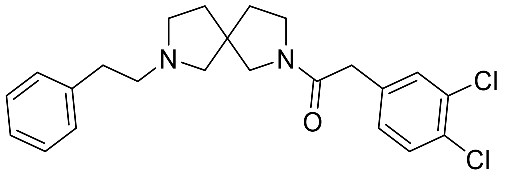

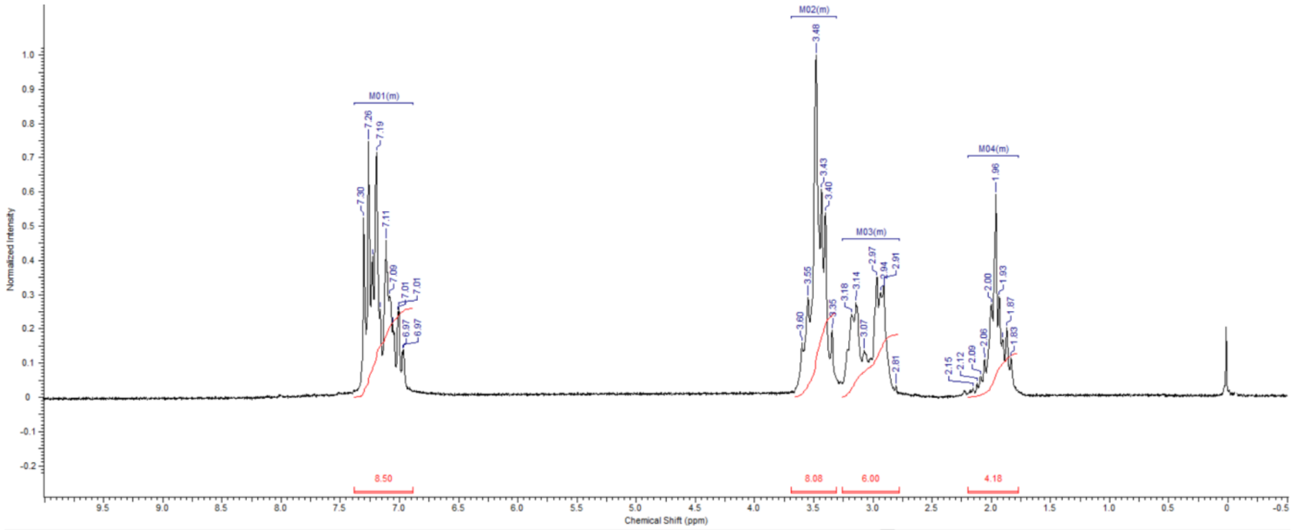

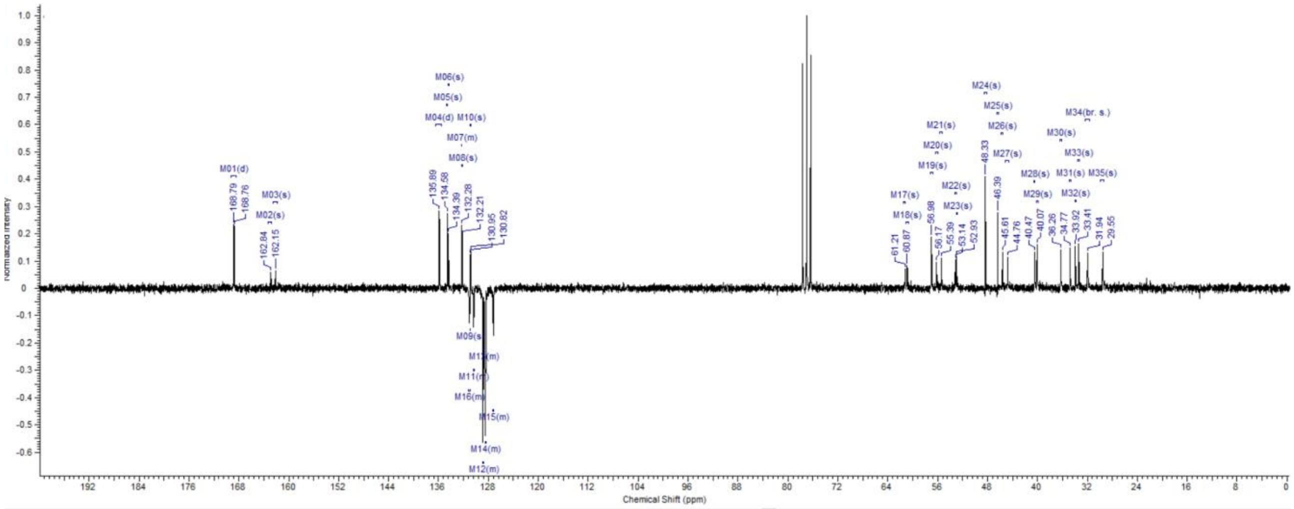

Compound 15b (AD226)

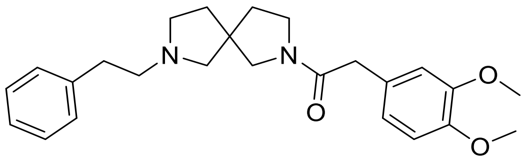

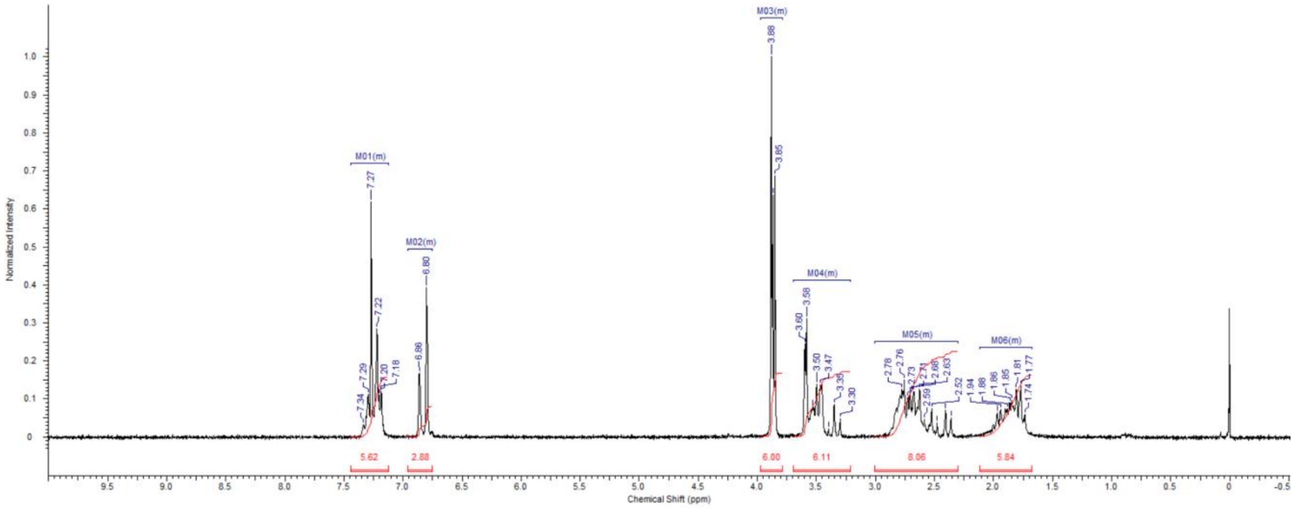

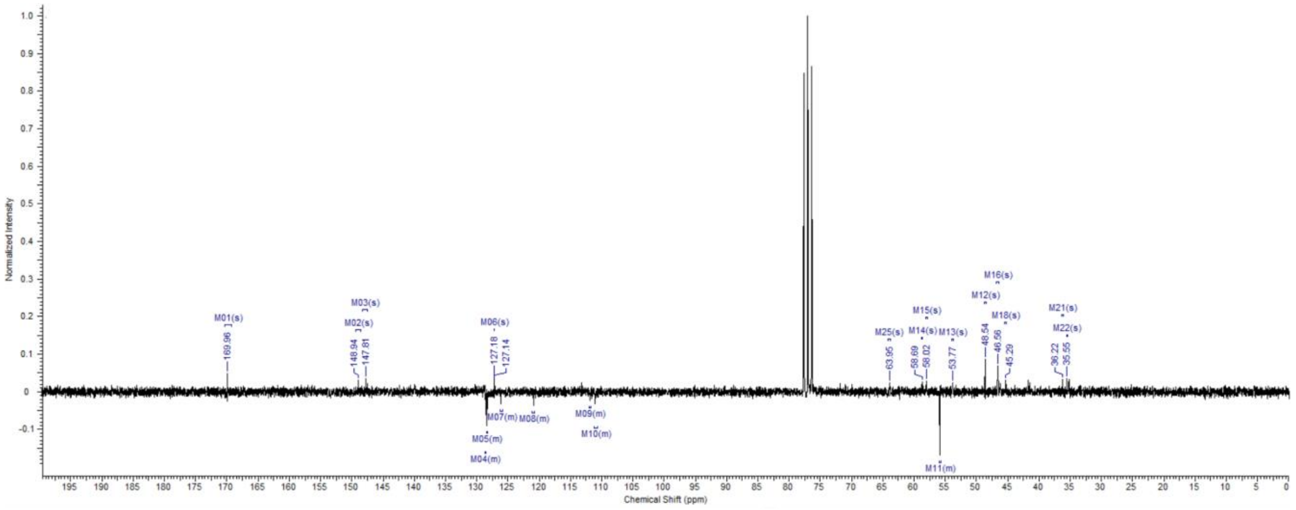

Compound 15d (AD217)

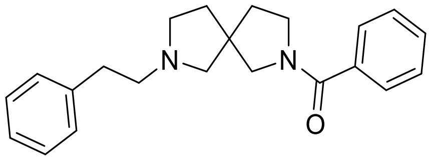

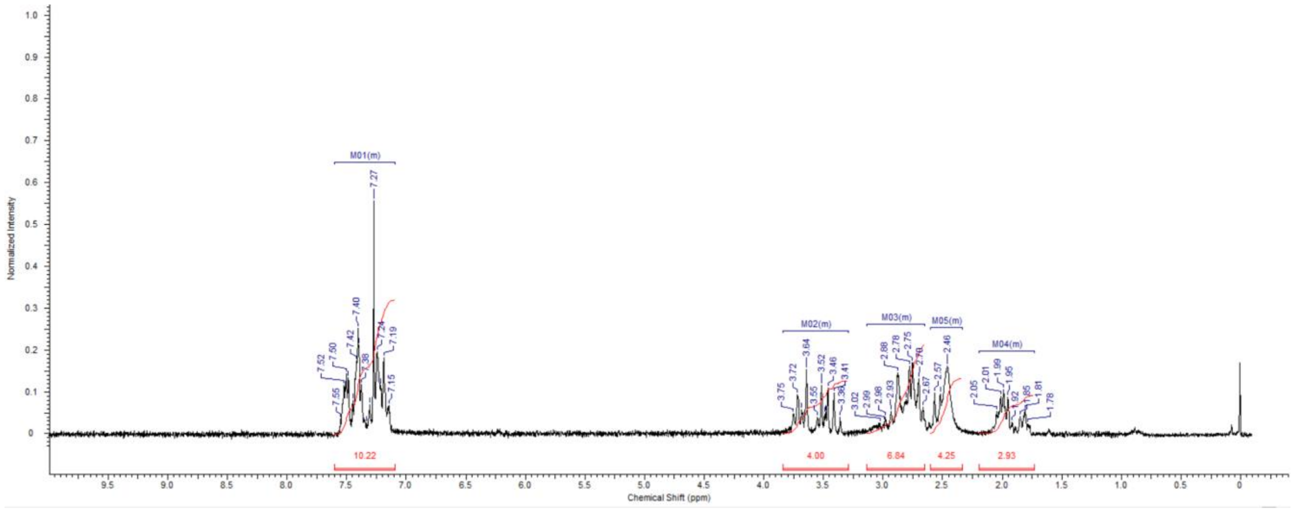

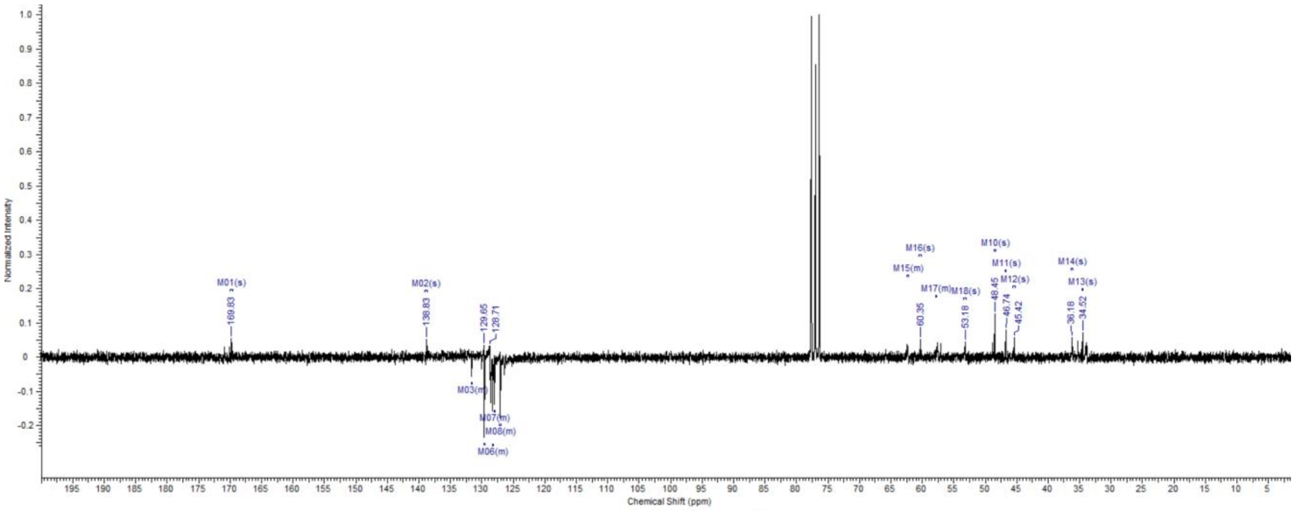

Compound 15e (AD219)

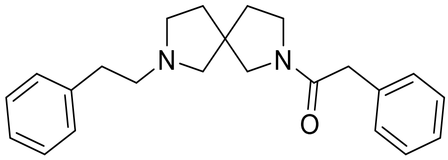

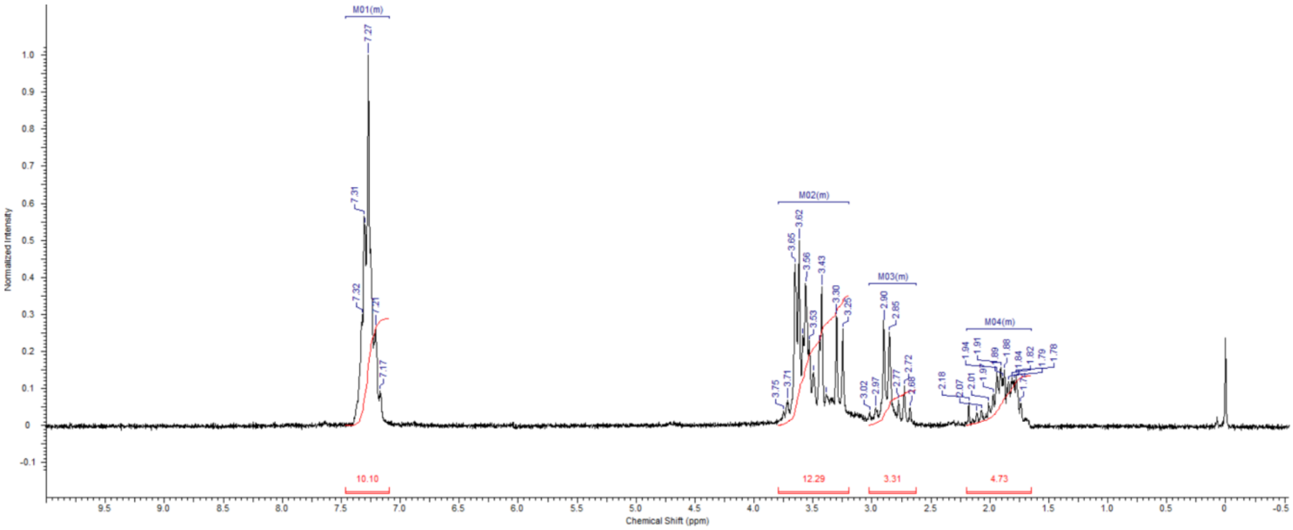

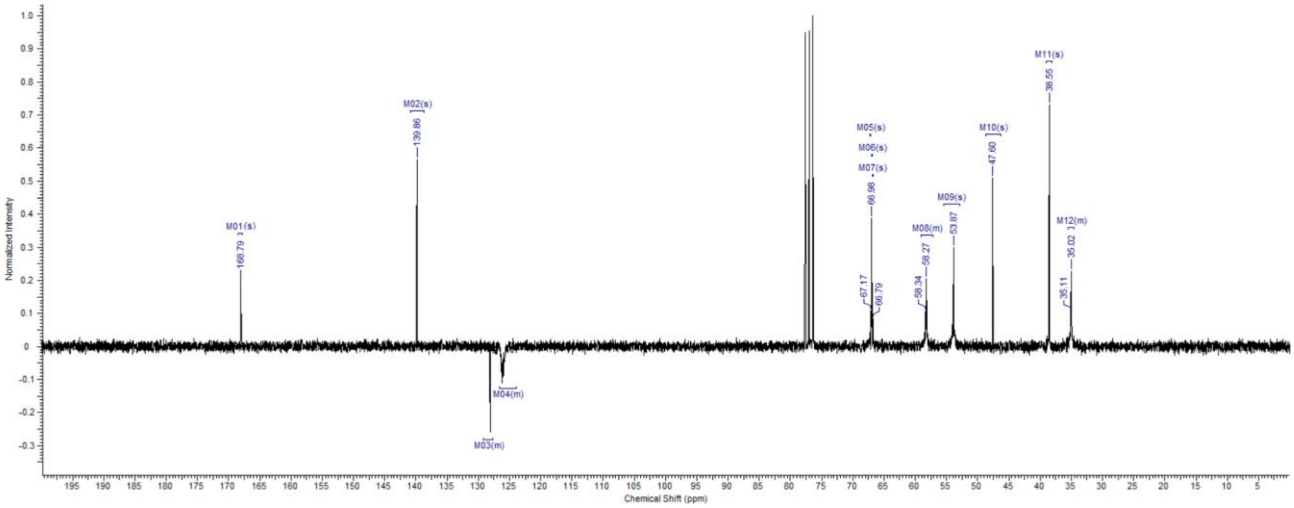

Compound 16a (AD225)

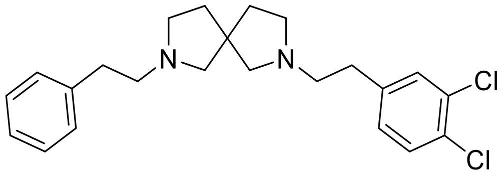

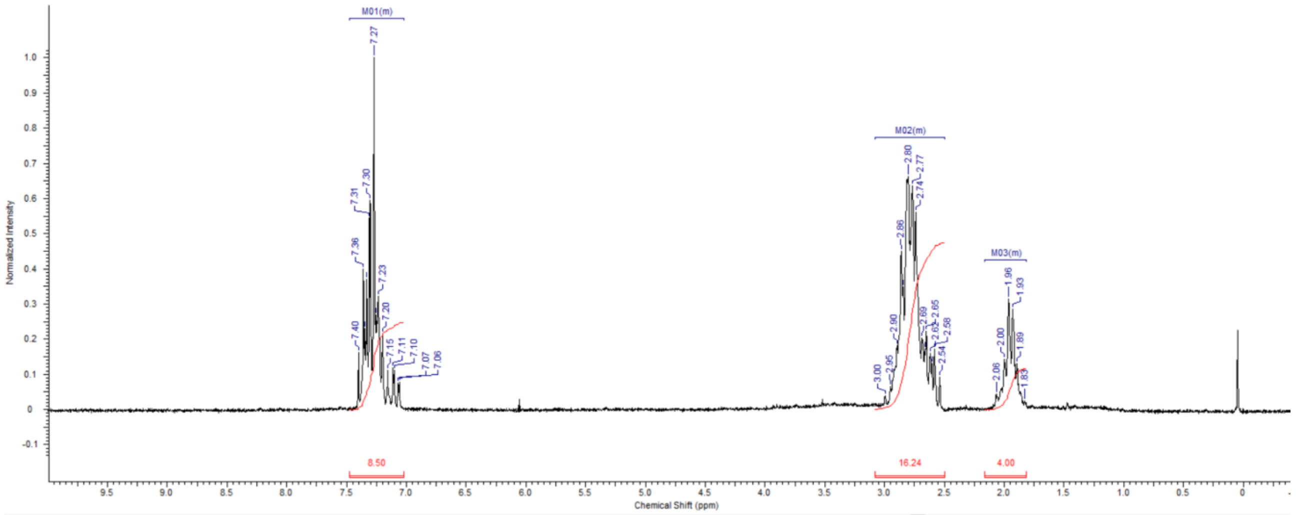

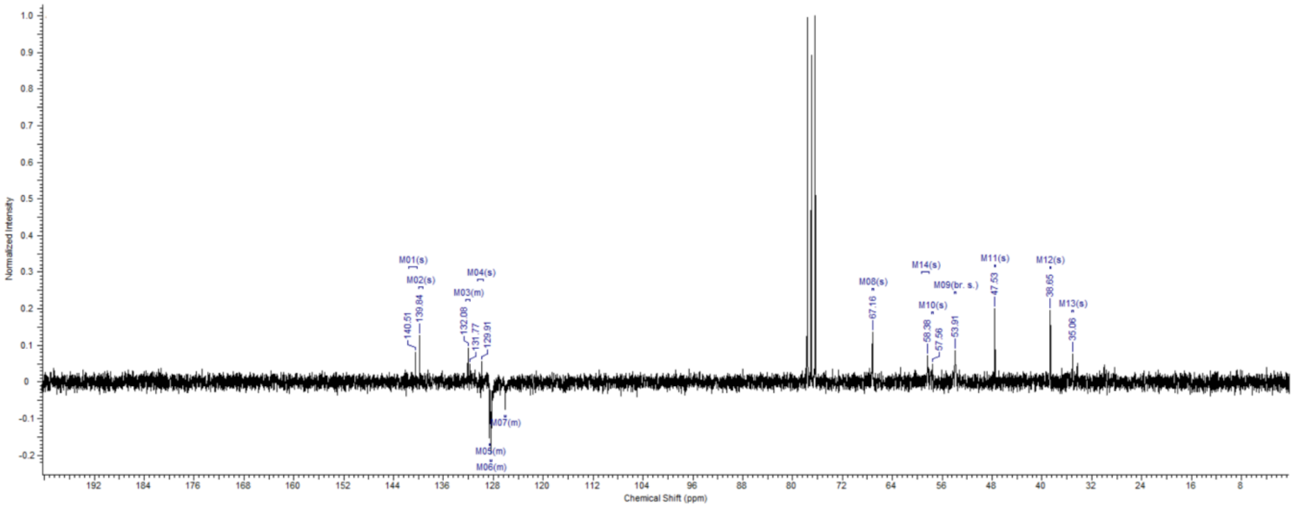

Compound 16b (AD234)

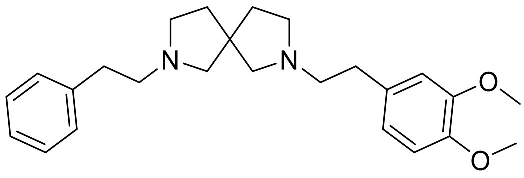

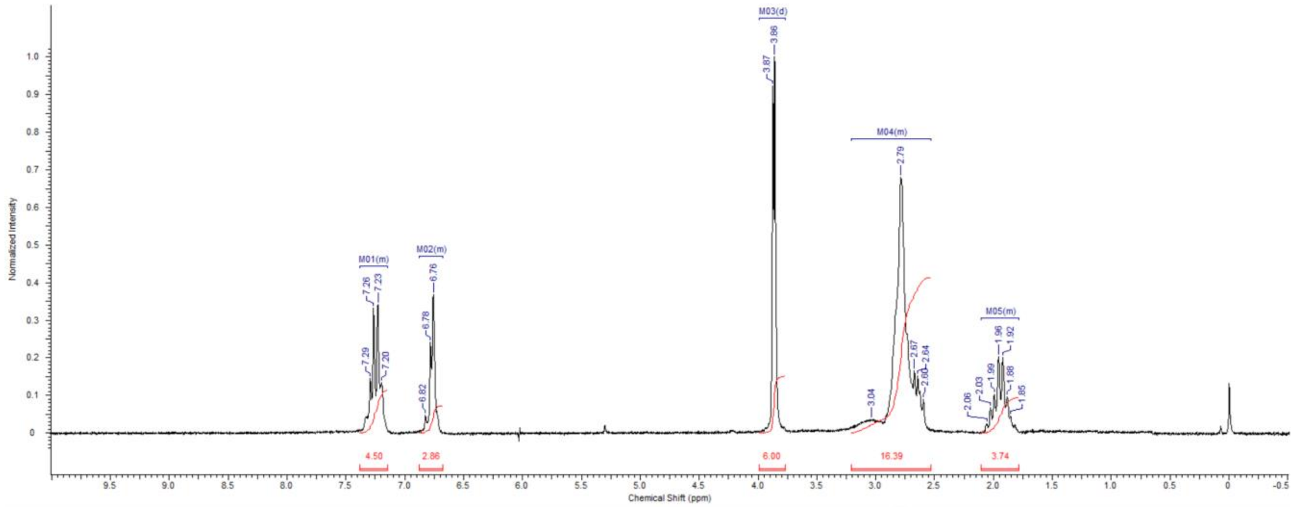

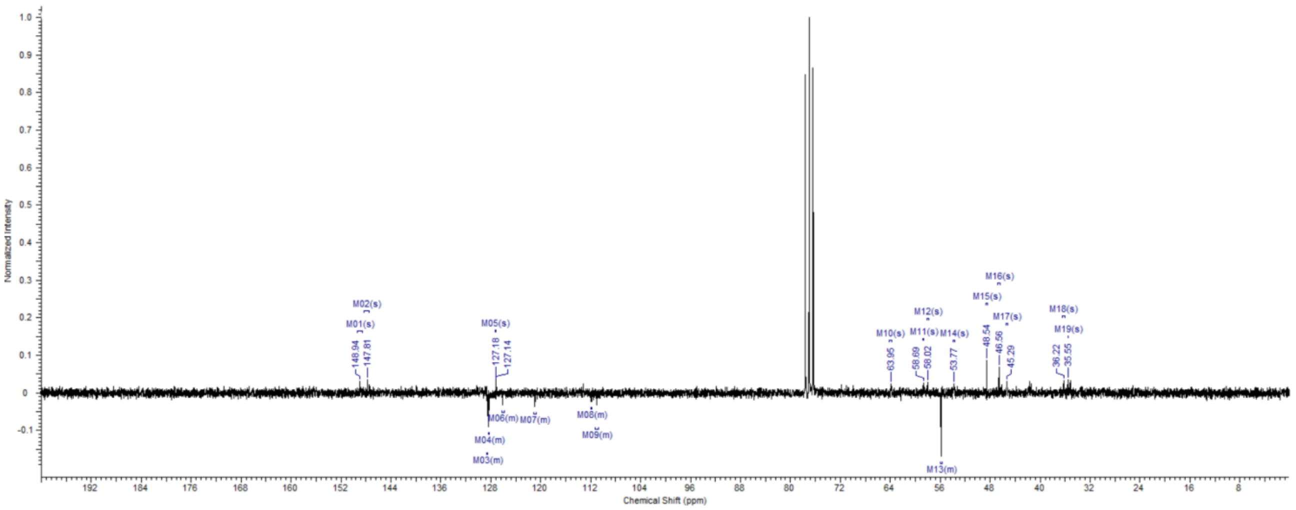

Compound 16b (AD267)

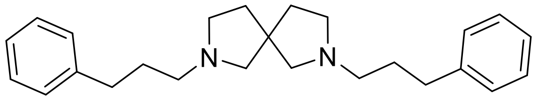

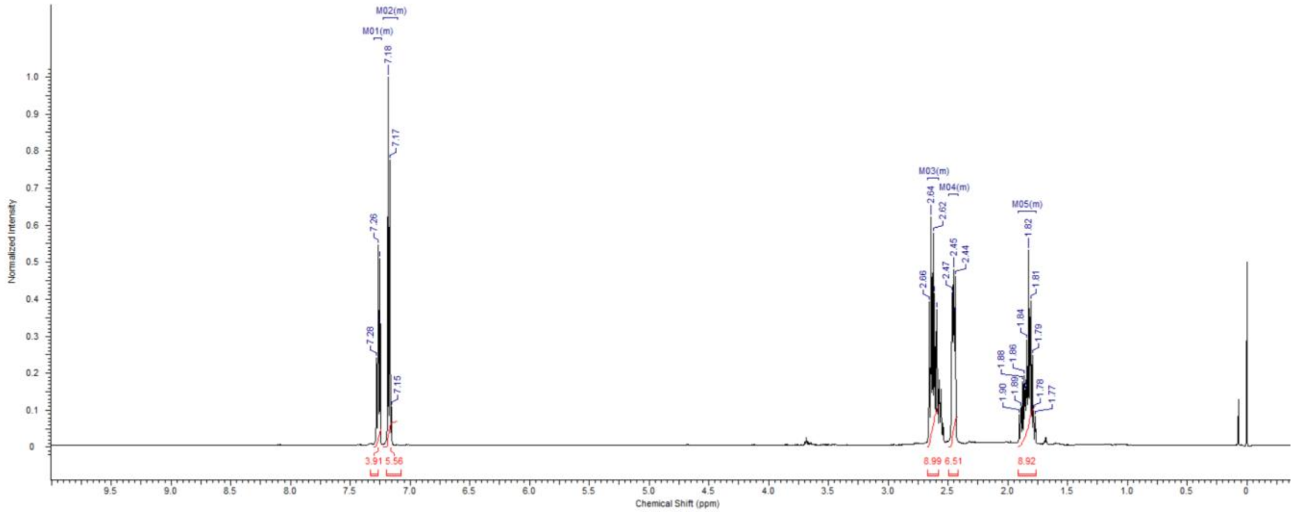

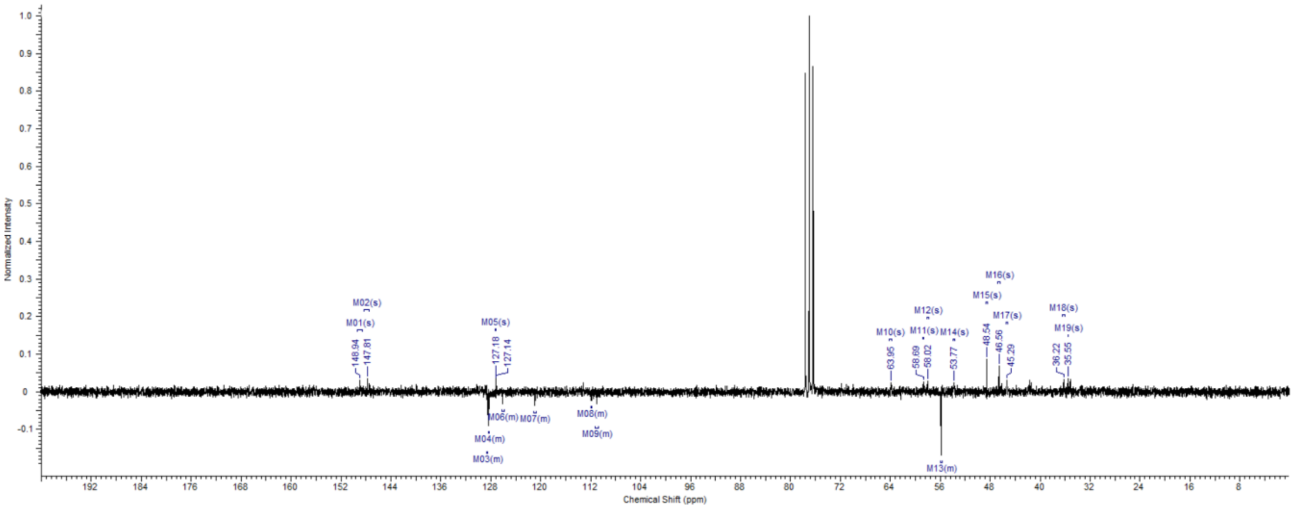

Molecular modeling analysis

Table S1. Docking Score values of the 19 compounds in the presence of S1R and S2R. The Docking score values correspond to the average values of the compounds' stereoisomers.

Compound	D-Score* S1R	D-Score* S2R
**7b**	-8.78	-8.10
**8a**	-8.53	-8.02
**8b**	-9.41	-8.24
**9a**	-9.47	-7.50
**9b**	-9.29	-8.05
**9c**	-10.07	-8.35
**9d**	-9.63	-8.05
**11b**	-8.91	-9.04
**13a**	-9.13	-8.44
**13b**	-9.77	-8.88
**14a**	-9.75	-8.76
**14b**	-9.82	-9.18
**15a**	-11.16	-7.74
**15b**	-10.19	-7.42
**15d**	-10.10	-8.36
**15e**	-10.42	-8.60
**16a**	-9.81	-8.93
**16b**	-10.37	-8.92
**16c**	-10.25	-8.39

**Docking Scores values are expressed in kcal/mol.*

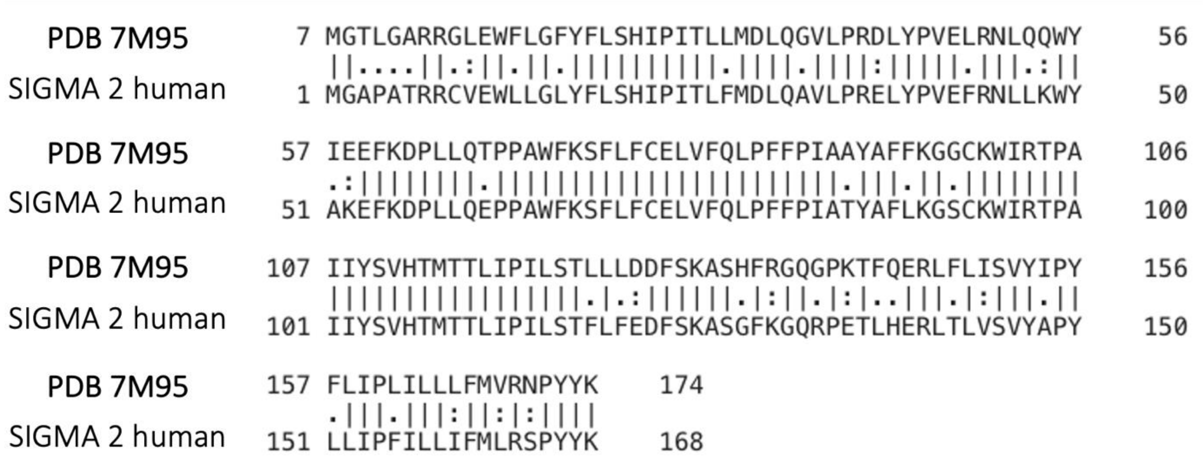

Figure S1. Sequence alignment between human and bovine S2R (PDB model 7M95). The single and the double dots indicate the different and similar residues between the human and bovine S2R sequences, respectively.

Table S2. Count and percentage of actives in top N% of results for S1R.

**% Results**	1	2	5	10	20
**# Actives**	1	1	3	3	7
**% Actives**	6.7	6.7	20.0	20.0	46.7

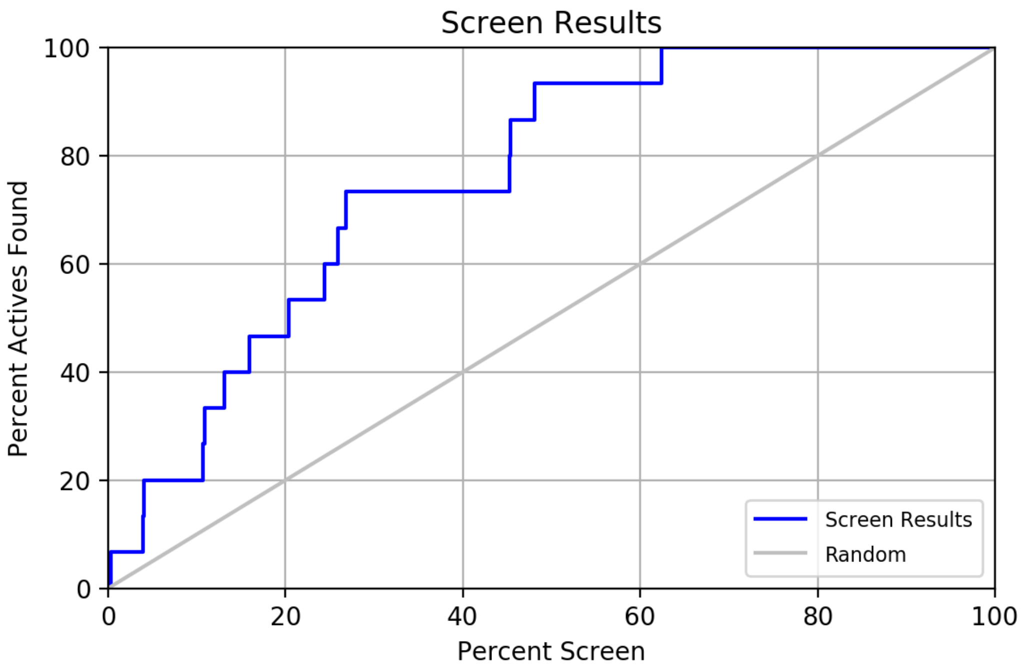

Figure S2. Percent screen plot for S1R. The percentage of the processed compounds and the percentage of the identified active compounds are reported on the abscissa and on the ordinate axes, respectively.

Table S3. Count and percentage of actives in top N% of results for S2R.

**% Results**	1	2	5	10	20
**# Actives**	5	7	11	13	15
**% Actives**	33.3	46.7	73.3	86.7	100.0

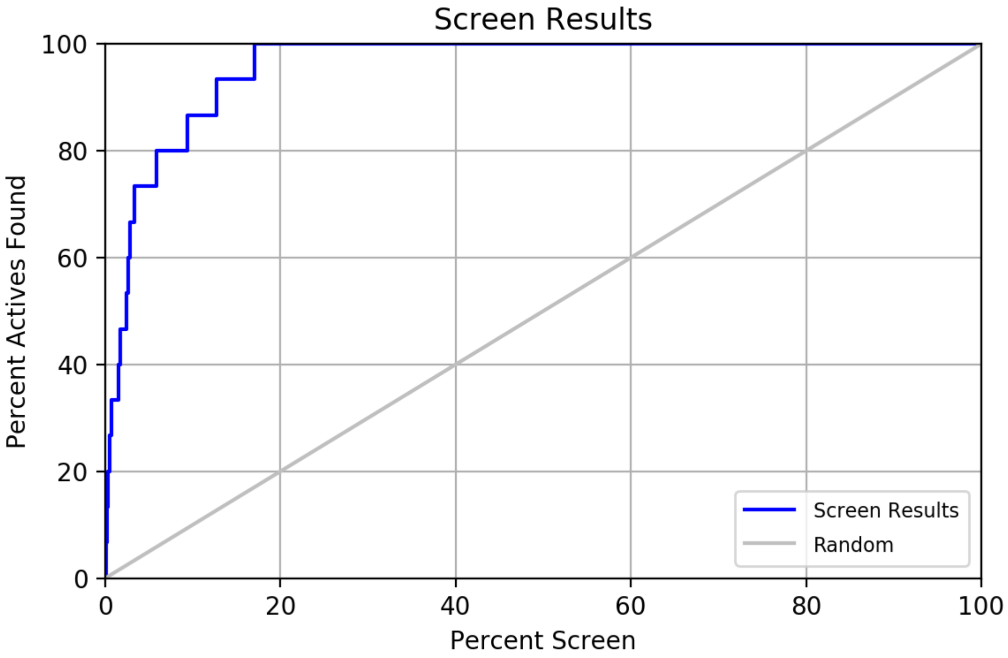

Figure S3. Percent screen plot for S2R. The percentage of the processed compounds and the percentage of the identified active compounds are reported on the abscissa and on the ordinate axes, respectively.
